# CCDC174 deficiency impaired human fertility by affecting the alternative splicing of maternal mRNAs

**DOI:** 10.1038/s44321-026-00448-y

**Published:** 2026-05-12

**Authors:** Weijie Wang, Zhiqi Pan, Huixia Jing, Rong Shi, Ling Wu, Jinjie Wang, Biaobang Chen, Jian Mu, Zhihua Zhang, Tianyu Wu, Qiaoli Li, Juanzi Shi, Yanping Kuang, Lin He, Lei Wang, Qing Sang

**Affiliations:** 1 International Peace Maternity and Child Health Hospital, School of Medicine, Shanghai Jiao Tong University, Shanghai, China; 2 Institute of Pediatrics, Children’s Hospital of Fudan University, The Institutes of Biomedical Sciences, State Key Laboratory of Genetic Engineering, Fudan University, Shanghai, China; 3 Reproductive Medicine Center, Shaanxi Maternal and Child Care Service Center, Xi’an, China; 4 Department of Assisted Reproduction, Shanghai Ninth People’s Hospital, Shanghai Jiao Tong University School of Medicine, Shanghai, China; 5 Bio-X Center, Key Laboratory for the Genetics of Developmental and Neuropsychiatric Disorders, Ministry of Education, Shanghai Jiao Tong University, Shanghai, China; 6 Shanghai Academy of Natural Sciences (SANS), Fudan University, Shanghai, China

**Keywords:** Genetics, Gene Therapy & Genetic Disease

## Abstract

Precise regulation of alternative splicing (AS) of maternal mRNAs is crucial for maintaining mRNA homeostasis and for acquiring oocyte competence. However, the regulatory factors and mechanisms of AS regulating oocyte competence and human fertility remain largely unknown. Here, we identified biallelic variants in *CCDC174* that cause human oocyte competence defects and female infertility. Oocyte-specific knockout of *Ccdc174* resulted in oocyte maturation arrest and female infertility in mice, and transcriptomic and proteomic analyses indicated that deletion of *Ccdc174* disrupted mRNA and protein homeostasis as well as AS in oocytes. Importantly, we found that CCDC174 interacted with the splicing machinery-related PRP19/CDC5L complex, and loss of CCDC174 led to aberrant activation of the expression of these complex members in oocytes. In addition, in vitro studies indicated that patient-derived variants impaired the expression of CCDC174 and its binding to RNAs or CDC5L. Taken together, our study not only show that CCDC174 is a novel AS regulator that maintains mRNA homeostasis and oocyte competence, but also decipher the critical role of *CCDC174* deficiency in the pathogenesis of female infertility.

## Synopsis

Five *CCDC174* variants were identified in five infertile females with oocyte competence defects, and were found to follow a recessive inheritance pattern. In vitro and in vivo studies elucidated the physiological and pathological roles of CCDC174 in oocyte development and female reproduction.

- Oocyte‑specific knockout of *Ccdc174* in mice results in oocyte maturation arrest and female infertility.
- *Ccdc174* deletion disrupts RNA and protein homeostasis, as well as alternative splicing in oocytes.
- CCDC174 regulates alternative splicing of maternal mRNAs by interacting with the PRP19/CDC5L complex.
- Patient-derived variants reduced CCDC174 expression and its interaction with RNAs or CDC5L.

## Abstract (web summary)

Five *CCDC174* variants were identified in five infertile females with oocyte competence defects, and were found to follow a recessive inheritance pattern. In vitro and in vivo studies elucidated the physiological and pathological roles of CCDC174 in oocyte development and female reproduction.

## Graphical abstract

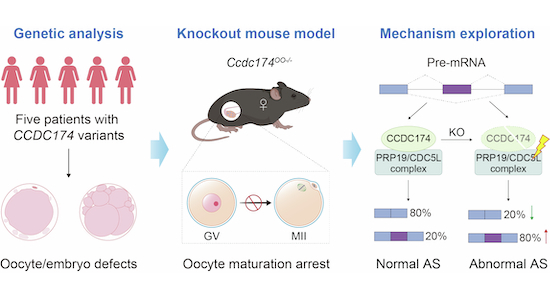

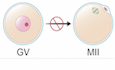

The paper explained **Problem**

Oocyte competence defects cause recurrent IVF/ICSI failure and female infertility in clinical practice. Recent studies have highlighted the contribution of genetic factors to oocyte competence defects. However, the diagnostic yield of reported pathogenic genes is only ~13.2%, and the genetic causes and pathogenic mechanisms remain unclear in the majority of patients.

**Results**

We identified homozygous or compound heterozygous variants in *CCDC174* in five independent infertile patients characterized by oocyte competence defects. To date, our understanding of CCDC174 function remains limited, and its role in oocyte development is entirely unknown. We found that CCDC174 was highly and specifically expressed in oocytes. Oocyte-specific knockout of *Ccdc174* resulted in oocyte maturation arrest and female infertility in mice. Transcriptomic and proteomic analyses showed that *Ccdc174* deletion disrupts RNA and protein homeostasis, as well as alternative splicing in oocytes. Mechanistic studies demonstrated that CCDC174 regulates alternative splicing of maternal mRNAs by interacting with the splicing machinery-related PRP19/CDC5L complex. Finally, in vitro functional experiments confirmed that patient-derived variants reduced CCDC174 expression and its interaction with RNAs or CDC5L. These results confirm that CCDC174, as a novel maternal effector, regulates alternative splicing of maternal mRNAs and plays a critical role in oocyte development and female reproduction.

**Impact**

Our study reveals that CCDC174 deficiency cause oocyte competence defects and female infertility, and elucidates the physiological and pathological mechanisms of CCDC174 in oocyte development. These findings not only expand our understanding of the regulatory mechanisms of maternal mRNA splicing in oocytes, but also provide a novel genetic diagnostic marker for female infertility patients.

## Introduction

Oocyte competence is one of the decisive factors that influence female fertility (Conti and Franciosi, 2018). In clinical practice, defects in oocyte competence cause female infertility and failure of in vitro fertilization (IVF) and intracytoplasmic sperm injection (ICSI), manifested by oocyte maturation arrest, fertilization failure or embryonic arrest (Sang et al, 2023). Recent studies have illuminated the contribution of genetic factors to oocyte competence defects and female infertility (Chen et al, 2025). However, the reported genes can only account for ~13.2% of corresponding patients (Chen et al, 2025), letting majority of patients remain unexplained.

The production and homeostatic storage of maternal mRNAs is a prerequisite for the acquisition of oocyte competence. Of the reported mutant genes, six of them are involved in regulating the dynamics of maternal mRNAs in oocytes. For example, PATL2 regulates the transcription and stability of maternal mRNA (Zhang et al, 2023), with its deficiency cause oocyte arrest at the germinal vesicle (GV) stage (Chen et al, 2017; Christou-Kent et al, 2018). PABPC1L dysfunction leads to oocyte competence defects by impairing poly(A) tail-modulated translational activation of maternal mRNAs (Wang et al, 2023). TBPL2 and LHX8 are oocyte-specific transcription factors, and their loss-of-function result in oocyte maturation arrest (Yang et al, 2021; Zhao et al, 2022). Impairment in the maternal mRNA degradation regulatory factors BTG4 and ZFP36L2 cause zygotic cleavage failure and embryonic arrest, respectively (Zheng et al, 2022; Zheng et al, 2020). These genetic studies highlight the critical role of transcription and stability of maternal mRNAs in maintaining human oocyte competence and female reproduction. Furthermore, knockout mouse studies demonstrated that alternative splicing (AS), a key step in post-transcriptional mRNA processing, is essential for maintaining maternal mRNA homeostasis and acquiring oocyte competence (Do et al, 2018; Yu et al, 2021; Zhang et al, 2022a; Zhang et al, 2022b). However, the regulatory factors and mechanisms governing the AS of maternal mRNA remain largely unknown, and no causal genes encoding AS regulatory factors have been reported to cause human oocyte competence defects and infertility.

CCDC174 (Coiled-coil domain-containing 174) contains two coiled-coil domains, and are highly conserved in vertebrates. In neuroblastoma cells it has been shown that CCDC174 localizes to the nucleus and interacts with EIF4A3 (Volodarsky et al, 2015), while in mouse ES cells CCDC174 interacts with NRDE2 to bind with U1 snRNA (Flemr et al, 2023). In addition, knockdown of the CCDC174 ortholog in *Xenopus laevis* embryos results in aberrant neural fold closure and embryonic lethality, suggesting that CCDC174 is essential for neuronal differentiation (Volodarsky et al, 2015). Despite the above studies, until now our understanding of the functions of CCDC174 is still limited, especially its role in oocyte development and human reproduction is unclear.

In the present study, we identified pathogenic variants in *CCDC174* in five patients characterized by oocyte competence defects and female infertility. We found that CCDC174 was highly and specifically expressed in oocytes, and oocyte-specific knockout of *Ccdc174* in mice resulted in oocyte maturation arrest and female infertility. Multi-omics analysis and mechanistic studies suggested that CCDC174 regulates the AS and homeostasis of maternal mRNAs in oocytes by interacting with the PRP19/CDC5L complex that is involved with the RNA splicing machinery. Finally, we investigated the disruptive effect of patient-derived variants on protein function in vitro. Our findings suggest an important role for CCDC174 in the regulation of AS in oocytes and highlight the clinical significance of CCDC174 in human reproduction and Mendelian disease.

## Results

### Identification of pathogenic variants in *CCDC174*

**Figure 1:**
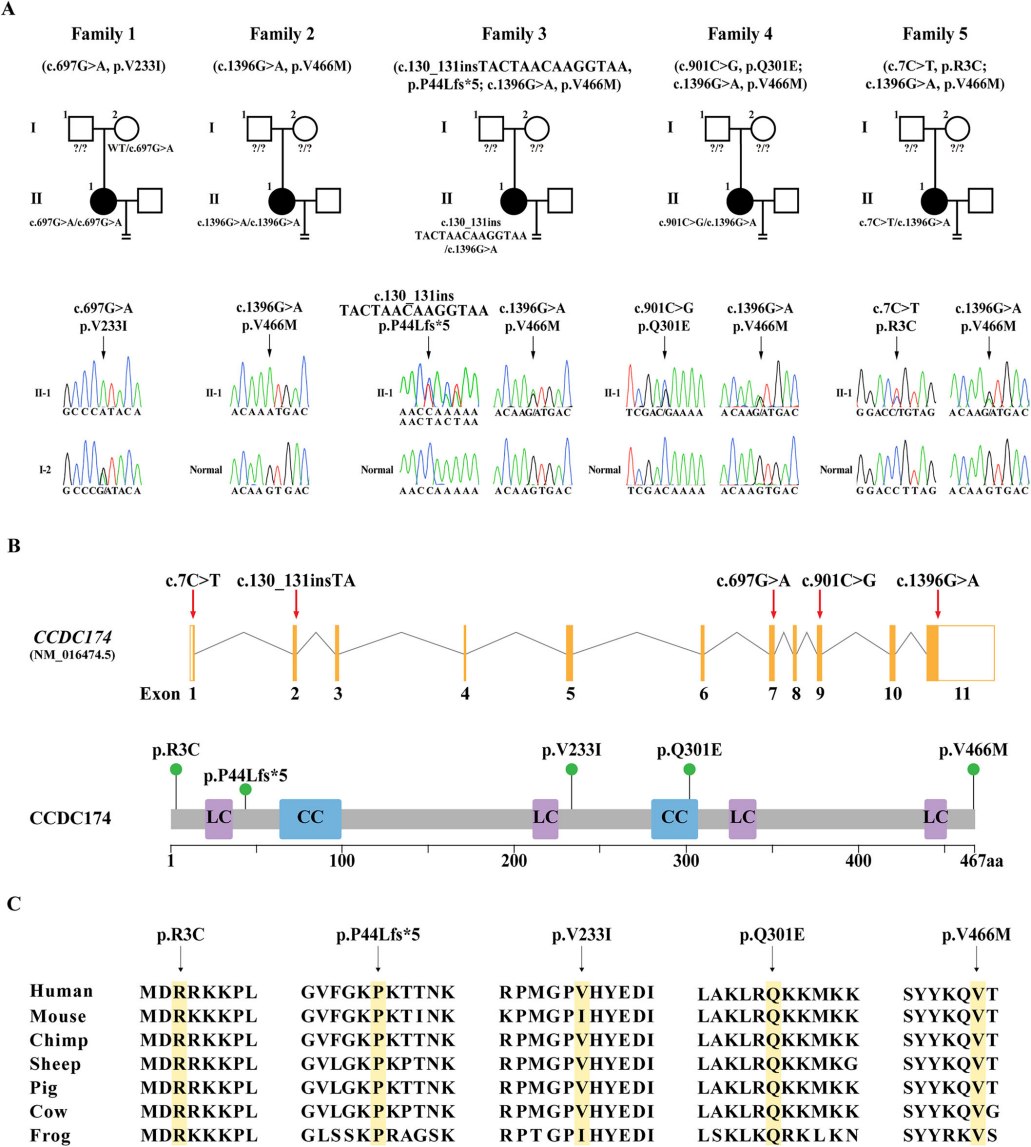
Identification of pathogenic variants in *CCDC174.* (**A**) Pedigrees of the five affected families. Sanger sequencing confirmation is shown below the pedigrees. Squares indicate male members, circles indicate female members, black circles indicate the affected individuals, question marks indicate unavailable DNA samples, and equal signs (=) indicate infertility. (**B**) Locations of the identified variants in *CCDC174* exons and the protein structure of CCDC174. LC low complexity, CC coiled coil. (**C**) Conservation analysis of the mutant amino acids in seven different species.

To identify the potential genetic factors underlying oocyte competence defects and female infertility, we performed a case-control association analysis combined with a gene set enrichment analysis on WES data from 3627 cases and 2868 controls (Chen et al, 2025). We identified homozygous or compound heterozygous variants in *CCDC174* (GenBank: NM_016474.5) in five independent infertility patients (Fig. 1A). In contrast, no homozygous or compound heterozygous variants in *CCDC174* were found in 2868 controls. The affected individual in family 1 carried homozygous variant c.697 G > A (p.V233I). The affected individual in family 2 carried homozygous variant c.1396 G > A (p.V466M). The affected individuals in families 3, 4, and 5 all carried the recurrent variant c.1396 G > A (p.V466M) combined with c.130_131insTACTAACAAGGTAA (p.P44Lfs*5), c.901 C > G (p.Q301E), and c.7 C > T (p.R3C), respectively. All the identified variants were confirmed through Sanger sequencing and suggested a recessive inheritance pattern (Fig. 1A). Detailed information about the variants, including the genomic position, predicted damaging effect, and frequency, is provided in Table 1. CCDC174 contains 11 exons encoding a 467 amino acid protein, and the locations of the variants in the gene and protein structure are shown in Fig. 1B. Most corresponding amino acids of these patient-derived variants are conserved across different species (Fig. 1C). Taken together, all the genetic evidence strongly suggests the potential role of *CCDC174* in human oocyte competence defects and female infertility.

**Table 1:** *CCDC174* variants identified in the five families.

Affected families	Genomic position (Chr 3)	cDNA change	Protein change	Variant type	SIFT^A^	PPH2^A^	MutTas^A^	GnomAD^B^
Family 1	14708427	c.G697A	p.V233I	Missense	T	B	B	0.0004465
Families 2, 3, 4, 5	14712693	c.G1396A	p.V466M	Missense	T	P	D	0.000636
Family 3	14696020	c.130_131insTACTAACAAGGTAA	p.P44Lfs*5	Frameshift	NA	NA	D	NA
Family 4	14709637	c.C901G	p.Q301E	Missense	T	P	D	0.000004112
Family 5	14693350	c.C7T	p.R3C	Missense	D	P	D	0.00005212

*T* tolerated, *B* benign, *P* probably damaging, *D* damaging or disease causing, *NA* not available.

^A^Mutation assessment by SIFT and PolyPhen-2 (PPH2), and MutationTaster (MutTas).

^B^Frequency of corresponding variants in the gnomAD databases.

### Clinical characteristics of the affected individuals

All five affected individuals with *CCDC174* variants had been diagnosed with primary infertility of unknown cause for several years despite their having had normal menstrual cycles. Their male partners had normal sperm counts and normal sperm morphologic features and motility. They all underwent several failed IVF/ICSI attempts.

The affected individual in family 1 underwent one failed IVF attempt and five failed ICSI attempts. A total of 29 oocytes were retrieved, among which 21 were mature metaphase II (MII) oocytes. However, only 8 oocytes were successfully fertilized and cleaved. Eventually, four viable embryos were obtained on day 3, but no pregnancy was achieved after embryo transfer (Table 2).

**Table 2:** Clinical characteristics of affected individuals and their retrieved oocytes.

Individual	Age (years)	Duration of infertility (years)	IVF/ICSI cycles	Total oocytes	Immature oocytes	MII oocytes	Normal fertilized oocytes	Normal cleaved embryos	Viable embryos on day 3	Outcomes
II-1 in Family 1	28	4	1st IVF	2	0	2	0	0	0	Four viable embryos were frozen on day 3 and failed to establish pregnancy after transfer.
			1st ICSI	3	0	3	2	1	1	
			2nd CSI	8	2	6	4	3	1	
			3rd ICSI	2	0	2	1	1	1	
			4th ICSI	8	3	5	3	3	1	
			5th ICSI	6	3	3	0	0	0	
II-1 in Family 2	32	NA	1st IVF	9	0	9	2	2	2	Four viable embryos were frozen on day 3 and failed to establish pregnancy after transfer.
			2nd IVF	6	0	6	2	2	2	
II-1 in Family 3	30	5	1st IVF	5	1	4	3	3	1	Seven viable embryos were frozen on day 3. The remaining 13 embryos were further cultured, and one blastocyst was obtained on day 5. This blastocyst established a pregnancy after transfer and resulted in a successful delivery.
			2nd IVF	7	0	7	6	6	5	
			1st ICSI	6	1	5	4	4	4	
			3rd IVF	15	2	13	12	12	10	
II-1 in Family 4	27	3	1st ICSI	4	NA	NA	NA	NA	0	One viable embryo developed into a blastocyst on day 5. However, it failed to establish a pregnancy after transfer.
			2nd ICSI	10	6	4	2	2	1	
			3rd ICSI	13	6	7	1	1	0	
II-1 in Family 5	28	4	1st IVF	9	0	9	0	0	0	/

*IVF* in vitro fertilization, *ICSI* intracytoplasmic sperm injection, *MII* metaphase II.

The affected individual in family 2 underwent two failed IVF attempts. A total of 15 mature oocytes were retrieved. After IVF, only four of these oocytes were fertilized and developed into viable embryos on day 3. However, no pregnancy was achieved following the transfer of these embryos (Table 2).

The affected individual in family 3 underwent three failed IVF attempts and one failed ICSI attempt. A total of 25 fertilized oocytes were obtained. All these fertilized oocytes underwent cleavage, but most of the embryos arrested at an early stage, with only one developing into a blastocyst. This blastocyst established a pregnancy after transfer and resulted in a successful delivery (Table 2).

The affected individual in family 4 underwent three failed ICSI attempts. In the first cycle, four oocytes were retrieved, and no viable embryos were obtained after ICSI. In her subsequent two ICSI cycles, a total of 23 oocytes were retrieved, among which 11 were MII oocytes. After ICSI, only three fertilized oocytes were obtained, one of which developed into a blastocyst. However, this blastocyst failed to result in a pregnancy after transfer (Table 2).

The affected individual in family 5 underwent one failed IVF attempt. A total of nine oocytes were retrieved, all of which were mature MII oocytes. However, all these oocytes failed to fertilize following IVF (Table 2).

Overall, the clinical records of patients with *CCDC174* variants showed that all oocytes retrieved from them exhibited oocyte competence defects, manifested as oocyte maturation arrest, fertilization failure, or embryonic arrest (Table 2).

### Nuclear localization and high expression of CCDC174 in mouse oocytes and early embryos

**Figure 2:**
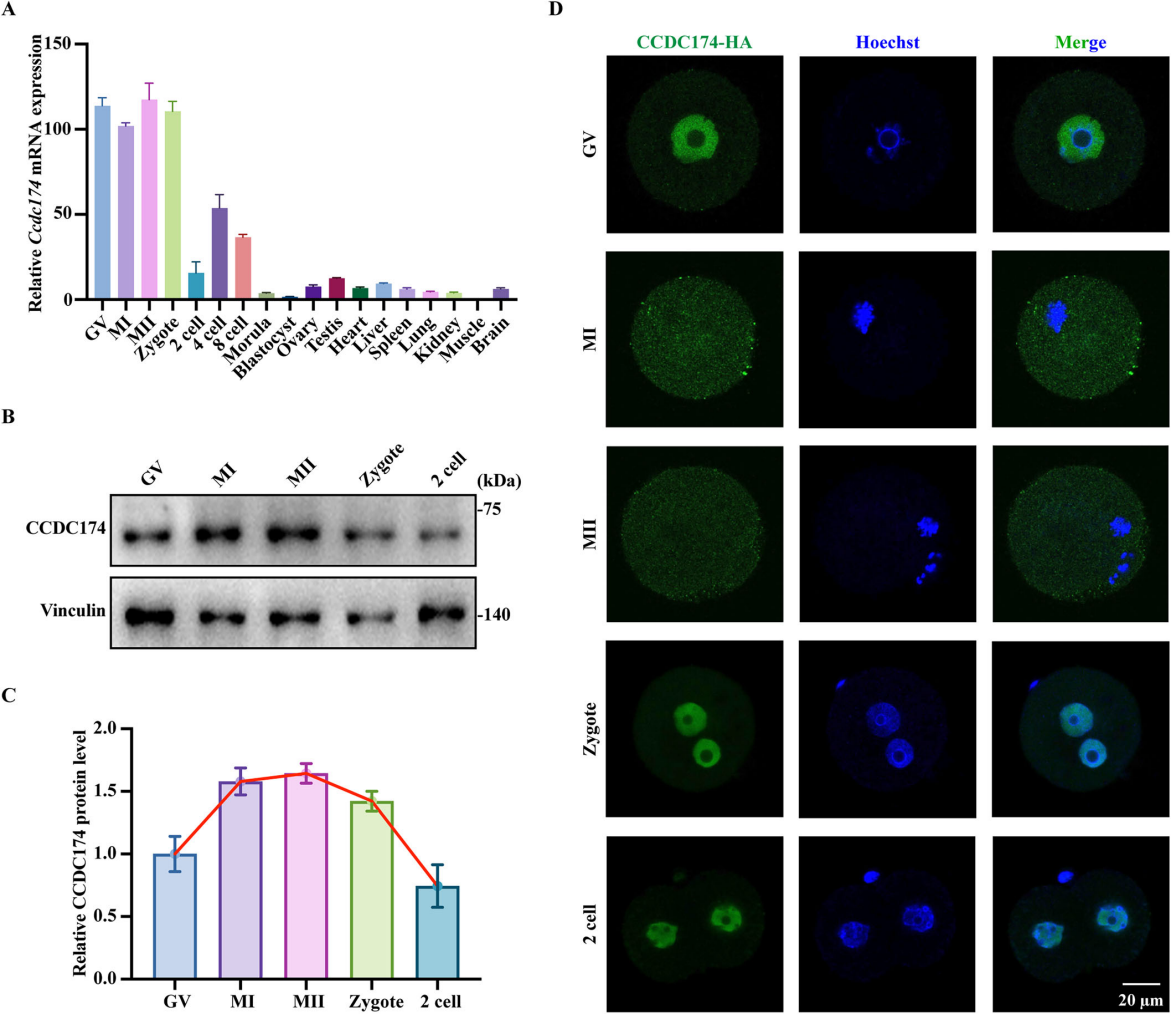
The expression and localization patterns of CCDC174 in oocytes and early embryos. (**A**) qRT-PCR results showing the relative expression levels of *Ccdc174* mRNA in different stages of mouse oocytes, early embryos, and various tissues. *n* = 4 biological replicates. Error bars represent the mean and SD. (**B**) Immunoblot results showing the protein levels of CCDC174 in different stages of mouse oocytes and early embryos. (**C**) Quantitation of CCDC174 protein levels in different stages of mouse oocytes and early embryos. *n* = 4 biological replicates. Data are shown as mean and SD. (**D**) Immunofluorescence results showing the localization of HA-CCDC174 in different stages of mouse oocytes and early embryos. Scale bar = 20 μm. Source data are available online for this figure.

Since the physiological function of CCDC174 in the reproductive process remains completely unknown, we first detected the expression of *Ccdc174* in different stages of mouse oocytes, in early embryos, and in various tissues by qRT-PCR. The results indicated that *Ccdc174* was highly expressed in GV, metaphase I (MI), and MII oocytes, as well as in zygotes, whereas it exhibited low expression in early embryos and somatic tissues (Fig. 2A). Protein level analysis showed that CCDC174 increases gradually during oocyte maturation and decreases following fertilization (Fig. 2B,C). Furthermore, immunofluorescence results showed that CCDC174 was specifically localized in the nucleus of GV oocytes, whereas it was distributed throughout the cytoplasm in MI and MII oocytes (Fig. 2D). After fertilization, CCDC174 reappeared in the nucleus of zygotes and 2-cell embryos. These expression and localization patterns suggest that CCDC174 may function in the nucleus and play a potential role in oocyte maturation.

### *Ccdc174* depletion causes female infertility in mice by disturbing oocyte maturation

**Figure 3:**
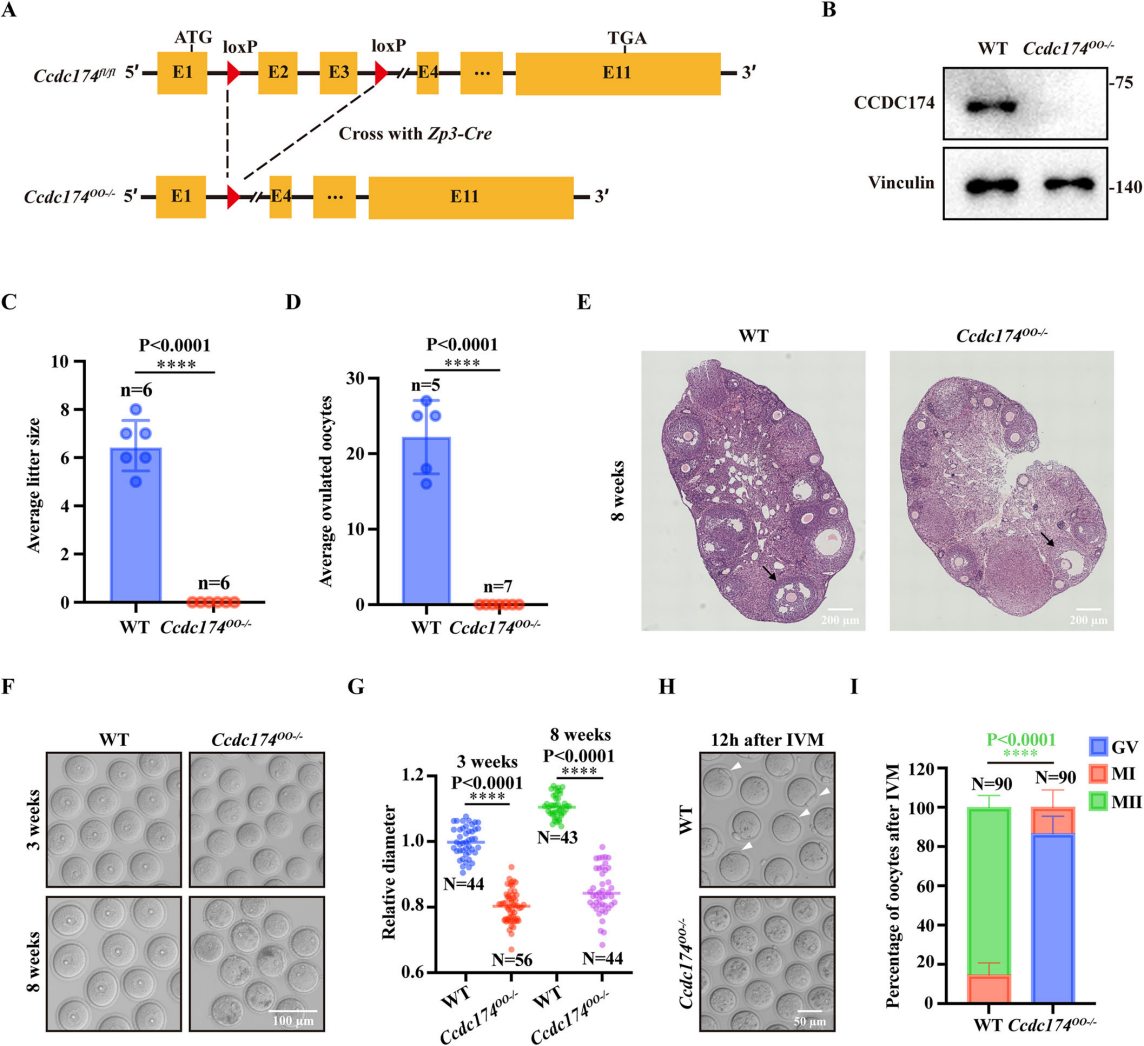
CCDC174 deficiency results in oocyte competence defects and female infertility in mice. (**A**) The strategy employed to construct oocyte-specific knockout mice (*Ccdc174*^*OO–/–*^). The deletion of Exons 2 and Exons 3 led to a loss-of-function frameshift mutation in *Ccdc174*. (**B**) Immunoblotting of CCDC174 in GV oocytes of WT and *Ccdc174*^*OO–/–*^ mice. Vinculin was used as the loading control. (**C**) The reproductive capacity of 8-week-old WT and *Ccdc174*^*OO–/–*^ female mice. *n* = 6 for each group. The statistics are analyzed by unpaired two-tailed Student’s *t* test. Data are shown as mean and SD. ****P  <  0.0001. WT vs *Ccdc174*^*OO–/–*^, *P* = 3.1 × 10^−8^. (**D**) Numbers of oocytes collected from the oviducts of 8-week-old WT and *Ccdc174*^*OO–/–*^ mice after superovulation. *n* ≥ 5 for each group. The statistics are analyzed by unpaired two-tailed Student’s *t* test. Da*t*a are shown as mean and SD. ****P  <  0.0001. WT vs *Ccdc174*^*OO–/–*^, *P* = 2.3  × 10^−7^. (**E**) Histological sections of ovaries from 8-week-old WT and *Ccdc174*^*OO–/–*^ mice were stained with hematoxylin and eosin. Black arrows indicate antral follicles. Scale bars = 200 μm. (**F**) Representative images of 3-week and 8-week-old WT and *Ccdc174*^*OO–/–*^ oocytes. Scale bars = 100 μm. (**G**) The quantitative statistical results of oocyte diameter from WT and *Ccdc174*^*OO–/–*^ mice. The number of analyzed oocytes is indicated (*N*). The statistics are analyzed by unpaired two-tailed Student’s *t* test. Data are shown as mean and SD. ****P  <  0.0001. WT vs *Ccdc174*^*OO–/–*^, 3 weeks, *P* = 6.3 × 10^−38^; 8 weeks, *P* = 4.3 × 10^−37^. (**H**) Representative images of oocytes after 12 h of in vitro maturation (IVM). Scale bar = 50 μm. White arrowheads indicate the first polar body. (**I**) The proportion of oocytes at different stages after IVM in 3-week-old mice with PMSG for 46 h. *n*  =  3 biological replicates. The *n*umber of analyzed oocytes is indicated (*N*). The statistics are analyzed by unpaired two-tailed Student’s *t* test. Data are shown as mean and SD. ****P  <  0.0001. Source data are available online for this figure.

Next, to explore the function of CCDC174 in oocyte competence and female fertility, we constructed in vivo mouse model. Given that homozygous *Ccdc174* knockout mice are embryonic lethal (MGI: 2444652), we generated an oocyte-specific *Ccdc174* knockout mouse (*Ccdc174*^*OO–/–*^) by crossing *Ccdc174*^fl/fl^ mice with transgene *Zp3-Cre* mice (Fig. 3A). Immunoblotting confirmed the complete loss of CCDC174 protein in *Ccdc174*^*OO–/–*^ oocytes (Fig. 3B). The fertility assessment showed that no pups were born to *Ccdc174*^*OO–/–*^ females when crossed to WT males for at least 3 months (Fig. 3C), indicating that *Ccdc174*^*OO–/–*^ female mice were completely infertile.

To address why *Ccdc174*^*OO–/–*^ female mice were infertile, we first performed superovulation experiment, but no oocytes were ovulated from *Ccdc174*^*OO–/–*^ female mice (Fig. 3D). To determine the causes of superovulation failure, we conducted histological analyses of ovarian tissues from 8-week-old mice. Still, there was no difference in ovarian size, follicular growth, or the formation of antral follicles between WT and *Ccdc174*^*OO−/−*^ mice (Fig. 3E; Appendix Fig. S1A,B). Then, we tried to collect GV oocytes from the ovaries, and found that although similar number of GV oocytes can be retrieved from both groups, the diameter of *Ccdc174*^*OO–/–*^ oocytes was smaller than that of WT oocytes (Fig. 3F,G). To evaluate the developmental competence of *Ccdc174*-deleted oocytes, we cultured GV oocytes in vitro and found that most GV oocytes from *Ccdc174*^*OO-/-*^ mice failed to mature, and arrested at the GV stage (Fig. 3H,I). Taken together, these results implied that CCDC174 deficiency severely impairs oocyte competence and female infertility.

### CCDC174 deficiency disrupted the homeostasis of mRNAs and proteins in oocytes

**Figure 4:**
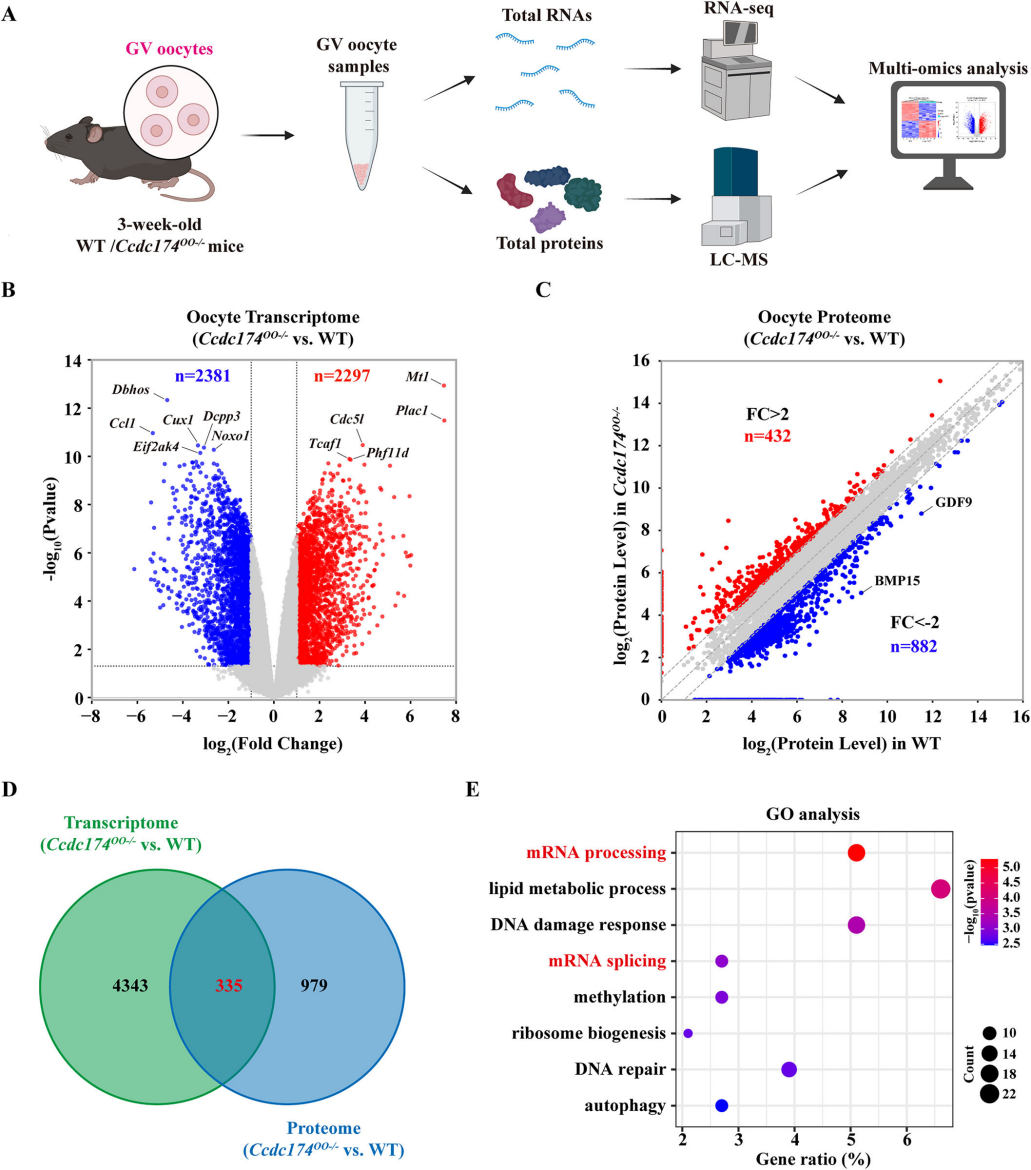
Transcriptomic and proteomic analysis in WT and *Ccdc174*^*OO–/–*^ oocytes. (**A**) Schematic illustration of the collection of GV oocytes from 3-week-old WT and *Ccdc174*^*OO–/–*^ mice for transcriptome and proteome analyses. (**B**) Volcano plot depicting differentially expressed genes between WT and *Ccdc174*^*OO–/–*^ GV oocytes. DESeq2 was used for differential gene expression analysis, *P* value was calculated by the Wald test. (**C**) Scatter plot depicting differentially abundant proteins between WT and *Ccdc174*^*OO–/–*^ GV oocytes. Differential protein abundance was evaluated using a *t* test. FC fold change. (**D**) Venn diagram showing the shared differentially expressed components in both the transcriptome and proteome. (**E**) GO analysis of shared differentially expressed components in *Ccdc174*^*OO–/–*^ vs. WT oocytes. GO analysis was performed using clusterProfiler R package based on the hypergeometric test.

To elucidate the mechanism of impaired oocyte competence in *Ccdc174*^*OO–/–*^ mice, we performed transcriptomic and proteomic analyses in WT and *Ccdc174*-deleted oocytes (Fig. 4A; Appendix Fig. S2A,B). Transcriptome analysis revealed that 2,381 transcripts were downregulated and 2297 transcripts were upregulated in *Ccdc174*-deleted oocytes compared with WT oocytes (Fig. 4B; Dataset EV1). The transcriptome data were validated by qRT-PCR of mRNA levels for selected genes (Appendix Fig. S2C). Meanwhile, proteomic analysis showed that 432 proteins were upregulated and 882 proteins were downregulated in *Ccdc174*-deleted oocytes compared with WT oocytes (Fig. 4C; Dataset EV2). The downregulated proteins included the oocyte-specific growth factors GDF9 and BMP15 (Fig. 4C; Appendix Fig. S2D), both of which are crucial for follicle development and oocyte maturation (Kristensen et al, 2022). We integrated the transcriptome and proteome data and found 335 shared differentially expressed components in both the transcriptome and the proteome (Fig. 4D). Gene Ontology (GO) analysis revealed that the shared components were mainly involved in mRNA processing, lipid metabolic process, DNA damage response, mRNA splicing, etc. (Fig. 4E). These results indicated that both mRNA and protein homeostasis were disrupted in *Ccdc174*-deleted oocytes.

### CCDC174 is required for the proper mRNA splicing in oocytes

**Figure EV1:**
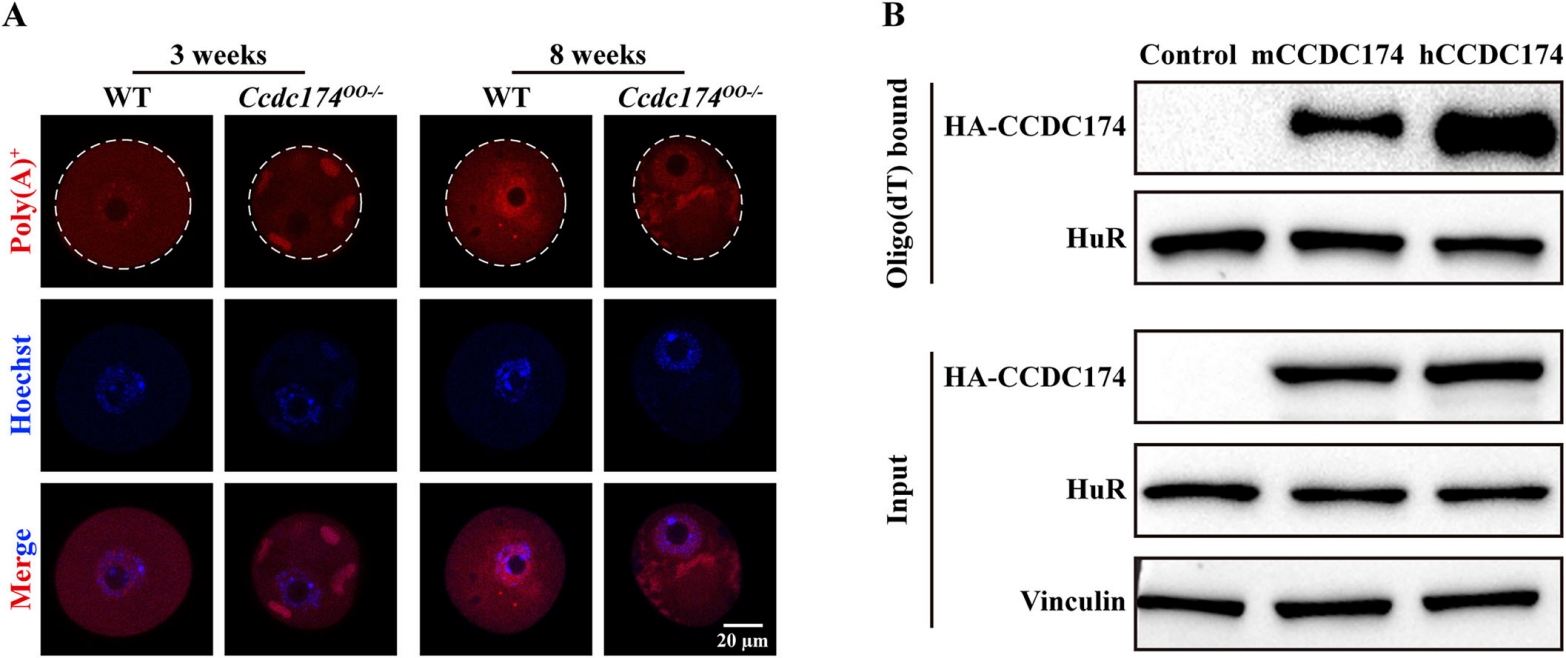
CCDC174 deficiency affects maternal mRNA localization, and human and mouse CCDC174 both bind to RNAs. (**A**) FISH of poly(A) RNAs in WT and *Ccdc174*^*OO–/–*^ oocytes. *n* ≥ 13 for each group. Dashed borders indicate the boundary of the oocyte. (**B**) Representative immunoblots of mouse and human CCDC174 bound to poly(A) RNAs in HEK293T cells. HuR was used as internal control and Vinculin was used as the loading control. *n* = 3 biological replicates.

To investigate the causes of abnormal mRNA and protein homeostasis in *Ccdc174*-deleted oocytes, we first performed a 5-ethynyluridine (EU) incorporation assay. The results showed that the transcriptional activity was not disturbed in *Ccdc174*^*OO–/–*^ oocytes (Appendix Fig. S3A,B). Considering that CCDC174 was localized in the nucleus of oocytes (Fig. 2D) and that mRNA processing and splicing were significantly enriched terms in the GO analysis, we hypothesized that CCDC174 may be associated with mRNA splicing in oocytes. Pre-mRNA splicing in the nucleus is critical to mRNA cytoplasmic localization (Martin and Ephrussi, 2009; Palacios, 2002), our FISH results showed that maternal mRNAs were abnormally aggregated in the cytoplasm of *Ccdc174*-deleted oocytes (Fig. EV1A). In addition, the RNA pull-down assay indicated that both human and mouse CCDC174 bound to RNAs (Fig. EV1B). These results implied that CCDC174 may participate in the splicing of maternal mRNAs.

**Figure 5:**
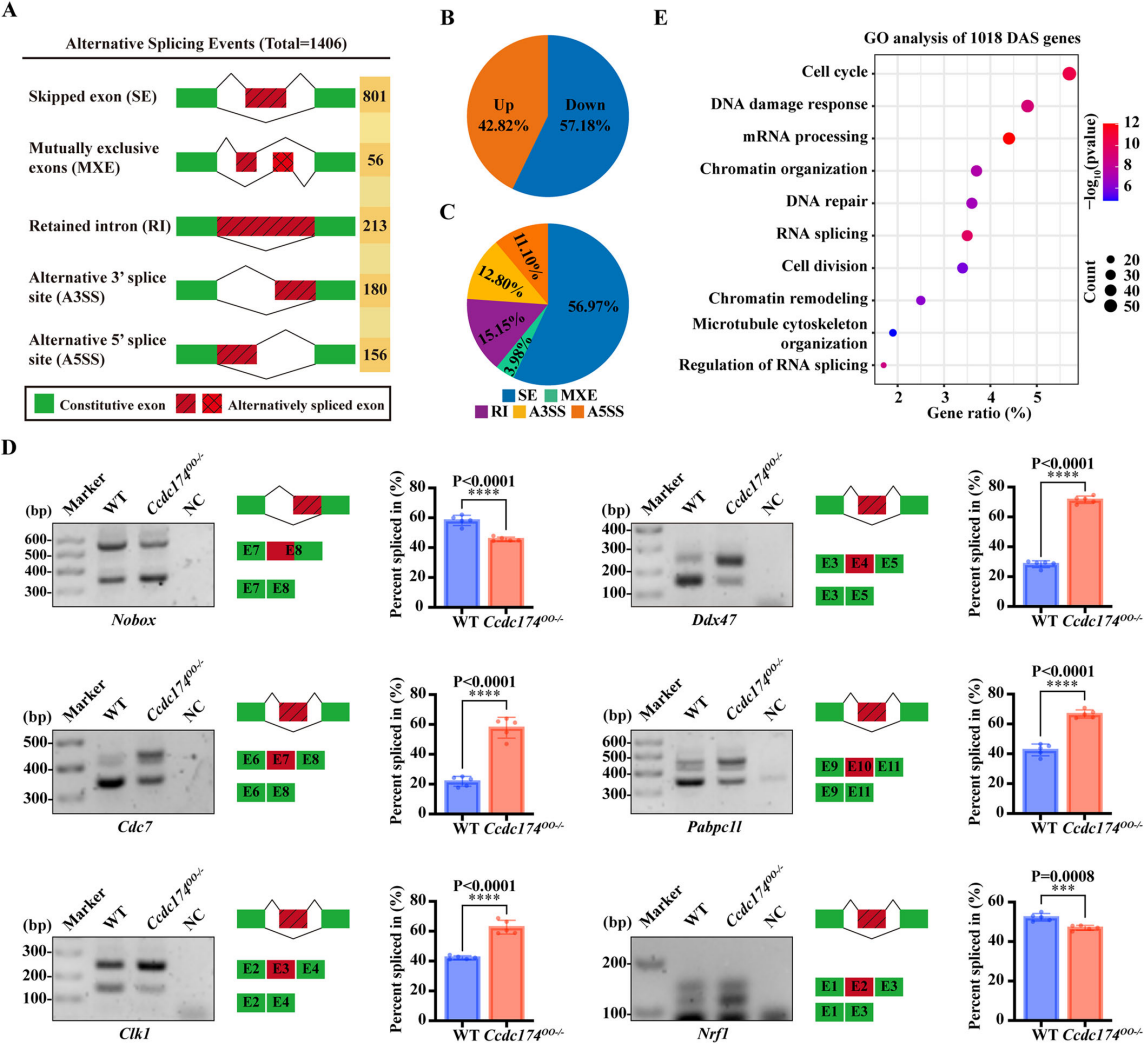
CCDC174 deficiency causes aberrant AS in mouse oocytes. (**A**) The categories and numbers of differential alternative splicing (DAS) events between WT and *Ccdc174*^*OO–/–*^ GV oocyte. (**B**) The proportion of upregulated and downregulated AS events in *Ccdc174*^*OO–/–*^ oocytes. (**C**) The proportion of different types of DAS events in *Ccdc174*^*OO–/–*^ oocytes. (**D**) Representative images of RT-PCR analyses for the DAS events in WT and *Ccdc174*^*OO–/–*^ oocytes. The middle panels represent the schematic diagram of alternatively spliced exons. The right panels show the quantification of percent spliced in (PSI). *n* = 5 biological replicates. The statistics are analyzed by unpaired two-tailed Student’s *t* test. Data are shown as mean and SD. ****P  <  0.0001. WT vs *Ccdc174*^*OO–/–*^, *Nobox*, *P* = 6.6 × 10^−5^; *Cdc7*, *P *= 6.5 × 10^−6^; *Clk1*, *P* = 1.2 × 10^−5^; *Ddx47*, *P* = 2.5 × 10^−9^; *Pabpc1l*, *P* = 3.7 × 10^−6^; *Nrf1*, *P* = 8.3 × 10^−4^. (**E**) GO analysis of genes with DAS events in *Ccdc174*^*OO–/–*^ oocytes. GO analysis was performed using clusterProfiler R package based on the hypergeometric test. Source data are available online for this figure.

To detect whether CCDC174 deficiency affects the splicing of maternal mRNAs in oocytes, we assessed the splicing events in oocytes using RNA-seq data. We found that a total of 1406 AS events were altered in *Ccdc174*^*OO–/–*^ oocytes compared with WT oocytes (Dataset EV3), including skipped exons, mutually exclusive exons, retained introns, alternative 3′ splice sites, and alternative 5′ splice sites (Fig. 5A; |PSI | >0.1, *P* value < 0.05). In *Ccdc174*^*OO–/–*^ oocytes, approximately 42.82% of AS events were upregulated, while 57.18% of AS events were downregulated (Fig. 5B). Of note, skipped exons were the predominant splicing type in oocytes (56.97%) among the AS events affected by *Ccdc174* deletion (Fig. 5C). All the affected AS events are involved in 1,018 differentially alternatively spliced (DAS) genes (Dataset EV3). We further selected six genes (*Nobox*, *Cdc7*, *Clk1*, *Ddx47*, *Pabpc1l*, and *Nrf1*) for AS event detection and confirmed abnormal AS of these genes in *Ccdc174*^*OO–/–*^ oocytes (Fig. 5D), indicating that CCDC174 deficiency causes aberrant AS in oocytes. Notably, two of these genes are essential for oocyte development and female fertility: NOBOX is an oocyte-specific transcription factor required for oocyte-specific gene expression (Rajkovic et al, 2004), and PABPC1L regulates the translational activation of maternal mRNAs (Wang et al, 2023). These represent illustrative examples within broader network-level splicing defects, rather than definitive single downstream effectors. In addition, GO analysis showed that these DAS genes are primarily enriched in biological processes that are crucial for oocyte development and maturation, including the cell cycle, DNA damage response, mRNA processing, chromatin organization, and cell division (Fig. 5E). Collectively, these results demonstrate that CCDC174 plays a crucial role in regulating AS and homeostasis of maternal mRNAs, which is essential for oocyte development and maturation.

### CCDC174 stabilizes the mRNA splicing machinery-related PRP19/CDC5L complex in oocytes

**Figure 6:**
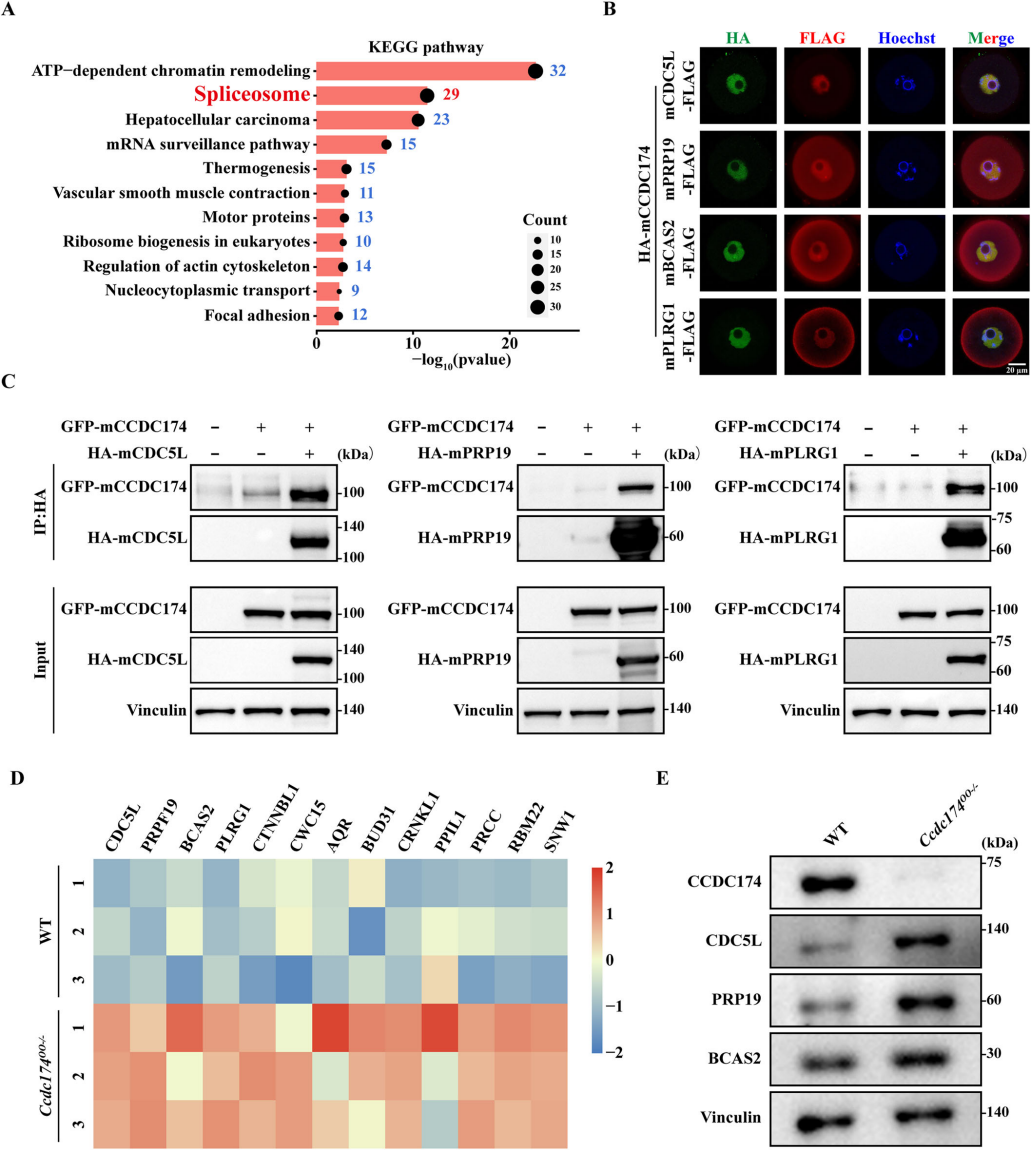
CCDC174 regulates AS in oocytes by interacting with the PRP19/CDC5L complex. (**A**) KEGG pathway analysis of the CCDC174-interacting proteins identified from the IP-MS data. KEGG pathway analysis was performed using clusterProfiler R package based on the hypergeometric test. (**B**) Co-immunofluorescence staining of mouse CCDC174 (mCCDC174) and members of the PRP19/CDC5L complex in mouse oocytes, including mouse CDC5L (mCDC5L), mouse PRP19 (mPRP19), mouse BCAS2 (mBCAS2), and mouse PLRG1 (mPLRG1). *n* ≥ 6 for each group. Scale bar =  20 μm. (**C**) Co-immunoprecipitation assays of HA-mCCDC174 and members of the PRP19/CDC5L complex in HEK293T cells. *n* = 3 biological replicates. (**D**) The heatmap shows the protein levels of the PRP19/CDC5L complex members in the proteomic data of WT and *Ccdc174*^*OO–/–*^ oocytes. (**E**) Immunoblotting analysis of the protein levels of CDC5L, PRP19, and BCAS2 in WT and *Ccdc174*^*OO–/–*^ oocytes. Vinculin was used as the loading control. *n* = 4 biological replicates. Source data are available online for this figure.

To explore how CCDC174 mediates AS in oocytes, we performed immunoprecipitation-mass spectrometry (IP-MS) to identify the proteins that interact with mouse CCDC174 (mCCDC174) in WT mouse ovaries (Appendix Fig. S4A). A total of 404 potential interacting proteins were identified and subjected to KEGG pathway analysis (Dataset EV4). The results indicated that 29 interacting proteins were enriched in the spliceosome (Fig. 6A), aligning with the aberrant mRNA splicing seen in *Ccdc174*-deleted oocytes. Further analysis revealed that the spliceosome-related proteins encompassed the four primary core proteins of the PRP19/CDC5L complex as well as numerous PRP19/CDC5L-related proteins (Appendix Fig. S4B). It has been reported that the PRP19/CDC5L complex primarily consists of four conserved core proteins—PRP19, CDC5L, PLRG1, and BCAS2—and that it plays an important regulatory role in the catalytic activity of spliceosomes (Bessonov et al, 2008; van Maldegem et al, 2015). Immunofluorescence results showed that mCCDC174 co-localized with the core members of the complex in the nucleus (Fig. 6B). Specifically, mouse CDC5L exhibited complete co-localization with mCCDC174, whereas mouse PRP19, BCAS2, and PLRG1 were localized not only in the nucleus, but also in the cytoplasm (Fig. 6B). To further verify the interaction between mCCDC174 and the complex members, we performed a Co-IP assay and found that mCCDC174 indeed interacts with mouse CDC5L, PRP19, and PLRG1 (Fig. 6C). These results demonstrate the interaction between CCDC174 and the PRP19/CDC5L complex. Of note, the protein levels of PRP19/CDC5L complex members and their related proteins were upregulated in *Ccdc174*^*OO–/–*^ oocytes compared with WT oocytes (Fig. 6D,E), suggesting CCDC174 is essential for maintaining the hemostasis of PRP19/CDC5L complex.

### Patient-derived variants in *CCDC174* impaired protein expression and its binding to RNAs or CDC5L

**Figure 7:**
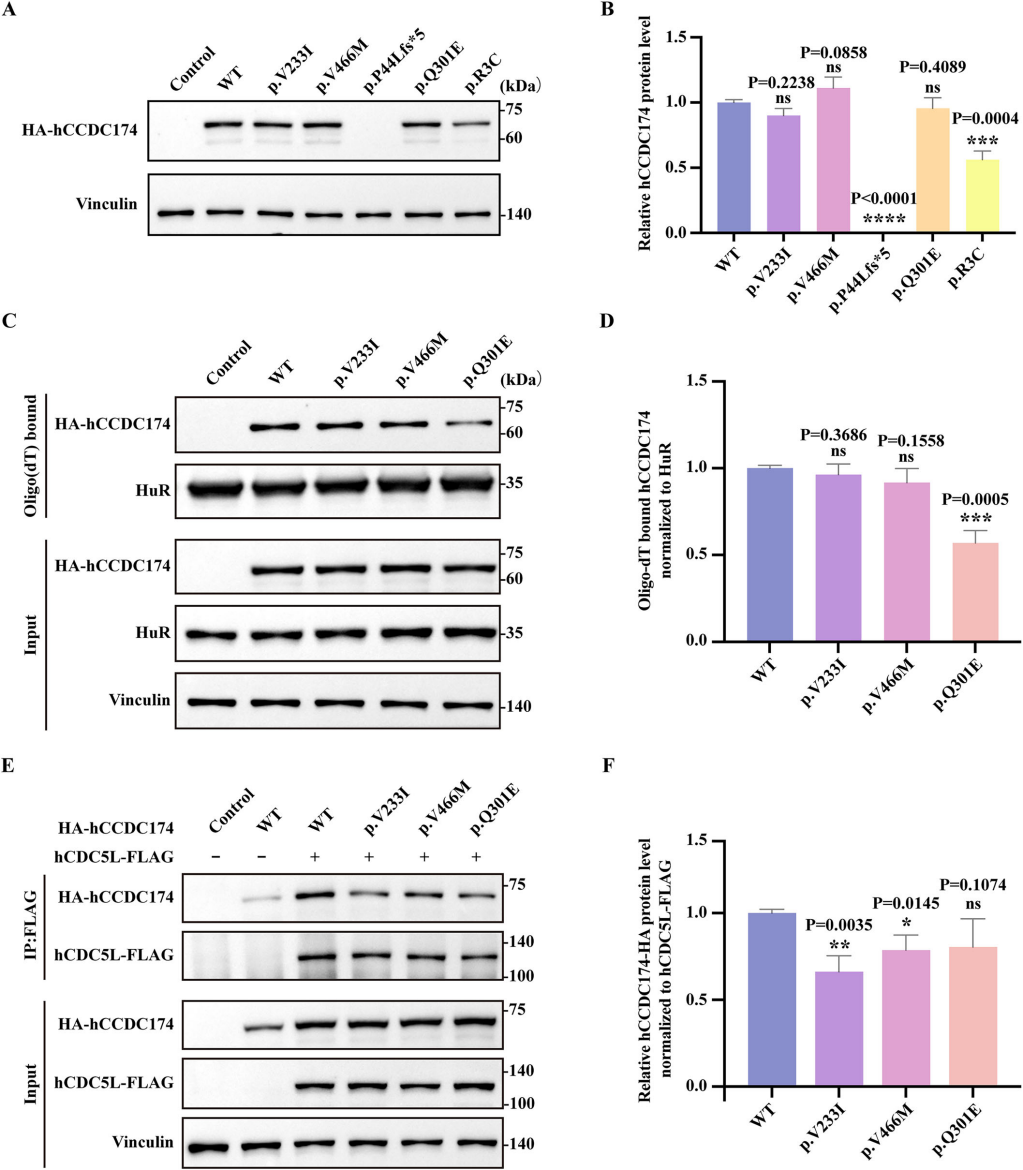
Effects of *CCDC174* variants on protein abundance and binding ability to RNA and CDC5L. (**A**) The effects of the variants on human CCDC174 (hCCDC174) expression by immunoblot in HEK293T cells. Vinculin was used as the loading control. (**B**) Quantitation of the WT and mutant hCCDC174 protein levels. *n* = 3 biological replicates. The statistics are analyzed by unpaired two-tailed Student’s *t* test. Data are shown as mean and SD. ***P < 0.001, ****P < 0.0001, ns, not significant. WT vs p.V233I, *P* = 0.2238; WT vs p.V466M, *P* = 0.0858; WT vs p.P44Lfs*5, *P* = 1.5 × 10^−7^; WT vs p.Q301E, *P *= 0.4089; WT vs p.R3C, *P* = 0.0004. (**C**) Immunoblotting of WT and mutant hCCDC174 bound to RNA in HEK293T cells. HuR was used as the internal control. (**D**) Quantitation of the oligo-dT–bound WT or mutant hCCDC174 normalized to HuR. *n* = 3 biological replicates. The statistics are analyzed by unpaired two-tailed Student’s *t* test. Data are shown as mean and SD. ***P < 0.001, ns, not significant. WT vs p.V233I, *P* = 0.3686; WT vs p.V466M, *P* = 0.1558; WT vs p.Q301E, *P* = 0.0005. (**E**) Immunoblotting results of WT and mutant hCCDC174 bound to human CDC5L (hCDC5L) in HEK293T cells. (**F**) Quantitation of hCDC5L-bound WT and mutant hCCDC174 normalized to hCDC5L. *n* = 3 biological replicates. The statistics are analyzed by unpaired two-tailed Student’s *t* test. Data are shown as mean and SD. *P < 0.05, **P < 0.01, ns, not significant. WT vs p.V233I, *P* = 0.0035; WT vs p.V466M, *P* = 0.0145; WT vs p.Q301E, *P* = 0.1074. Source data are available online for this figure.

Finally, we determined whether the patient-derived variants impair the normal function of CCDC174. We first performed immunoblot analysis in HEK293T cells transfected with HA-labeled WT or mutant human *CCDC174* (*hCCDC174*) expression plasmids. Compared with WT, the p.P44Lfs*5 and p.R3C variants significantly reduced the protein level of hCCDC174, whereas the other three missense variants p.V233I, p.V466M, and p.Q301E had no impact on protein level (Fig. 7A,B). Because our results indicated that hCCDC174 can bind to RNAs (Fig. EV1B) and that it interacts with the members of the PRP19/CDC5L complex (Fig. 6C). To further explore the functional impairment of the three missense variants (p.V233I, p.V466M, and p.Q301E), we examined the influence of these variants on the binding of hCCDC174 to RNAs or to human CDC5L (hCDC5L). The results showed that variant p.Q301E decreased the binding of hCCDC174 to RNAs (Fig. 7C,D), whereas variants p.V233I and p.V466M disrupted the interaction between hCCDC174 and hCDC5L (Fig. 7E,F). These findings suggested that all the patient-derived variants are functional impairment and pathogenic, thus establishing the causal effect of *CCDC174* variants on oocyte incompetence and female infertility.

## Discussion

Oocyte competence defect is one of causes for female infertility and recurrent IVF/ICSI failure, but the underlying genetic determinants remains largely unknown. Here, we found that biallelic variants in *CCDC174* cause oocyte competence defects and infertility, and deciphered that CCDC174 functions as a novel maternal-effect factor involved in AS regulation in oocytes. In vitro and in vivo studies further confirmed the crucial role of CCDC174 in oocyte development and female reproduction in humans and mice.

Genome-wide studies have estimated that 90–95% of human genes undergo AS (Pan et al, 2008; Wang et al, 2008). Defects in AS contribute to many diseases by affecting gene expression or by producing dysfunctional proteins isoforms (Nikom and Zheng, 2023). For instance, *RBM20* mutations induce abnormal AS of cardiac genes, resulting in dilated cardiomyopathy (Nishiyama et al, 2022). Mutations in the splicing factors TDP43, FUS, and members of the hnRNP family cause neurodegenerative diseases (Geuens et al, 2016; Nikom and Zheng, 2023). However, to date, no splicing related proteins have been reported to be associated with any human reproductive diseases. We utilized an in vivo model to confirm that CCDC174 exerts its functions in the AS of maternal mRNA and female reproduction through interactions with the PRP19/CDC5L complex. Importantly, genetic evidence and functional studies confirmed the pathogenicity of *CCDC174* variants, further establishing a causal relationship between CCDC174 deficiency and oocyte competence defects and female infertility. Our findings thus provide a novel genetic diagnostic marker for female infertility patients.

In mice and *Drosophila*, numerous mRNA splicing regulators have been shown to be essential for male fertility, including PRP19/CDC5L complex members, BCAS2 and PRP19 (Li et al, 2025). Our study clearly demonstrates that CCDC174 functions as a key mRNA splicing regulator and interacts with the PRP19/CDC5L complex. Moreover, *Ccdc174* is highly expressed in mouse testes. Together, these findings suggest that CCDC174 may play an essential role in male reproduction. Nevertheless, the specific function of CCDC174 in spermatogenesis and its potential involvement in male infertility remains completely unknown and requires further elucidation.

Previously, a homozygous stop-loss variant in *CCDC174* (c.1404 A > G, p.*468Trpext*6) has been reported to be associated with a syndrome of hypotonia and psychomotor developmental delay (Volodarsky et al, 2015). In our present study, the infertile women carrying *CCDC174* variants didn’t show death or neurodevelopmental disorders. It’s common that different variants in the same gene can cause different diseases such as *TRIP13* and *COX15* (Nussinov et al, 2022). The phenotypic differences may result from the varying degrees of disruption to protein function caused by the different types of variants (Zhang et al, 2020; Zhang et al, 2024). Overexpression of the variants associated with infertility did not result in cellular apoptosis as caused by p.*468Trpext*6 in the previous study (Appendix Fig. S5). So, the infertility-related variants we identified are less destructive to the protein compared to the previously reported stop-loss variants.

The PRP19/CDC5L complex is crucial for the splicing reaction by regulating the formation and progression of spliceosome conformations (van Maldegem et al, 2015). Oocyte-specific knockout of *Bcas2*, a member of this complex, results in aberrant AS of maternal mRNAs and infertility in female mice (Zhang et al, 2022b). In this study, CCDC174 interacts with core members of the PRP19/CDC5L complex and colocalizes with them in the nucleus. These results indicate that CCDC174 maintains the normal splicing of maternal mRNAs by binding to this complex and regulating its function. In addition, we found that the RNA and protein levels of many components of the PRP19/CDC5L complex are increased in CCDC174-deleted oocytes. One possible explanation for this is that CCDC174 deficiency leads to functional abnormalities of this complex and to disruptions in AS patterns. In response to AS defects, oocytes may activate the expression of this complex to maintain normal AS patterns. Our findings, together with the *Bcas2* knockout mouse model, indicate that the PRP19/CDC5L complex is required for the AS of maternal mRNAs and for successful female reproduction.

The acquisition of oocyte competence relies on the precise regulation and synchronous completion of nuclear and cytoplasmic maturation (Conti and Franciosi, 2018). In this study, we demonstrated that CCDC174 deficiency alters the splicing pattern of a gene network comprising 1,018 genes. GO analysis revealed that these genes are essential for two maturation processes: nuclear maturation (e.g., cell cycle regulation, DNA damage and repair, chromatin organization and remodeling) and cytoplasmic maturation (e.g., mRNA processing and cytoskeletal organization). Additionally, transcriptomic and proteomic analyses revealed aberrant homeostasis of stored mRNAs and proteins in *Ccdc174*^*OO−/−*^ oocytes, which also disrupts cytoplasmic maturation. Overall, dual abnormalities in nuclear and cytoplasmic maturation severely impair the developmental competence of oocytes, ultimately resulting in GV arrest and female infertility in mice.

According to the patient’s clinical records, patients with *CCDC174* variants exhibited no obvious phenotypes in other somatic tissues. This oocyte-restricted phenotype could be explained by several mechanisms. One plausible mechanism is that *Ccdc174* is highly expressed in oocytes but lowly in somatic tissues (Fig. 2), which likely renders oocytes more sensitive to *CCDC174* variants. In addition, genetic redundancy may contribute to this tissue-specific phenotype. In somatic cells, functionally redundant genes may compensate for impaired CCDC174 function, whereas such a compensatory mechanism is absent or insufficient in highly specialized oocytes. Furthermore, the unique process of oocyte meiosis requires strict temporal control over mRNA transcription and stability, which differs from the cell cycle and gene regulatory programs of somatic mitosis. These unique processes are likely more susceptible to dysfunction of CCDC174, leading to oocyte-specific phenotypes.

In this study, five patients with different *CCDC174* variants exhibited phenotypic heterogeneity. There are some possible explanations for phenotypic heterogeneity. Firstly, different types (e.g., impaired expression, CDC5L binding, or RNA binding) and degrees (severe vs. mild) of CCDC174 dysfunction caused by different variants might lead to phenotypic heterogeneity in these patients. Secondly, genetic modifiers may also play an important role in regulating the function of mutant CCDC174 and promoting phenotypic heterogeneity. Besides genetic factors, external factors such as lifestyle, environmental exposure, and assisted reproductive strategies may also influence the phenotypic characteristics of patients.

In addition, phenotypic differences exist between patients with *CCDC174* variants and *Ccdc174* knockout mice. Specifically, *Ccdc174*^*OO–/–*^ female mice exhibit a phenotype of oocyte maturation arrest, while infertility patients primarily display a phenotype of fertilization failure or early embryonic arrest. Compared to the complete loss of function resulting from gene knockout, the mutant CCDC174 retains partial biological activity that can support oocyte maturation but is insufficient for sustaining subsequent fertilization and embryonic development. Our functional studies further confirmed that the variants reduce the binding ability of CCDC174 with RNAs or with CDC5L rather than causing a complete loss of these functions. This further suggests that the phenotypic severity correlates with the degree of functional disruption in the protein.

This study has some limitations. First, we confirmed in mouse oocytes that CCDC174 deficiency leads to abnormal maternal mRNA splicing. However, due to the lack of oocyte samples from patients with *CCDC174* variants, we were unable to evaluate mRNA splicing in these oocytes. Second, in this study, we found that CCDC174 regulates the AS of maternal mRNAs by interacting with the PRP19/CDC5L spliceosomal complex. However, whether CCDC174 act as scaffold, regulate the activity of spliceosome, or have other unknown roles remains unclear, this deserves further investigation in future studies. Third, the compensatory upregulation of the PRP19/CDC5L complex components are not directly tested, which need further confirmation in future. Finally, our findings identify *CCDC174* as a novel pathogenic gene for oocyte competence defects. Nevertheless, treatment strategies for patients with CCDC174 variants remain to be explored. We attempted to rescue the oocyte maturation arrest phenotype by microinjecting wild-type *Ccdc174* mRNA into GV oocytes from knockout mice, but this strategy proved unsuccessful (Appendix Fig. S6). *Ccdc174* depletion severely disrupts the homeostasis of maternal mRNAs and proteins in oocytes. Affected molecules are involved in multiple key pathways governing oocyte development, such as GDF9 and BMP15 (essential factors regulating oocyte and follicle development). Disruption of these key factors and pathways cause irreversible damage to oocyte development. Therefore, reintroducing CCDC174 into GV oocytes cannot restore oocyte maturation. This limitation prevents a definitive functional rescue conclusion and reflects the temporal requirement of CCDC174 during oocyte development. Furthermore, CCDC174 may begin to function in earlier growing oocytes. Depletion of *Ccdc174* causes irreversible damage in early growing oocytes, therefore, reintroduction of wild-type CCDC174 into knockout fully-grown oocytes cannot rescue the phenotype, however, reintroduction of wild-type CCDC174 into early growing oocytes is technically challenging. Future studies could focus on targeting downstream effectors of CCDC174 or screening for small-molecule regulators of alternative splicing, which may provide possible treatment strategies to improve patient fertility.

**Figure 8:**
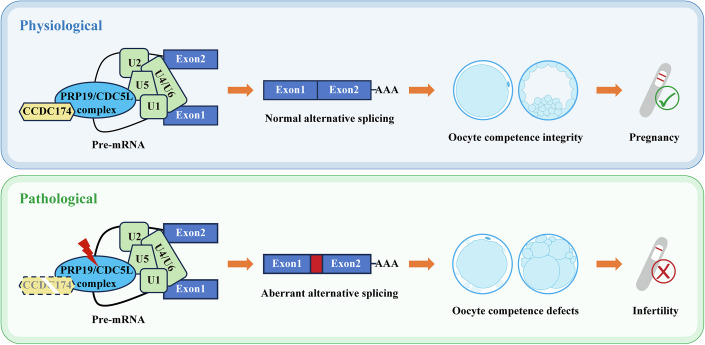
Schematic model of the physiological and pathological mechanisms of CCDC174 in oocyte development and female reproduction. CCDC174 regulates the AS of maternal mRNAs by interacting with the PRP19/CDC5L complex, which lays the foundation for oocyte competence and successful pregnancy. CCDC174 deficiency results in aberrant AS and oocyte competence defects, ultimately causing female infertility.

In conclusion, our study has elucidated the physiological and pathological roles that CCDC174 plays in oocyte development and female reproduction. CCDC174 regulates the AS of maternal mRNAs by interacting with the PRP19/CDC5L complex, and this process lays the foundation for oocyte competence and successful pregnancy. CCDC174 deficiency results in aberrant AS and oocyte competence defects, ultimately causing female infertility (Fig. 8). These findings expand our understanding of the regulatory mechanisms behind the AS of maternal mRNAs in oocytes and provide a novel genetic factor related to female infertility.

## Methods

**Table:** Reagents and tools table

Reagent/resource	Reference or source	Identifier or catalog number
**Experimental models**		
C57BL/6J (*M. musculus*)	Cyagen	N/A
**Recombinant DNA**		
pcmv6-mCcdc174-HA	This study	
pcmv6-mCdc5l-FLAG	This study	
pcmv6-mPrp19-FLAG	This study	
pcmv6-mBcas2-FLAG	This study	
pcmv6-mPlrg1-FLAG	This study	
pcmv6-hCDC5L-FLAG	This study	
Pcr3.1-HA-hCCDC174-WT	This study	
Pcr3.1-HA-hCCDC174-p.V233I	This study	
Pcr3.1-HA-hCCDC174-p.V466M	This study	
Pcr3.1-HA-hCCDC174-p.P44Lfs*5	This study	
Pcr3.1-HA-hCCDC174-p.Q301E	This study	
Pcr3.1-HA-hCCDC174-p.R3C	This study	
**Antibodies**		
Rabbit Anti-HA	Cell Signaling Technology	3724
Rabbit Anti-CCDC174	This study	
Rabbit Anti-Vinculin	Cell Signaling Technology	13901
Rabbit Anti-HuR	Cell Signaling Technology	12582
Rabbit Anti-GFP	Proteintech	50430-2-AP
Rabbit Anti-FLAG	Sigma-Aldrich	F7425
Rabbit Anti-CDC5L	Proteintech	Q99459
Rabbit Anti-PRP19	Abclonal	A12590
Rabbit Anti-BCAS2	Abclonal	A4398
Rabbit Anti-BMP15	Abclonal	A7321
Rabbit Anti-GDF9	Abclonal	A2739
Rabbit Control IgG	Abclonal	AC005
Alexa Fluor 594 donkey anti-mouse IgG	Thermo Fisher Scientific	A21203
Alexa Fluor 488 donkey anti-rabbit IgG	Thermo Fisher Scientific	A21206
**Oligonucleotides and other sequence-based reagents**		
PCR primers	This study	Appendix Table S1
**Chemicals, enzymes and other reagents**		
AgeI restriction enzyme	New England BioLabs	R3552S
M2 medium	Nanjing Luanchuang Co.	M01-B
2% paraformaldehyde	Sigma-Aldrich	P6148
Triton X-100	Sigma-Aldrich	T8787
bovine serum albumin	Sigma-Aldrich	B2064
Tween-20	Sigma-Aldrich	P9416
RNase inhibitor	New England BioLabs	M0314L
PolyJet In Vitro DNA Transfection Reagent	Signagen	100688
protease inhibitor cocktail	Bimake	B14001
Oligo d(T)_25_ magnetic beads	New England BioLabs	S1419S
EU	Thermo Fisher Scientific	E10345
Protein A/G Magnetic Beads	Selleck	B23202
**Software**		
GraphPad Prism	GraphPad Software	Version 10.2.2
ImageJ	https://imagej.nih.gov/ij/	N/A
Adobe Illustrator	Adobe	Version 2024
ZEISS Zen Lite	ZEISS	N/A
**Other**		
HiScribe T7 ARCA mRNA Kit	New England BioLabs	E2060S
RNeasy MinElute Cleanup Kit	Qiagen	74204
RNeasy Mini Kit	Qiagen	74104
PrimeScript RT Reagent Kit	Takara	RR047A
Amplification and Library Generation Kit	New England BioLabs	E6420
KOD-Plus Mutagenesis Kit	Toyobo Life Science	SMK-101
Click-iT Cell Reaction Buffer Kit	Thermo Fisher Scientific	C10269

### Clinical samples

A cohort of 3627 infertility patients with oocyte competence defects, including oocyte maturation arrest, fertilization failure, and embryonic arrest, were recruited from 22 collaborating hospitals and reproductive centers (Chen et al, 2025). In this study, five infertility patients who carried *CCDC174* variants were recruited from the Reproductive Medicine Center of the Shanghai Ninth Hospital affiliated with Shanghai Jiao Tong University and the Reproductive Medicine Center of the Shaanxi Maternal and Child Care Service Center. The inclusion criteria were as follows: (1) age younger than 40 years old; (2) primary infertility with unexplained etiology; (3) normal menstrual cycles, hormone levels, and chromosomes; (4) exclusion of male factors due to spermatogenic failure, hormonal disturbances, etc.; (5) IVF/ICSI failure due to oocyte maturation arrest, abnormal fertilization, or embryonic arrest, etc.; and (6) exclusion of other diseases that affect fertility such as ovarian dysfunction, fallopian tube lesions, endometriosis, endocrine disorders, etc. All blood samples were donated for the investigation after informed consent was obtained. The study was approved by the Ethics Committee of the Medical College of Fudan University and the Reproductive Study Ethics Committees of the respective hospitals. These experiments conformed to the principles set out in the WMA Declaration of Helsinki and the Department of Health and Human Services Belmont Report.

### Genetic studies

Genomic DNA was extracted from peripheral blood using the QIAamp DNA Blood Mini Kit (51104, Qiagen), and whole-exome sequencing (WES) was performed using a SeqCap EZ Exome Kit (Roche) on an Illumina NovaSeq 6000 platform (Illumina). Sequencing analysis was compared with the human reference sequence (NCBI Genome build GRCh37). Variants were annotated with GRCh37 and the dbSNP (version 138) database. Candidate variants were filtered according to the criteria described in a previous study (Wang et al, 2021). The *CCDC174* variants were verified by Sanger sequencing of the affected probands as well as all the available family members. The primers used are shown in Appendix Table S1.

### Generation of oocyte-specific knockout mice

The oocyte-specific knockout mice *Ccdc174*^*OO–/–*^ were prepared by Cyagen Biosciences Inc. All mouse strains were in a C57BL/6 J background. The targeting vector with loxP sites was co-injected with Cas9 and sgRNA to obtain *Ccdc17*^*fl/fl*^ mice. The loxP sites were located before Exon 2 and after Exon 3, and this deletion led to a loss-of-function frameshift mutation in *Ccdc174*. *Ccdc174*^*fl/fl*^ mice were crossed with *Zp3*-*Cre* mice to generate the oocyte-specific *Ccdc174* knockout mouse strain *Ccdc174*^*OO–/–*^. The genotypes of each generation of mice were confirmed by Sanger sequencing, and the primers are shown in Appendix Table S1. All mouse experiments were approved by the Medical Ethics Committee of the International Peace Maternity and Child Health Hospital of the China Welfare Institute (GKDW-A-2024-39).

### Oocyte collection and in vitro maturation

Ovaries were isolated from mice to obtain mouse GV oocytes. The GV oocytes were isolated from ovaries by puncturing the antral follicles with a fine needle on the stage of a dissecting microscope. For in vitro maturation, GV oocytes were cultured in M2 medium (M01-B, Nanjing Luanchuang Co.) under oil droplets at 37 °C in a 5% CO_2_ incubator for 12 h, and the proportion of oocytes at each stage was calculated.

### In vitro transcription and microinjection

The expression vectors were linearized by digestion with the AgeI restriction enzyme (R3552S, New England BioLabs) at 37 °C for 3 h. Purified linearized DNA was used as a template for the in vitro transcription of RNA, and this was followed by DNase I treatment and poly(A) polymerase tailing using the HiScribe T7 ARCA mRNA Kit (E2060S, New England BioLabs). Finally, the RNAs were purified and dissolved in nuclease-free water using the RNeasy MinElute Cleanup Kit (74204, Qiagen). GV oocytes were microinjected with RNAs (500 ng/μL) on the stage of an inverted microscope (Leica) with micromanipulators (Eppendorf). After injection, the oocytes were individually transferred to M2 medium for culturing, and oocytes at different developmental stages were obtained for further analyses.

### Immunofluorescence

The oocytes were fixed in 2% paraformaldehyde (P6148, Sigma-Aldrich) in phosphate-buffered saline (PBS) for 30 min at room temperature and then permeabilized in PBS containing 0.5% Triton X-100 (T8787, Sigma-Aldrich) for 20 min at room temperature. After incubation for 1 h in a blocking buffer containing 3% bovine serum albumin (B2064, Sigma-Aldrich), 0.1% Tween-20 (P9416, Sigma-Aldrich), and 0.01% Triton X-100 (T8787, Sigma-Aldrich) in PBS at room temperature, oocytes were incubated with primary antibodies diluted in blocking buffer for 1 h at 37 °C. After three washes, oocytes were incubated with secondary antibodies for 1 h at 37 °C. Oocytes were briefly stained with Hoechst for 5 min at room temperature. The samples were resuspended in PBS and imaged using a laser scanning confocal microscope (LSM880, Zeiss). The antibodies used for immunofluorescence are listed in Appendix Table S2.

### Quantitative real-time PCR (qRT-PCR)

Total RNA was extracted with a RNeasy Mini Kit (74104, Qiagen), and reverse transcription was performed with the PrimeScript RT Reagent Kit (RR047A, Takara) according to the manufacturer’s instructions. qRT-PCR was performed with TB Green Premix Ex Taq (Takara) in triplicate on a LightCycler 480 II System (Roche). The expression of *Ccdc174* was normalized by comparison to the expression of an internal mouse *Actin* control. The qRT-PCR primers for *Ccdc174* and *Actin* are shown in Appendix Table S1.

### Immunoblot analysis

All immunoblot samples, including oocytes, embryos, or cell lines, were heated at 100 °C for 10 min. Equal amounts of proteins were separated by SDS-polyacrylamide gel electrophoresis (EZBiolab) and then transferred to nitrocellulose membranes (Pall Corporation). The membranes were blocked with 5% non-fat milk diluted in PBS with 0.1% Tween-20 (PBST) for 1 h and then incubated with primary antibodies at 4 °C overnight. The membranes were washed with PBST three times and incubated with secondary antibodies for 1 h at room temperature followed by three washes with PBST. Finally, enhanced chemiluminescence imaging was performed on a chemiluminescent imaging system (5200, Tanon). The immunoblots were quantified with the ImageJ software (National Institutes of Health). The antibodies used in the immunoblot analysis are listed in Appendix Table S2.

### RNA sequencing (RNA-seq)

Each sample included ten oocytes, and RNA-seq libraries were prepared using a cDNA Synthesis, Amplification and Library Generation Kit (E6420, NEB). RNA quality was examined by gel electrophoresis and with Qubit (Thermo Fisher Scientific). RNA samples were sequenced on an Illumina Novaseq 6000 instrument through the commercial service provided by Genergy Biotechnology Co. Ltd. The raw data were processed by Skewer, and data quality was checked using FastQC v0.11.2. The read length was 2  × 150 bp. Clean reads were aligned to the mouse genome mm10 using STAR and StringTie. The expression levels of the transcripts were calculated by fragments per kilobase million (FPKM), and differential gene expression analysis was performed using DESeq2 with read counts data. The RNA-seq results are listed in Dataset EV1.

### Proteomics

Each sample included 20 oocytes, and an iST Sample Preparation kit (PreOmics) was used for sample preparation according to the manufacturer’s protocols. All samples were analyzed by LC-MS/MS using a nanoElute 2 liquid chromatography system connected to an ion-mobility spectrometry quadrupole time-of-flight mass spectrometer (Bruker Daltonik). The Spectronaut 18 software (Biognosys AG) was used for searching the raw files against the UniProt mouse proteome database (21,984 entries). For the database search, trypsin was used as the digestion enzyme with specific cleavage. Carbamidomethyl on cysteine was specified as the fixed modification, and oxidation on methionine was specified as the variable modification. The retention time prediction type was set to dynamic iRT, and data extraction was determined by Spectronaut based on extensive mass calibration data. Spectronaut can determine the ideal extraction window based on the iRT calibration and gradient stability. The FDR cutoff on both the precursor level and the protein level was 1%. Decoy generation was set to mutated, which is similar to scrambled but only applies a random number of amino acid position swaps (min = 2, max = length/2). The normalization strategy was set to local normalization. Peptides that passed the 1% FDR cutoff were used to calculate the major group quantities using the MaxLFQ method. The proteomics results are listed in Dataset EV2.

### Fluorescence in situ hybridization (FISH)

Oocytes were fixed with 2% paraformaldehyde in RNase-free PBS for 30 min at room temperature and permeabilized in 0.5% Triton X-100 in PBS for 20 min with RNase inhibitor (M0314L, New England BioLabs). The oocytes were then incubated with 100 µl hybridization buffer containing 100 nM hybridization probes overnight at 37 °C. After hybridization, the oocytes were washed three times with 2× SSC and labeled with Hoechst 33342 for 5 min. The oocytes were imaged using a Zeiss LSM880 confocal microscope.

### Construction of expression vectors and transfections

The full-length gene coding sequences were amplified and inserted into expression vectors containing different tags. For the construction of mutant CCDC174 expression vectors, site-directed mutagenesis was performed to introduce the identified variants into the wild-type (WT) vector according to the instructions of the KOD-Plus Mutagenesis Kit (SMK-101, Toyobo Life Science). HEK293T cells (Cell Bank of the Shanghai Institute for Biological Sciences) were cultured in DMEM (Gibco) supplemented with 10% fetal bovine serum (Gibco) and 1% penicillin/streptomycin (Gibco) in a 5% CO_2_ atmosphere at 37 °C. The WT and mutant expression vectors were transfected into HEK293T cells using the PolyJet In Vitro DNA Transfection Reagent (100688, Signagen) according to a standard protocol.

### Oligo(dT) pull-down assay

HEK293T cells were harvested 36 h post-transfection and washed three times with cold PBS. Cells were lysed in NP-40 lysis buffer (50 mM Tris, 150 mM NaCl, 0.5% NP-40, pH 7.5) with 1% protease inhibitor cocktail (B14001, Bimake) and RNase inhibitor. Equal amounts of protein extracts were incubated with Oligo d(T)_25_ magnetic beads (S1419S, New England BioLabs). After incubation at 4 °C for 2 h, the beads were washed with lysis buffer three times. The bead-bound proteins were then eluted using SDS sample buffer.

### 5-ethynyluridine (EU) incorporation assay

Oocytes were cultured in M2 medium containing 1 mM EU (E10345, Thermo Fisher Scientific) for 1 h at 37 °C. The oocytes were then fixed with 2% paraformaldehyde for 30 min and permeabilized in PBS containing 0.5% Triton X-100 for 30 min. Finally, the oocytes were labeled with Alexa Fluor 594 Azide (A10270, Thermo Fisher Scientific) using a Click-iT Cell Reaction Buffer Kit (C10269, Thermo Fisher Scientific). The oocytes were imaged using a Zeiss LSM880 confocal microscope.

### AS analysis

rMATS was used to analyze the AS events in the WT and *Ccdc174*^*OO–/–*^ groups. AS events with a |Percent Spliced In value (PSI)| >0.1 and a *P* value < 0.05 were categorized as differential AS events, and these were classified into five types: skipped exons, mutually exclusive exons, retained introns, alternative 3′ splice sites, and alternative 5′ splice sites. The results of the AS analysis are listed in Dataset EV3. The RT-PCR products were quantified using ImageJ software. Splicing ratios are represented as PSI values, which represent the percentage of a gene’s mRNA transcripts that include a specific exon or splice site. The RT-PCR primers used to amplify different isoforms are shown in Appendix Table S1.

### Immunoprecipitation (IP) and Co-IP

Mouse ovaries were washed twice with PBS and lysed in NP-40 lysis buffer with 1% protease inhibitor cocktail. A CCDC174 antibody was added to the lysate and was incubated overnight at 4 °C. The lysates were then incubated with Protein A/G Magnetic Beads (B23202, Selleck) for 2 h at 4 °C, and after extensive washing three times with IP buffer the bound proteins were eluted with 2× SDS sample buffer. The samples were separated by SDS-polyacrylamide gel electrophoresis, and the peptides were excised from the gels and identified by LC-MS/MS analysis. For the Co-IP assay, GFP-CCDC174 plasmids were co-transfected with the HA-CDC5L, HA-PRP19, and HA-PLRG1 plasmids into HEK293T cells. At 36 h after transfection, total cell proteins were extracted with NP-40 lysis buffer containing 1% protease inhibitor cocktail. Total protein was incubated with anti-HA beads (B26202, Selleck) at 4 °C for 3 h. The beads were then washed three times with lysis buffer and then boiled with SDS sample buffer for immunoblot analysis.

### Statistical analysis

Statistical analysis was performed with GraphPad Prism (GraphPad Software). The results are given as the means and standard deviations (SDs). Each experiment included at least three independent samples and was repeated at least three times. Group comparisons were made by two-tailed unpaired Student’s *t* tests. Statistically significant values of *P* < 0.05, *P* < 0.01, *P* < 0.001, and *P* < 0.0001 are indicated by (*), (**), (***), and (****), respectively.

## Supplementary information

Appendix

Peer Review File

Dataset EV1

Dataset EV2

Dataset EV3

Dataset EV4

Source data Fig. 2

Source data Fig. 3

Source data Fig. 5

Source data Fig. 6

Source data Fig. 7

Expanded View Figures

## Data Availability

The raw RNA-seq data reported in this paper have been deposited in the Genome Sequence Archive in National Genomics Data Center, China National Center for Bioinformation/Beijing Institute of Genomics, Chinese Academy of Sciences (GSA: CRA029850). The raw proteomic data reported in this paper have been deposited in the OMIX, China National Center for Bioinformation/Beijing Institute of Genomics, Chinese Academy of Science. Oocyte proteome data (OMIX011823) are publicly accessible at https://ngdc.cncb.ac.cn/omix/release/OMIX011823. IP-MS data (OMIX011822) are publicly accessible at https://ngdc.cncb.ac.cn/omix/release/OMIX011822. The source data of this paper are collected in the following database record: biostudies:S-SCDT-10_1038-S44321-026-00448-y.
