# Supplementary material for: CCDC174 deficiency impaired human fertility by affecting the alternative splicing of maternal mRNAs
Source: EMBO Mol Med. 2026 May 12;18(6):2436–54. doi: 10.1038/s44321-026-00448-y (PMC13270137; doi:10.1038/s44321-026-00448-y)
Supplement: Supplementary file 1 — Appendix [file 44321_2026_448_MOESM1_ESM.pdf]

Appendix

CCDC174 deficiency impaired human fertility by affecting the alternative splicing of maternal mRNAs

Weijie Wang[1,†,*], Zhiqi Pan[2,†], Huixia Jing[2,†], Rong Shi[3,†], Ling Wu[4,†], Jinjie Wang[2], Biaobang Chen[2], Jian Mu[2], Zhihua Zhang[2], Tianyu Wu[2], Qiaoli Li[2], Juanzi Shi[3], Yanping Kuang[4], Lin He[5], and Lei Wang[2,6,*], Qing Sang[2,*]

Table of contents

Appendix Fig. S1 2

Appendix Fig. S2 3

Appendix Fig. S3 4

Appendix Fig. S4 5

Appendix Fig. S5 6

Appendix Fig. S6 7

Appendix Table S1 8

Appendix Table S2 10

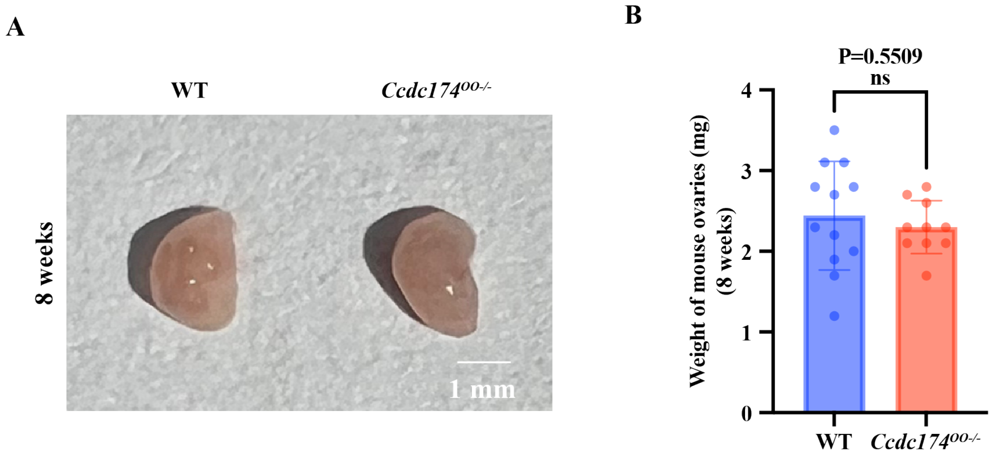

Appendix Fig. S1. The ovary weight of WT and *Ccdc174*$^{OO–/–}$ mice. (A) Representative images of ovaries from 8-week-old mice. Scale bar = 1 mm. (B) The ovary weight of 8-week-old WT and *Ccdc174*$^{OO–/–}$ mice. ns, not significant.

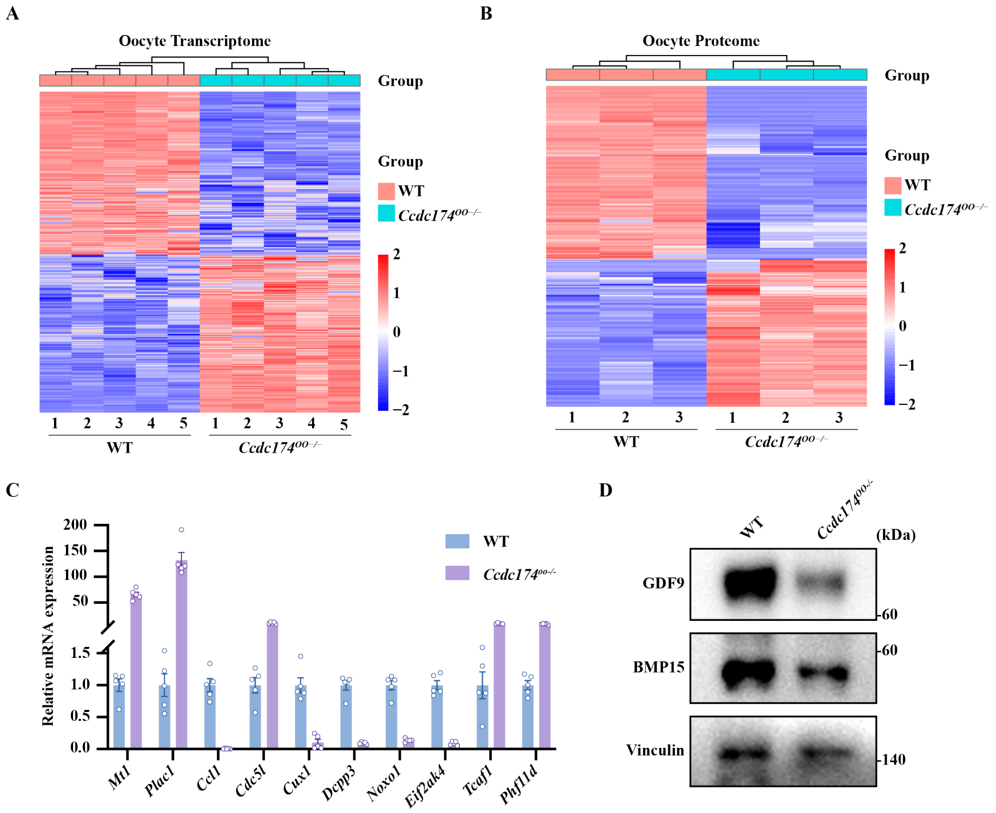

Appendix Fig. S2. Analysis and validation of the transcriptome and proteome of oocytes. (A, B) Heatmap showing relative expression levels of genes (A) and proteins (B) in 3-week-old WT and *Ccdc174*$^{OO-/-}$ oocytes. (C, D) qRT–PCR and immunoblotting results verified the expression of differentially expressed genes (C) and proteins (D) in the transcriptome and proteome analysis.

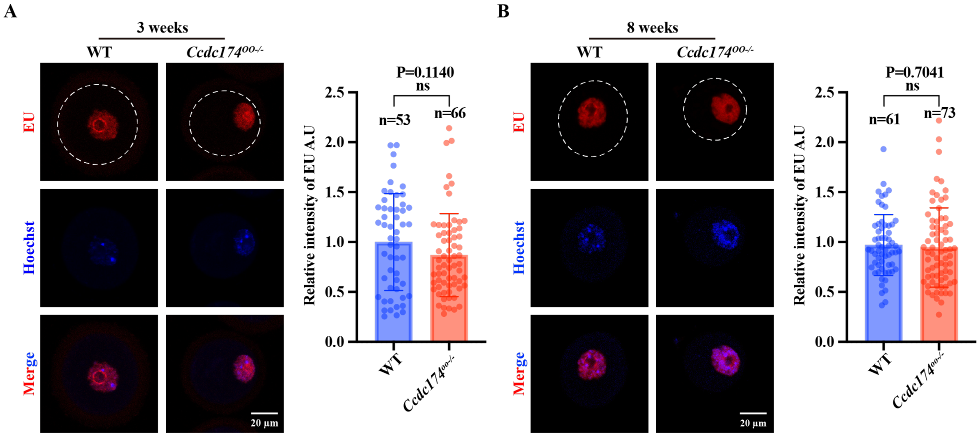

Appendix Fig. S3. Evaluation of transcriptional activity in WT and *Ccdc174*$^{OO–/–}$ oocytes. (A) Representative images and statistical graphs of EU staining for WT and *Ccdc174*$^{OO–/–}$ oocytes from 3-week-old mice. (B) Representative images and statistical graphs of EU staining for WT and *Ccdc174*$^{OO–/–}$ oocytes from 8-week-old mice. ns, not significant. The number of analyzed oocytes is indicated (n). Scale bars = 20 µm.

A

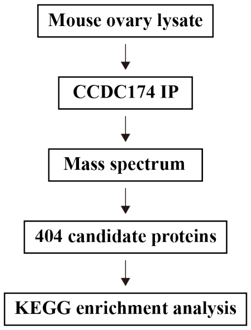

B

Complex	Protein name (Gene name)
PRP19/CDC5L complex components	Cell division cycle 5-like protein (Cdc5l)
	Pre-mRNA-processing factor 19 (Prpf19)
	Pleiotropic regulator 1 (Plrg1)
	Pre-mRNA-splicing factor SPF27 (Bcas2)
PRP19/CDC5L-related proteins	Pre-mRNA-splicing factor SYF1 (Xab2)
	Crooked neck-like protein 1 (Crnkl1)
	RNA helicase aquarius (Aqr)
	Serine/arginine-rich splicing factor 7 (Srsf7)
	Peptidyl-prolyl cis-trans isomerase-like 1(Ppil1)

Appendix Fig. S4. IP-MS for the identification of interacting proteins with CCDC174.

(A) Strategy and flowchart for the identification of CCDC174-interacting proteins. (B) The PRP19/CDC5L complex members identified by IP-MS that interact with CCDC174.

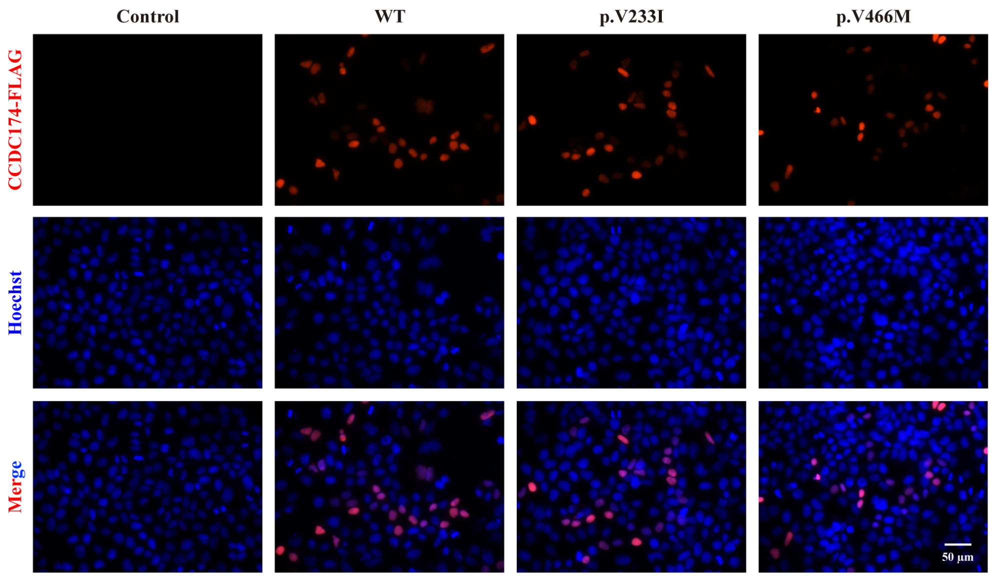

Appendix Fig. S5. Overexpression of mutant CCDC174 does not induce rapid and massive cell apoptosis. HeLa cells were transfected with FLAG-tagged full-length WT or mutant *CCDC174* expression plasmids and stained with an anti-FLAG antibody (Red). Nuclei were labeled with Hoechst (blue). Scale bars = 50 μm.

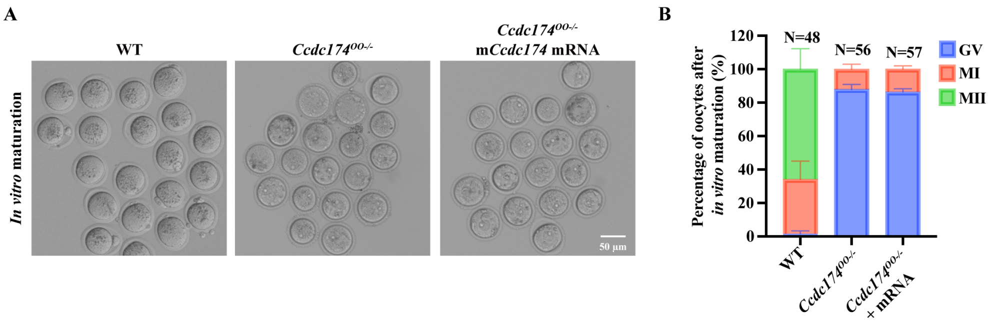

Appendix Fig. S6. Microinjection of mouse *Ccdc174* mRNA into *Ccdc174*$^{OO-/-}$ oocytes could not rescue oocyte maturation. (A) Representative images of oocytes after in vitro maturation (IVM). Scale bar = 50 μm. (B) The proportion of oocytes at different stages after IVM. The number of analyzed oocytes is indicated (N). *n* = 3 biological replicates.

Appendix Table S1. Primer sequences.

Primer name	Primer sequence (5' to 3')	Application
Ccdc174-F1	CTGACCTTTAACTTCAGCCTCG	Identification of Mouse genotypes
Ccdc174-R1	ATTCTCCAAGGGAGATTTGAGCTT	
Ccdc174-R2	TCCAGAGTCCTATCTCCAGATTCAA	
Ccdc174-qPCR-F	GAACAGCAAGGTGGAGGTCATC	qRT-PCR
Ccdc174-qPCR-R	AGCTCGGAGTTCTGACTGCTTC	
Mt1-qPCR-F	AGATCTCGGAATGGACCCCA	
Mt1-qPCR-R	AGGAGCAGCAGCTCTTCTTG	
Plac1-qPCR-F	CATCCGCATCAAGGCTGTCTCT	
Plac1-qPCR-R	TTGCTAGGTGCTTTCGCGGAGT	
Ccl1-qPCR-F	GCTTACGGTCTCCAATAGCTGC	
Ccl1-qPCR-R	GCTTTCTCTACCTTTGTTCAGCC	
Cdc5l-qPCR-F	ACAGCATCGCTCTGAGAACACC	
Cdc5l-qPCR-R	CCCACCTTTCAATGGAGTGTCC	
Cux1-qPCR-F	CCAAGAACGGCATTTGCCAGAG	
Cux1-qPCR-R	CCTTTCTGGGTCAGTTTGCTCC	
Dcpp3-qPCR-F	GGTCCAGAAGTTGGAAAACATTCC	
Dcpp3-qPCR-R	GAGCCATACACTTGACCGTCCT	
Noxo1-qPCR-F	TCAGCAGGTAGCCTGGTTTCCA	
Noxo1-qPCR-R	CACGGATAGCTCATCAGAGCGA	
Eif2ak4-qPCR-F	CGATGGTGACTGCCTCAGAAAG	
Eif2ak4-qPCR-R	GGTTCAACAGCCAGGAGATGAC	
Tcaf1-qPCR-F	CCACTCTGGAAGTGACCTCTCA	
Tcaf1-qPCR-R	CCTTCCAGGTATGTAGAGTCCG	
Phf11d-qPCR-F	GACAGAAGAGCCTCATGGTCAC	

Phf11d-qPCR-R	TCTGCAAGTGCCTCAAGCTGCT	
Actin-qPCR-F	CATTGCTGACAGGATGCAGAAGG	
Actin-qPCR-R	TGCTGGAAGGTGGACAGTGAGG	
Ddx47-AS-F	AGCTTGTGACCAGTTGGGATGG	AS analysis
Ddx47-AS-R	AGTCGGCCAGGAGTAGCTATTAC	
Nobox-AS-F	CACCTATTCGAAGGGCCAACCT	
Nobox-AS-R	GGTTAGGAAAGTAACCCTCTGC	
Cdc7-AS-F	TCAGCAGTGTACCCCAAAAACT	
Cdc7-AS-R	GTCTGGTCAGGACACTTTGTCA	
Pabpc1l-AS-F	CAGTTCTGGCTCCATAACTCC	
Pabpc1l-AS-R	CATGCCCGTGATCTTGC	
Clk1-AS-F	GGAGTGGAAGAAGCAGTTACAA	
Clk1-AS-R	CTCCCACTTTATGATCGATGCA	
Nrf1-AS-F	CAGCTGATGAGGTAACTGCCCA	
Nrf1-AS-R	CACAGCAGCAGCAGCGGCCATT	
CCDC174-V233I-F	GCCTCTGTAGTTCCCTGTTT	Identification of variants in infertile patients
CCDC174-V233I-R	TCCACTTCTCTCAGGGAACA	
CCDC174-V466M-F	ACATACGTCACCCACTCCTG	
CCDC174-V466M-R	TGGAAGCTGAACCCTTGGAA	
CCDC174-P44Lfs*5-F	TCTTAAGGCTGAACTCTTCCGA	
CCDC174-P44Lfs*5-R	ACACCCTGATATTTTCCGCTAT	
CCDC174-Q301E-F	AGGATCTCAAGGACAGCTTCT	
CCDC174-Q301E-R	TGTCATTCTGCATCACTTTGGT	
CCDC174-R3C-F	CCTCCGCACTCTATGACCTT	
CCDC174-R3C-R	GCGCATGTCAGAGGAGAGTA	

Appendix Table S2. Antibody information.

Antibody name	Manufacture (Catalogue number)	Applications (Dilution ratio)
Anti-HA	Cell Signaling Technology (3724)	WB/IF (1:1000/1:200)
Anti-CCDC174	Synthesized by Abclonal	WB/IP (1:1000/5μg/mL)
Anti-Vinculin	Cell Signaling Technology (13901)	WB (1:1000)
Anti-HuR	Cell Signaling Technology (12582)	WB (1:1000)
Anti-GFP	Proteintech (50430-2-AP)	WB (1:1000)
Anti-FLAG	Sigma-Aldrich (F7425)	WB/IF (1:1000/1:200)
Anti-CDC5L	Proteintech (Q99459)	WB (1:1000)
Anti-PRP19	Abclonal (A12590)	WB (1:1000)
Anti-BCAS2	Abclonal (A4398)	WB (1:1000)
Anti-BMP15	Abclonal (A7321)	WB (1:1000)
Anti-GDF9	Abclonal (A2739)	WB (1:1000)
Rabbit Control IgG	Abclonal (AC005)	IP (5μg/mL)
Hoechst	BD Biosciences (33342)	IF (1:500)
Alexa Fluor 594 donkey anti-mouse IgG	Thermo Fisher Scientific (A21203)	IF (1:500)
Alexa Fluor 488 donkey anti-rabbit IgG	Thermo Fisher Scientific (A21206)	IF (1:500)
