# Supplementary figures and images for: CCDC174 deficiency impaired human fertility by affecting the alternative splicing of maternal mRNAs
Source: EMBO Mol Med. 2026 May 12;18(6):2436–54. doi: 10.1038/s44321-026-00448-y (PMC13270137; doi:10.1038/s44321-026-00448-y)

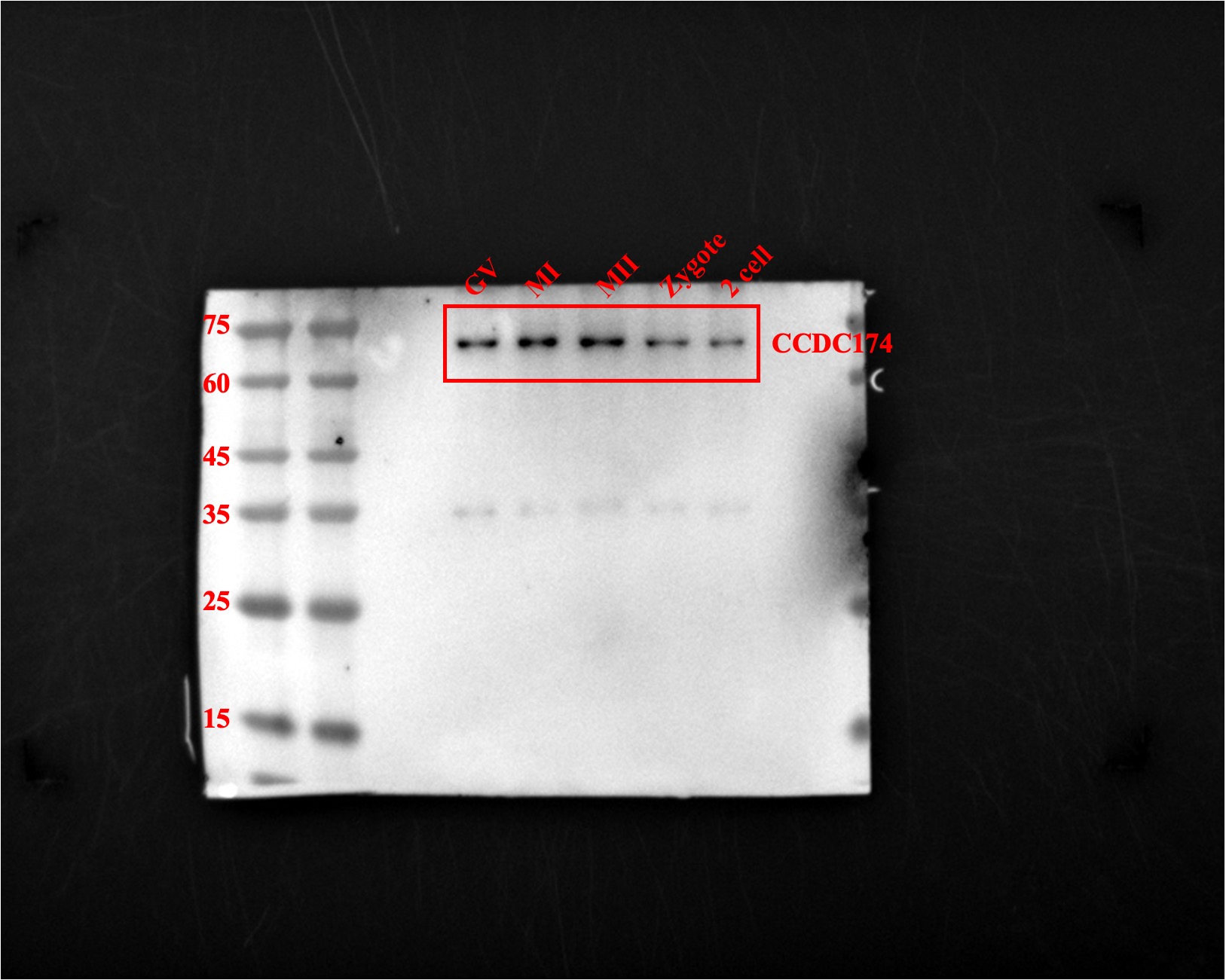

Supplement: Supplementary file 7 — Source data Fig. 2 [file 44321_2026_448_MOESM7_ESM.zip › Figure 2/2B/CCDC174.jpg]

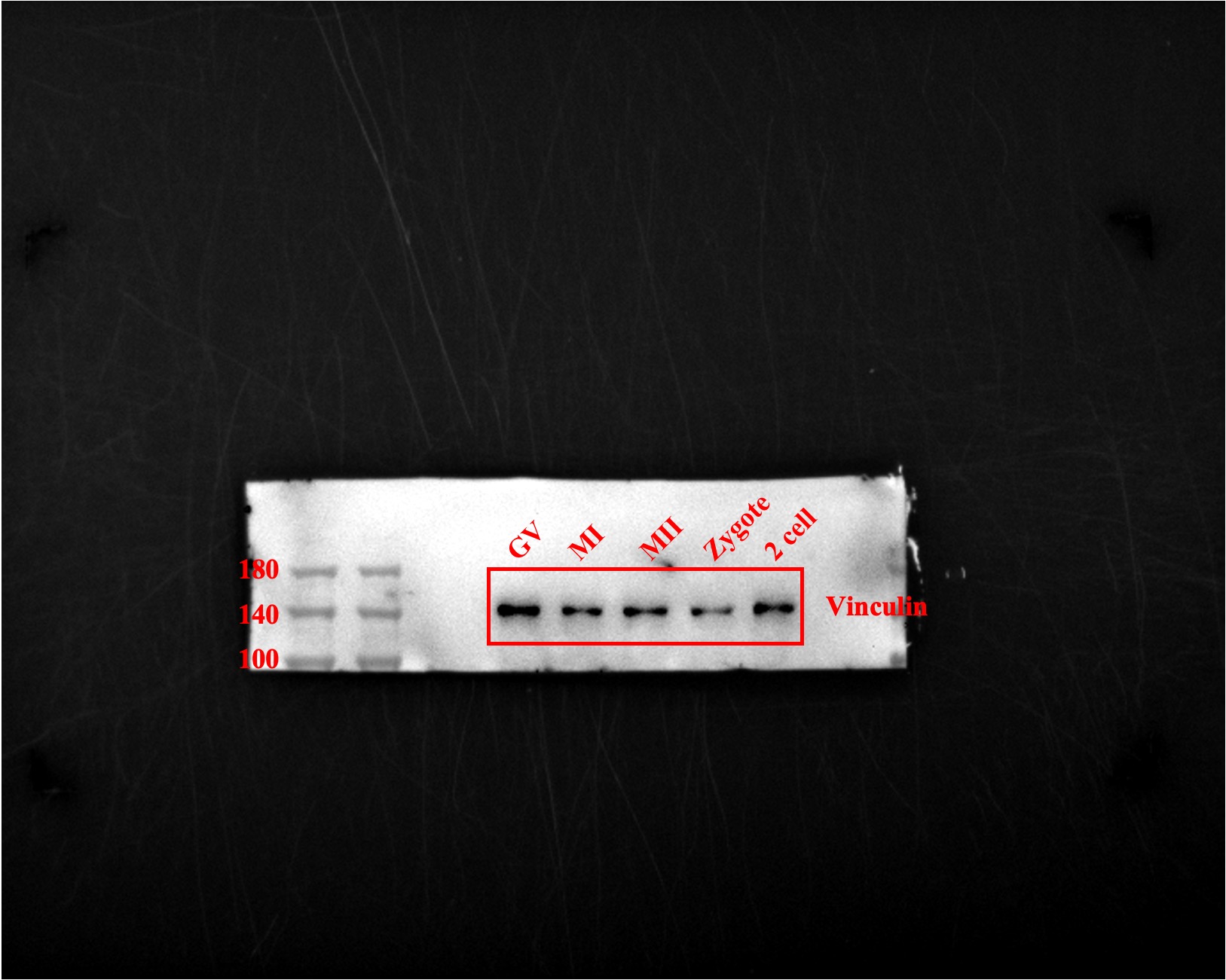

Supplement: Supplementary file 7 — Source data Fig. 2 [file 44321_2026_448_MOESM7_ESM.zip › Figure 2/2B/Vinculin.jpg]

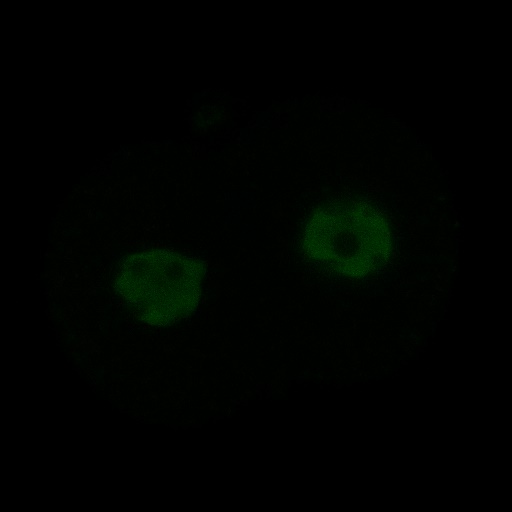

Supplement: Supplementary file 7 — Source data Fig. 2 [file 44321_2026_448_MOESM7_ESM.zip › Figure 2/2D/2cell/CCDC174-HA.jpg]

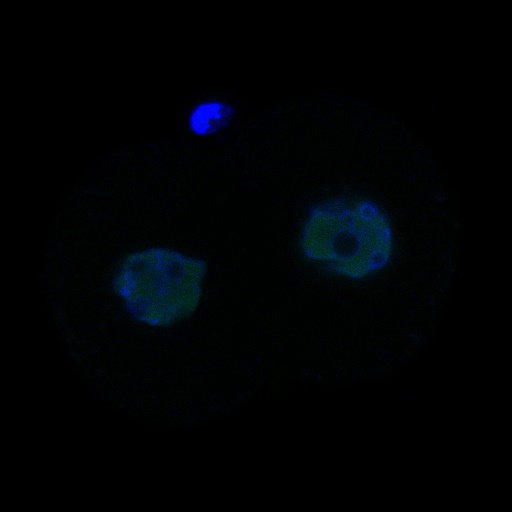

Supplement: Supplementary file 7 — Source data Fig. 2 [file 44321_2026_448_MOESM7_ESM.zip › Figure 2/2D/2cell/Merge.jpg]

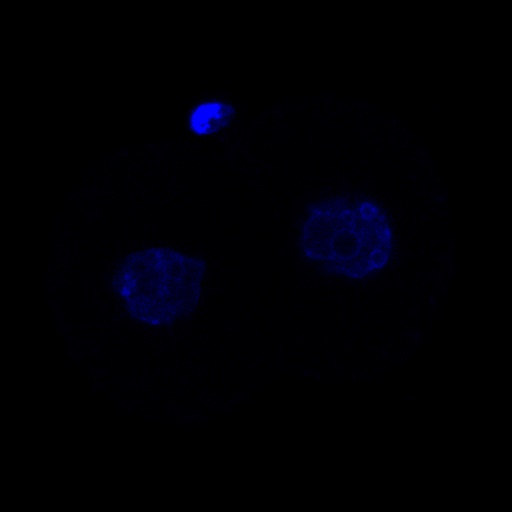

Supplement: Supplementary file 7 — Source data Fig. 2 [file 44321_2026_448_MOESM7_ESM.zip › Figure 2/2D/2cell/Hoechst.jpg]

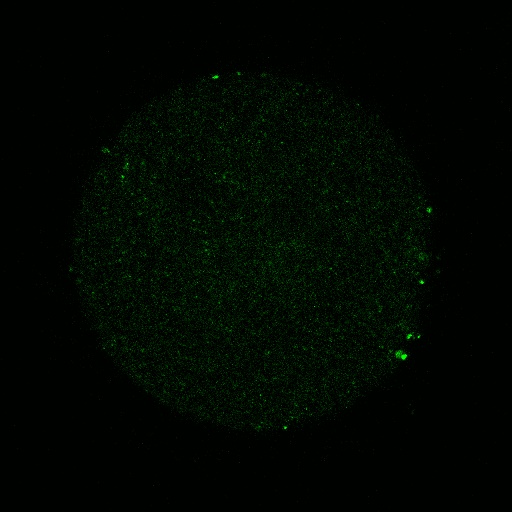

Supplement: Supplementary file 7 — Source data Fig. 2 [file 44321_2026_448_MOESM7_ESM.zip › Figure 2/2D/MI/CCDC174-HA.jpg]

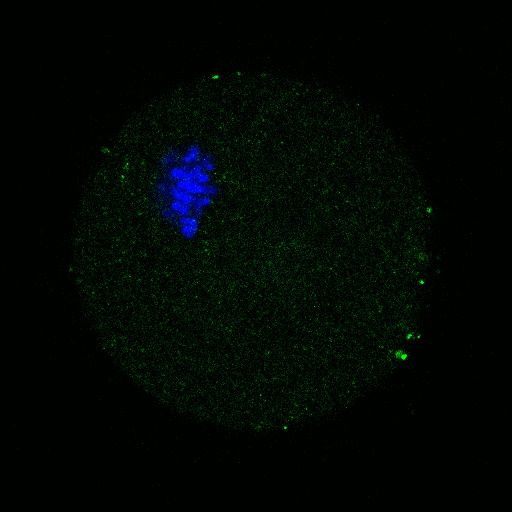

Supplement: Supplementary file 7 — Source data Fig. 2 [file 44321_2026_448_MOESM7_ESM.zip › Figure 2/2D/MI/Merge.jpg]

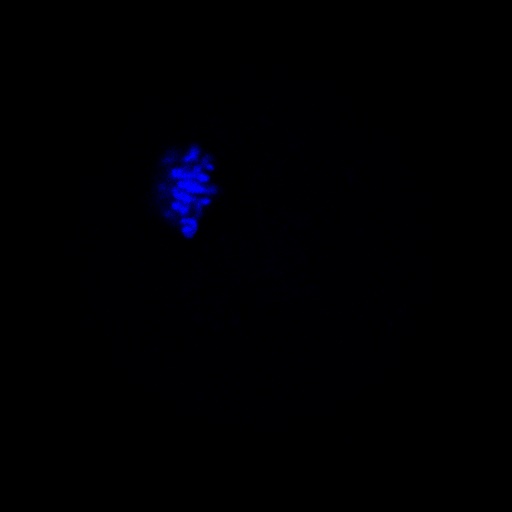

Supplement: Supplementary file 7 — Source data Fig. 2 [file 44321_2026_448_MOESM7_ESM.zip › Figure 2/2D/MI/Hoechst.jpg]

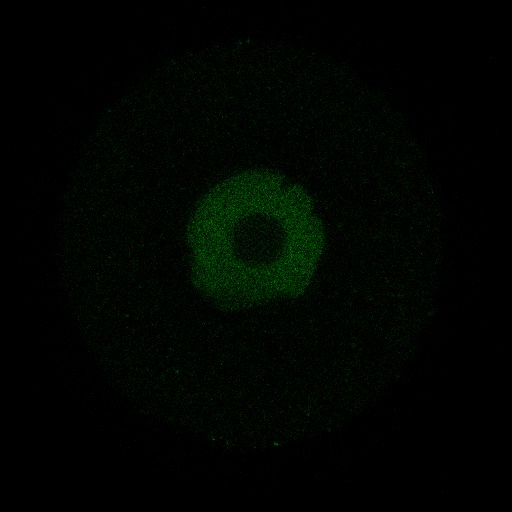

Supplement: Supplementary file 7 — Source data Fig. 2 [file 44321_2026_448_MOESM7_ESM.zip › Figure 2/2D/GV/CCDC174-HA.jpg]

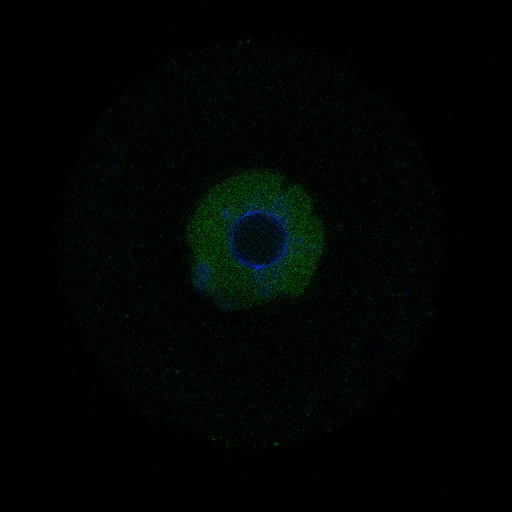

Supplement: Supplementary file 7 — Source data Fig. 2 [file 44321_2026_448_MOESM7_ESM.zip › Figure 2/2D/GV/Merge.jpg]

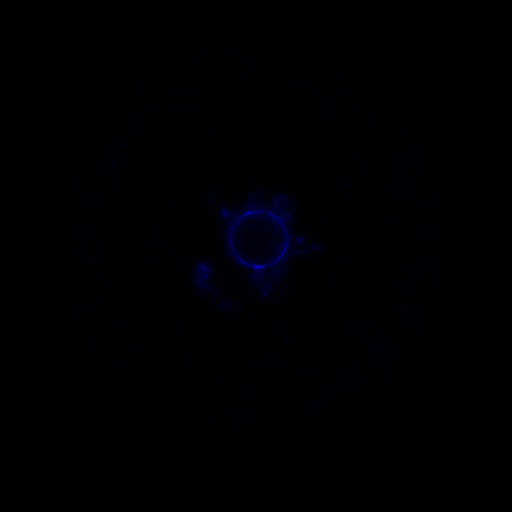

Supplement: Supplementary file 7 — Source data Fig. 2 [file 44321_2026_448_MOESM7_ESM.zip › Figure 2/2D/GV/Hoechst.jpg]

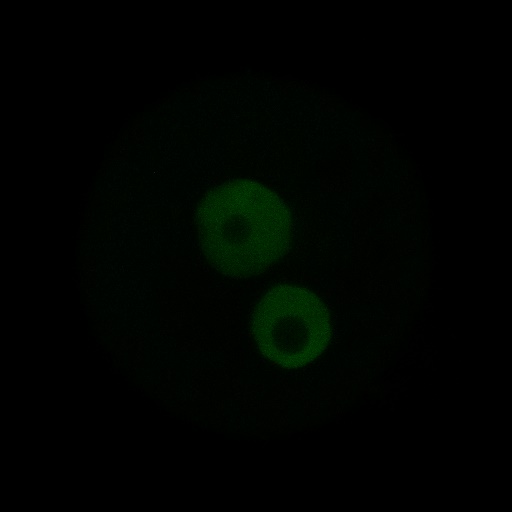

Supplement: Supplementary file 7 — Source data Fig. 2 [file 44321_2026_448_MOESM7_ESM.zip › Figure 2/2D/2pn/CCDC174-HA.jpg]

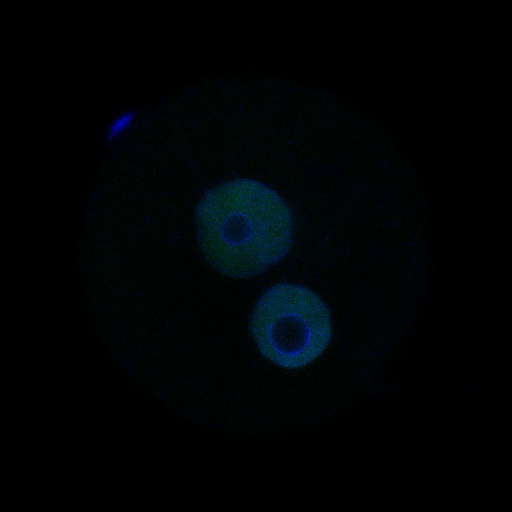

Supplement: Supplementary file 7 — Source data Fig. 2 [file 44321_2026_448_MOESM7_ESM.zip › Figure 2/2D/2pn/Merge.jpg]

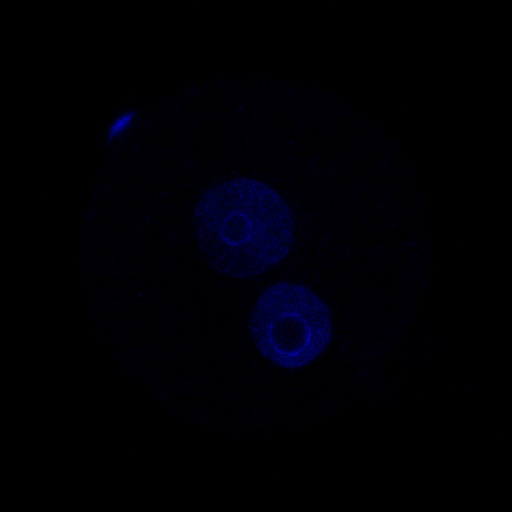

Supplement: Supplementary file 7 — Source data Fig. 2 [file 44321_2026_448_MOESM7_ESM.zip › Figure 2/2D/2pn/Hoechst.jpg]

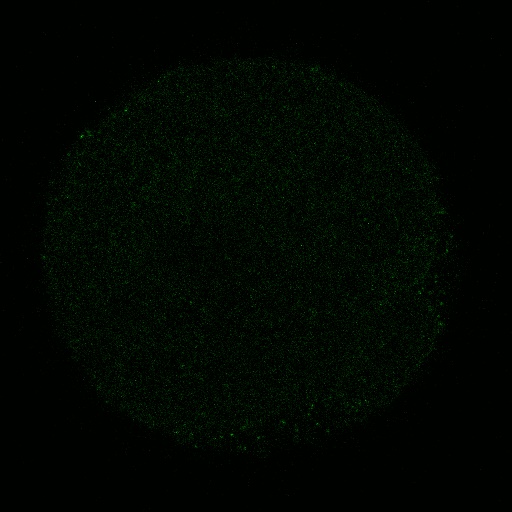

Supplement: Supplementary file 7 — Source data Fig. 2 [file 44321_2026_448_MOESM7_ESM.zip › Figure 2/2D/MII/CCDC174-HA.jpg]

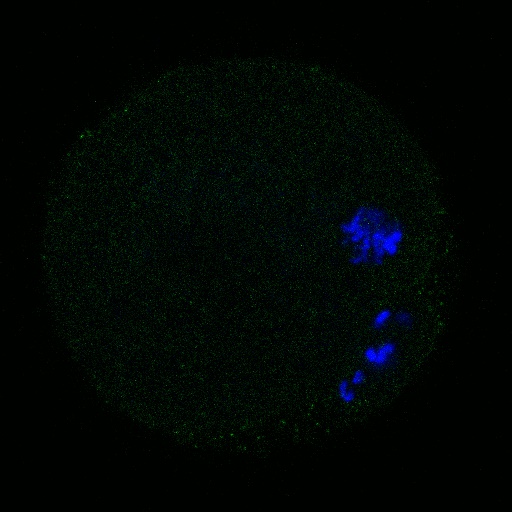

Supplement: Supplementary file 7 — Source data Fig. 2 [file 44321_2026_448_MOESM7_ESM.zip › Figure 2/2D/MII/Merge.jpg]

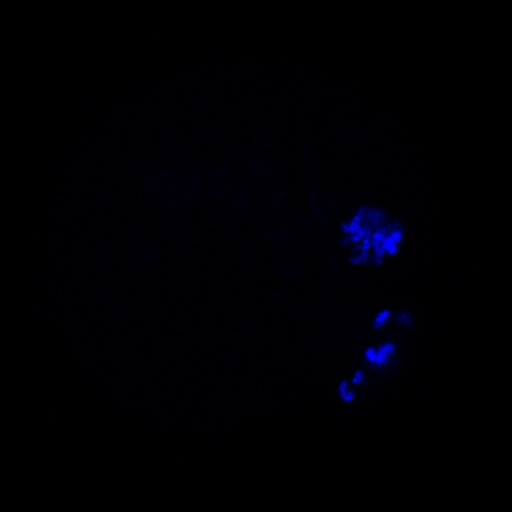

Supplement: Supplementary file 7 — Source data Fig. 2 [file 44321_2026_448_MOESM7_ESM.zip › Figure 2/2D/MII/Hoechst.jpg]

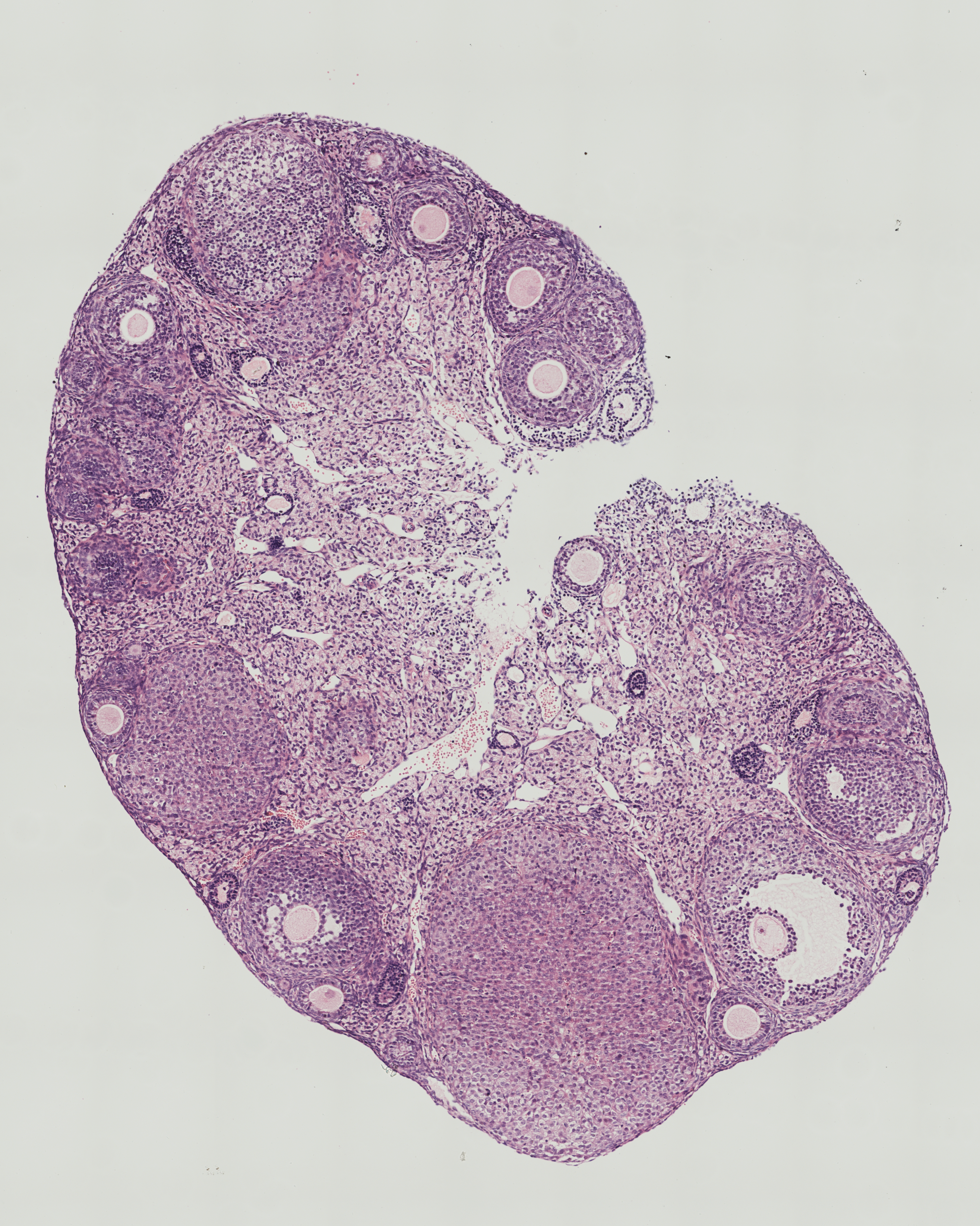

Supplement: Supplementary file 8 — Source data Fig. 3 [file 44321_2026_448_MOESM8_ESM.zip › Figure 3/3E/Ccdc174 CKO.tif]

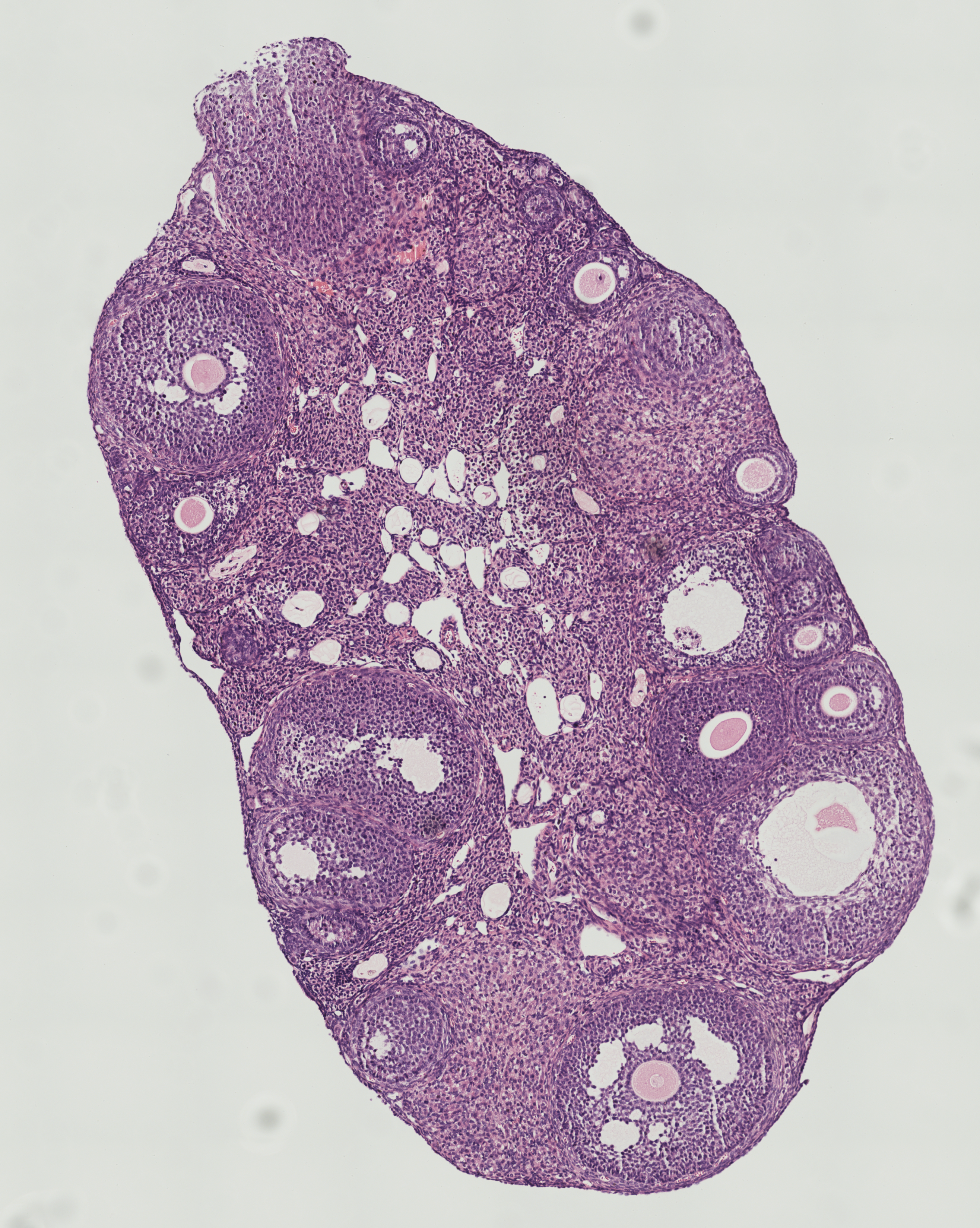

Supplement: Supplementary file 8 — Source data Fig. 3 [file 44321_2026_448_MOESM8_ESM.zip › Figure 3/3E/WT.tif]

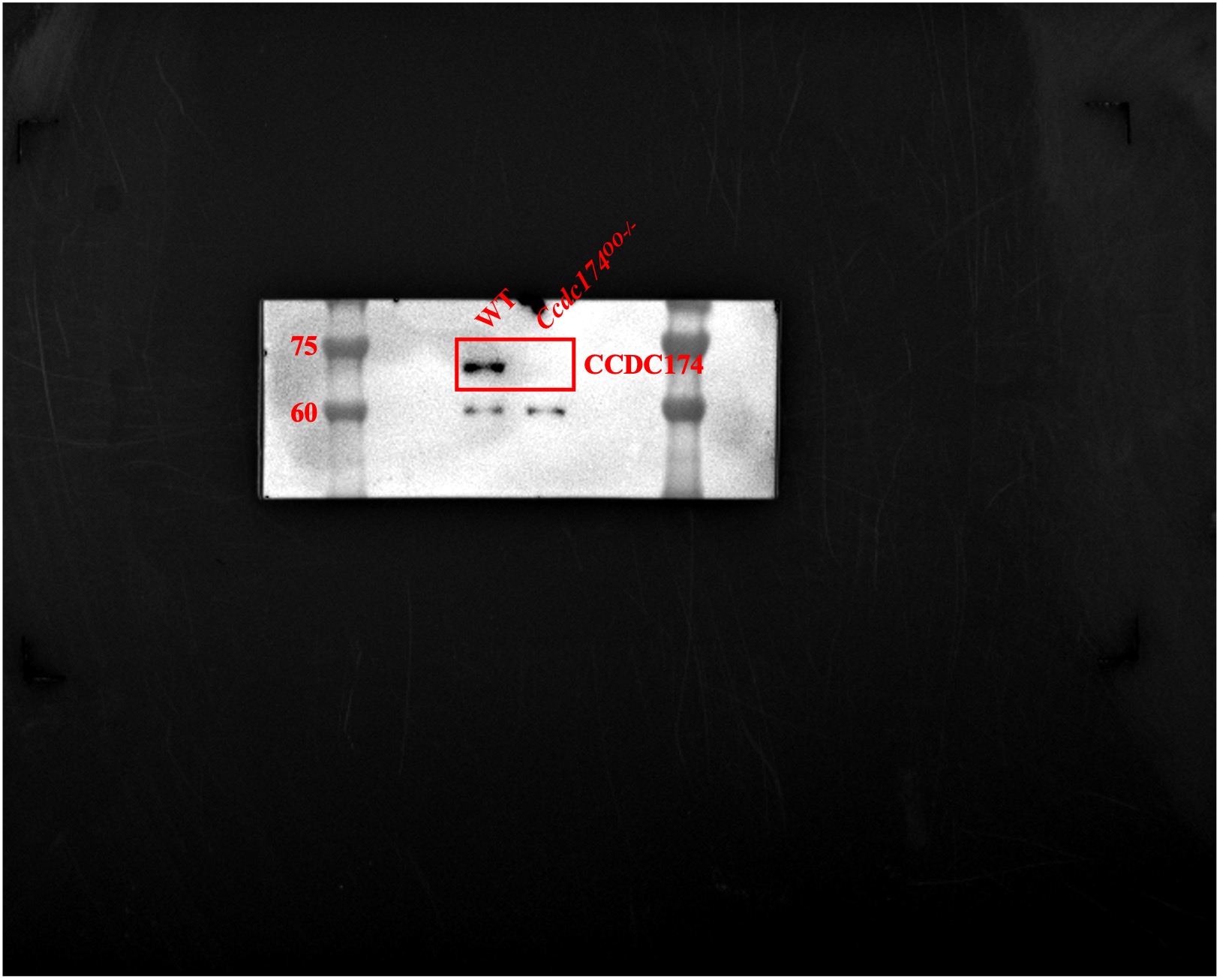

Supplement: Supplementary file 8 — Source data Fig. 3 [file 44321_2026_448_MOESM8_ESM.zip › Figure 3/3B/CCDC174.jpg]

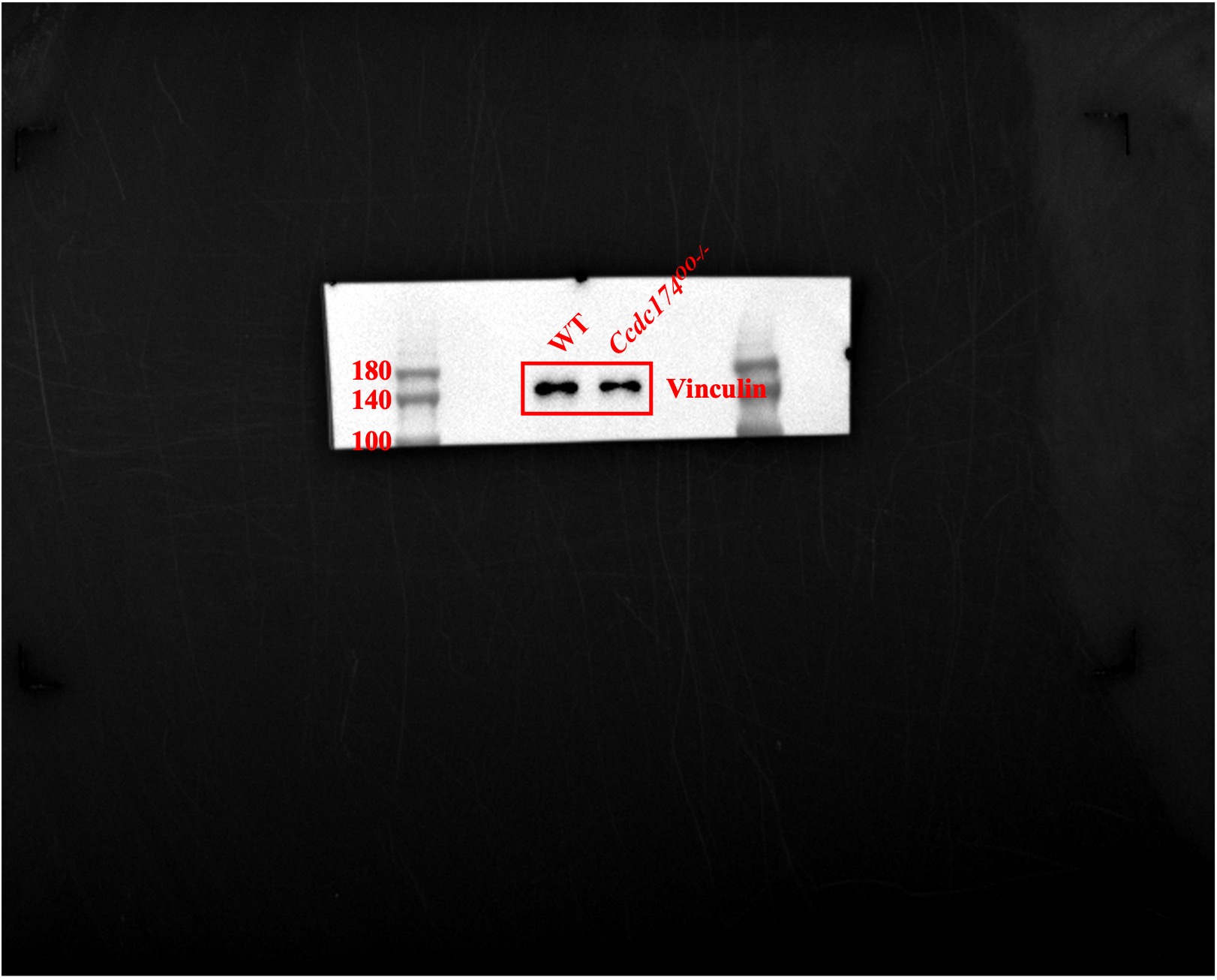

Supplement: Supplementary file 8 — Source data Fig. 3 [file 44321_2026_448_MOESM8_ESM.zip › Figure 3/3B/Vinculin.jpg]

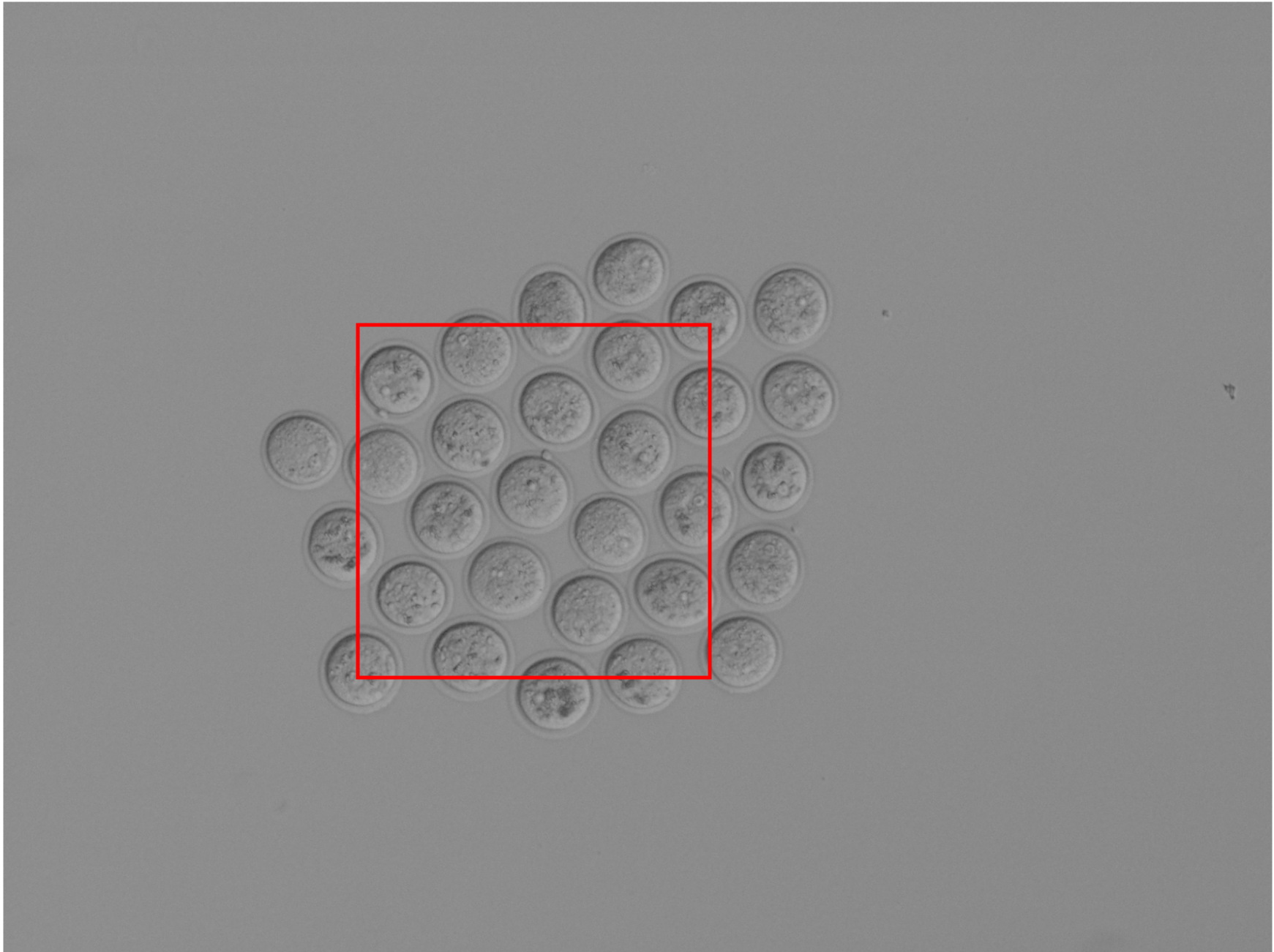

Supplement: Supplementary file 8 — Source data Fig. 3 [file 44321_2026_448_MOESM8_ESM.zip › Figure 3/3H/Ccdc174 CKO.png]

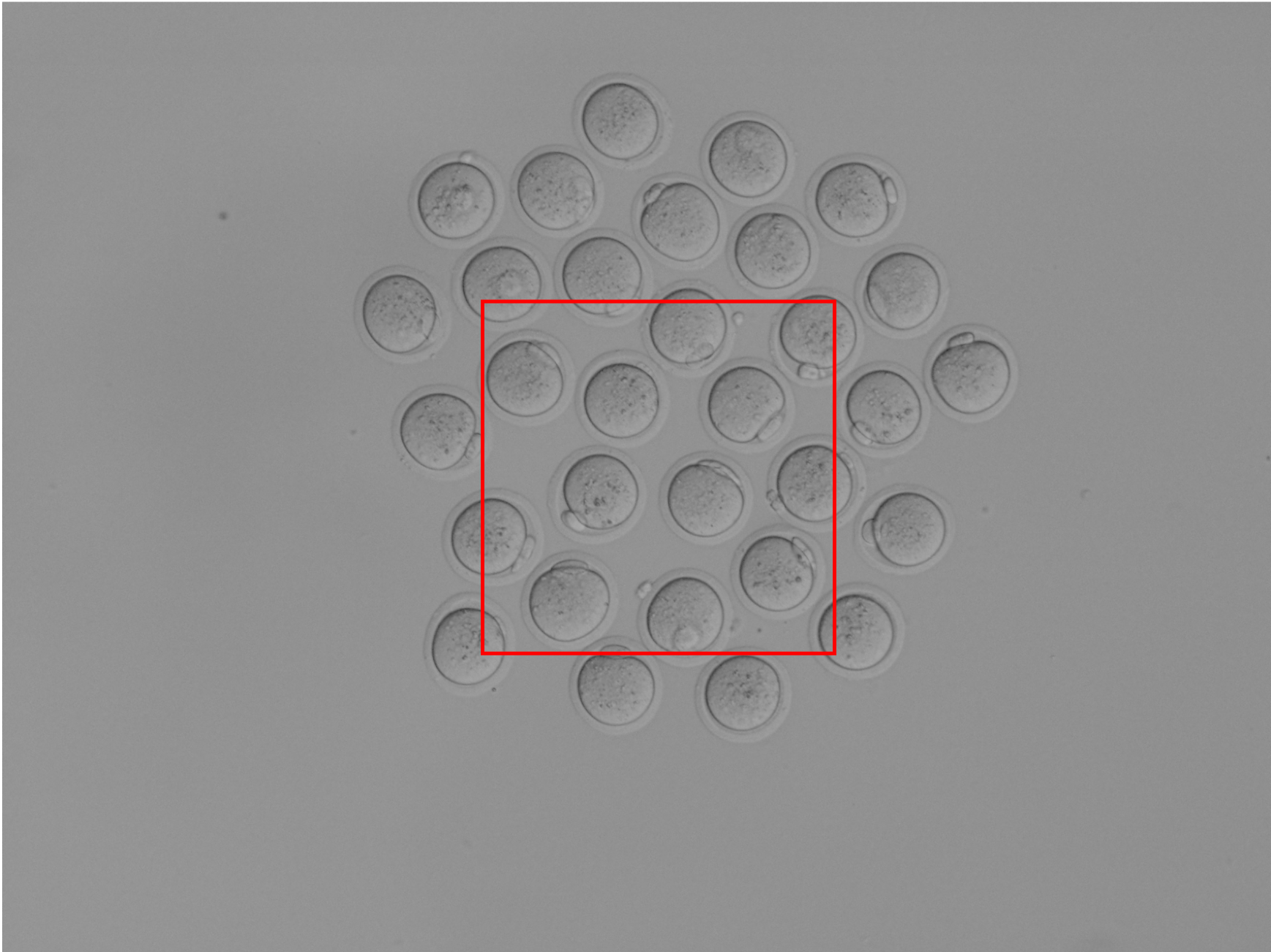

Supplement: Supplementary file 8 — Source data Fig. 3 [file 44321_2026_448_MOESM8_ESM.zip › Figure 3/3H/WT.png]

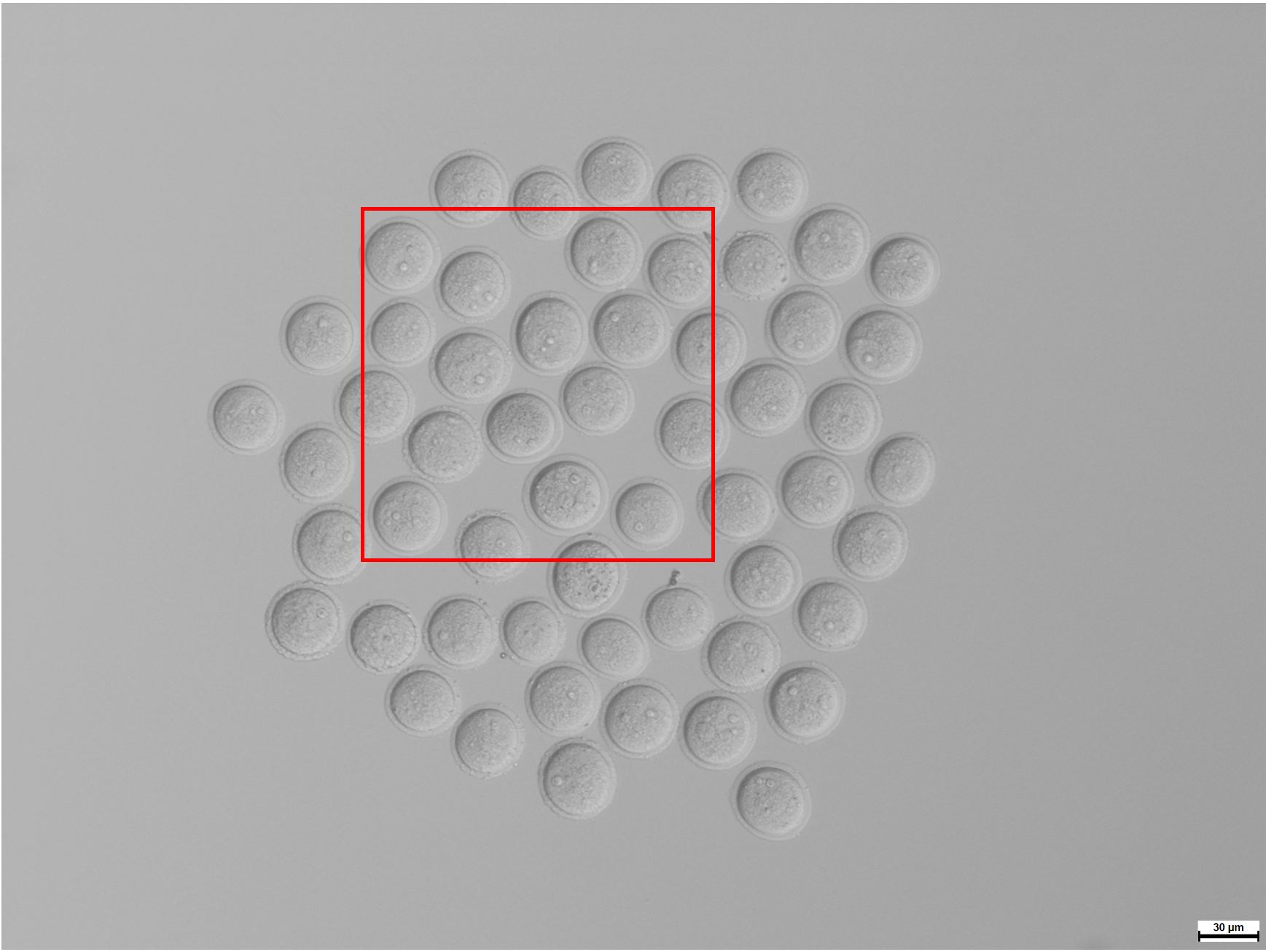

Supplement: Supplementary file 8 — Source data Fig. 3 [file 44321_2026_448_MOESM8_ESM.zip › Figure 3/3F/3 weeks-Ccdc174 CKO.png]

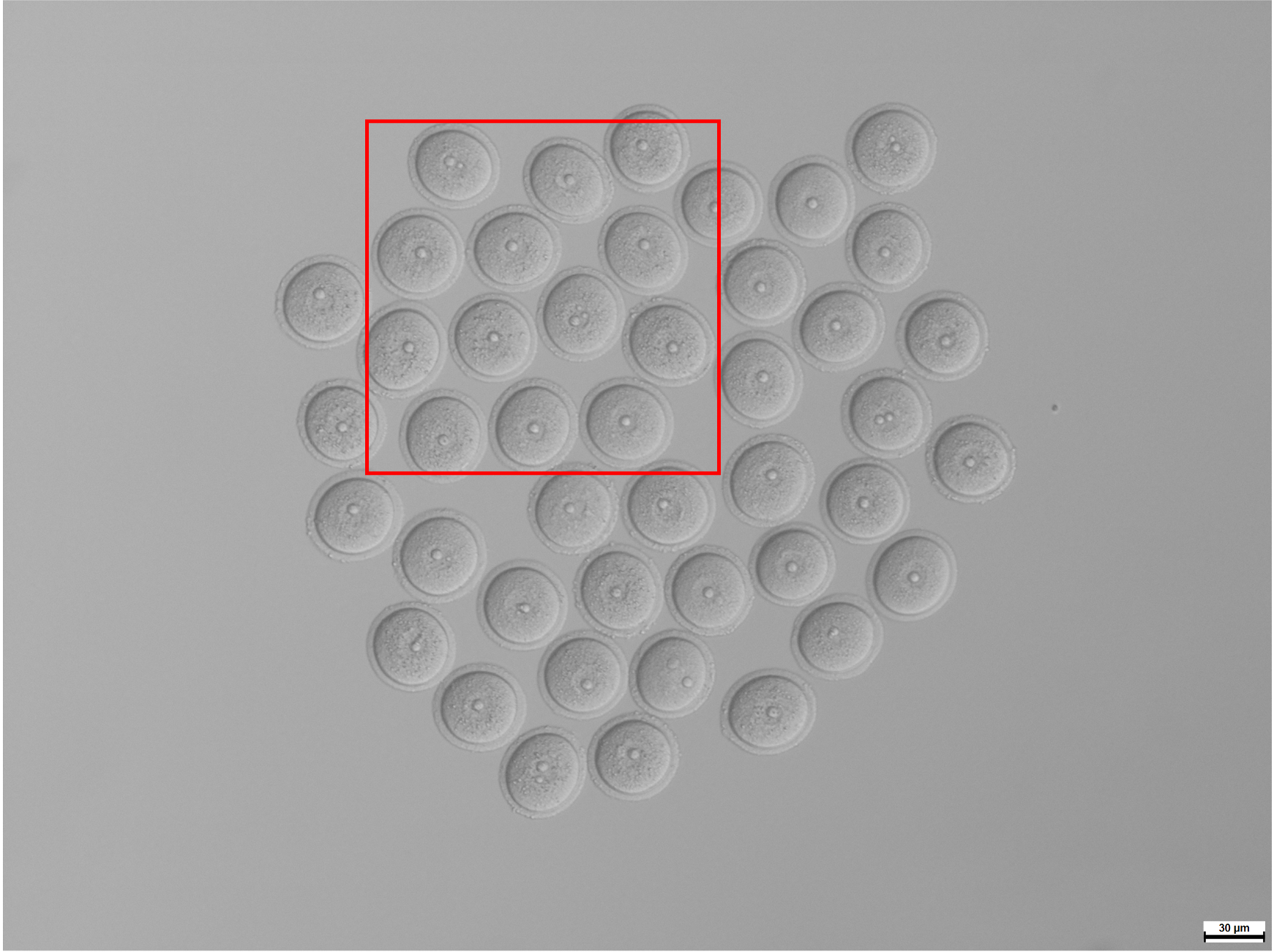

Supplement: Supplementary file 8 — Source data Fig. 3 [file 44321_2026_448_MOESM8_ESM.zip › Figure 3/3F/3 weeks-WT.png]

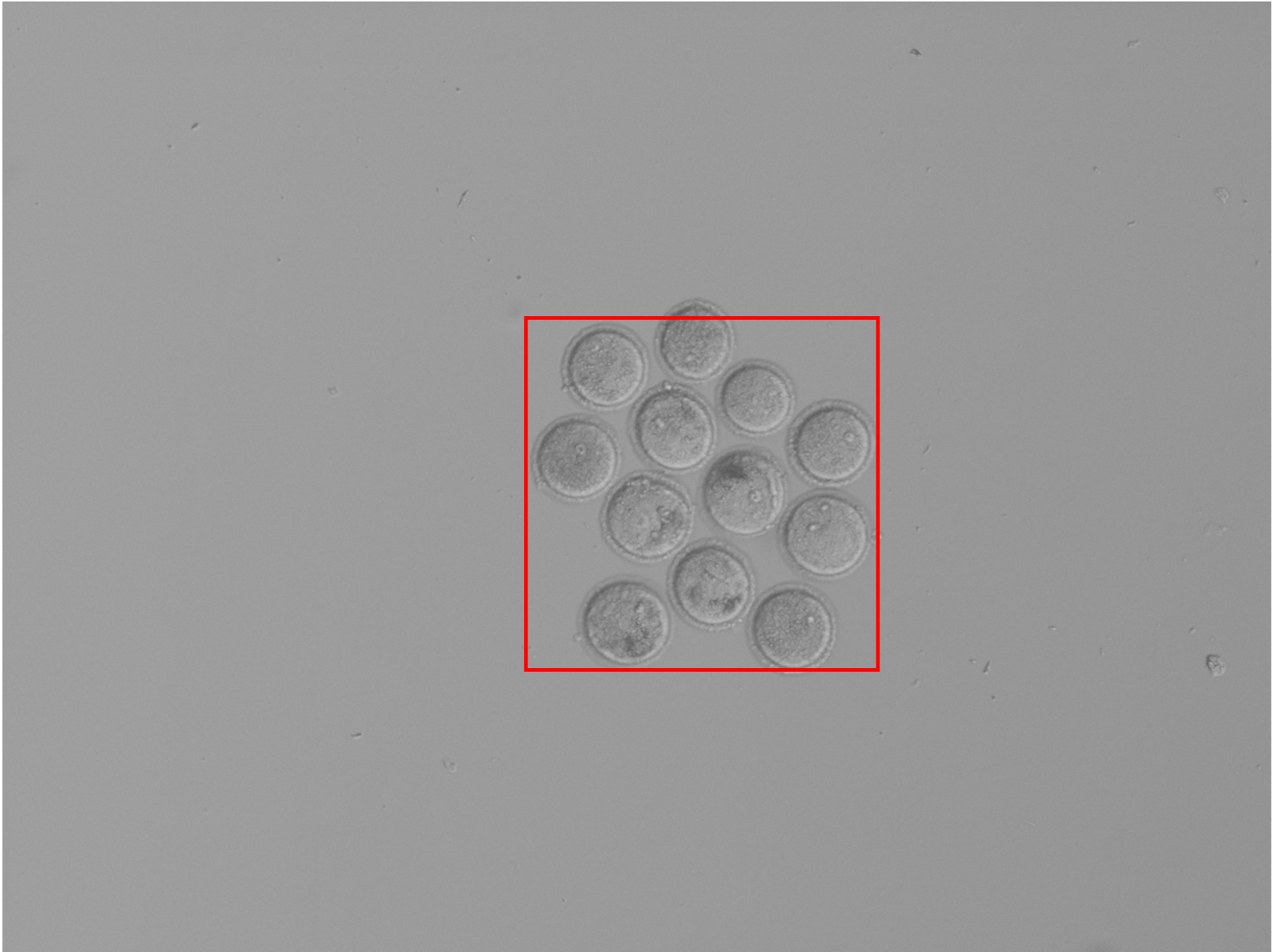

Supplement: Supplementary file 8 — Source data Fig. 3 [file 44321_2026_448_MOESM8_ESM.zip › Figure 3/3F/8 weeks-Ccdc174 CKO.png]

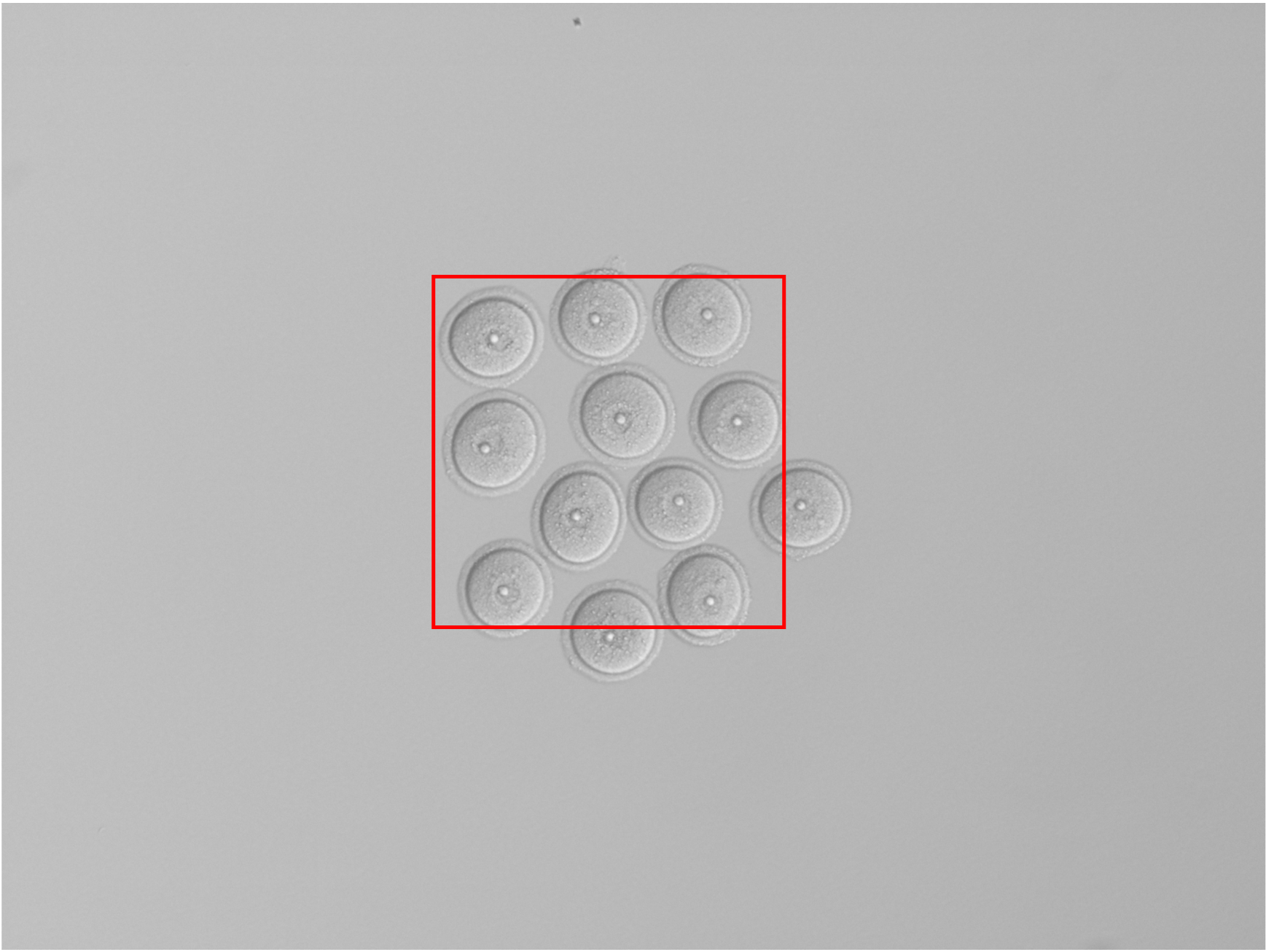

Supplement: Supplementary file 8 — Source data Fig. 3 [file 44321_2026_448_MOESM8_ESM.zip › Figure 3/3F/8 weeks-WT.png]

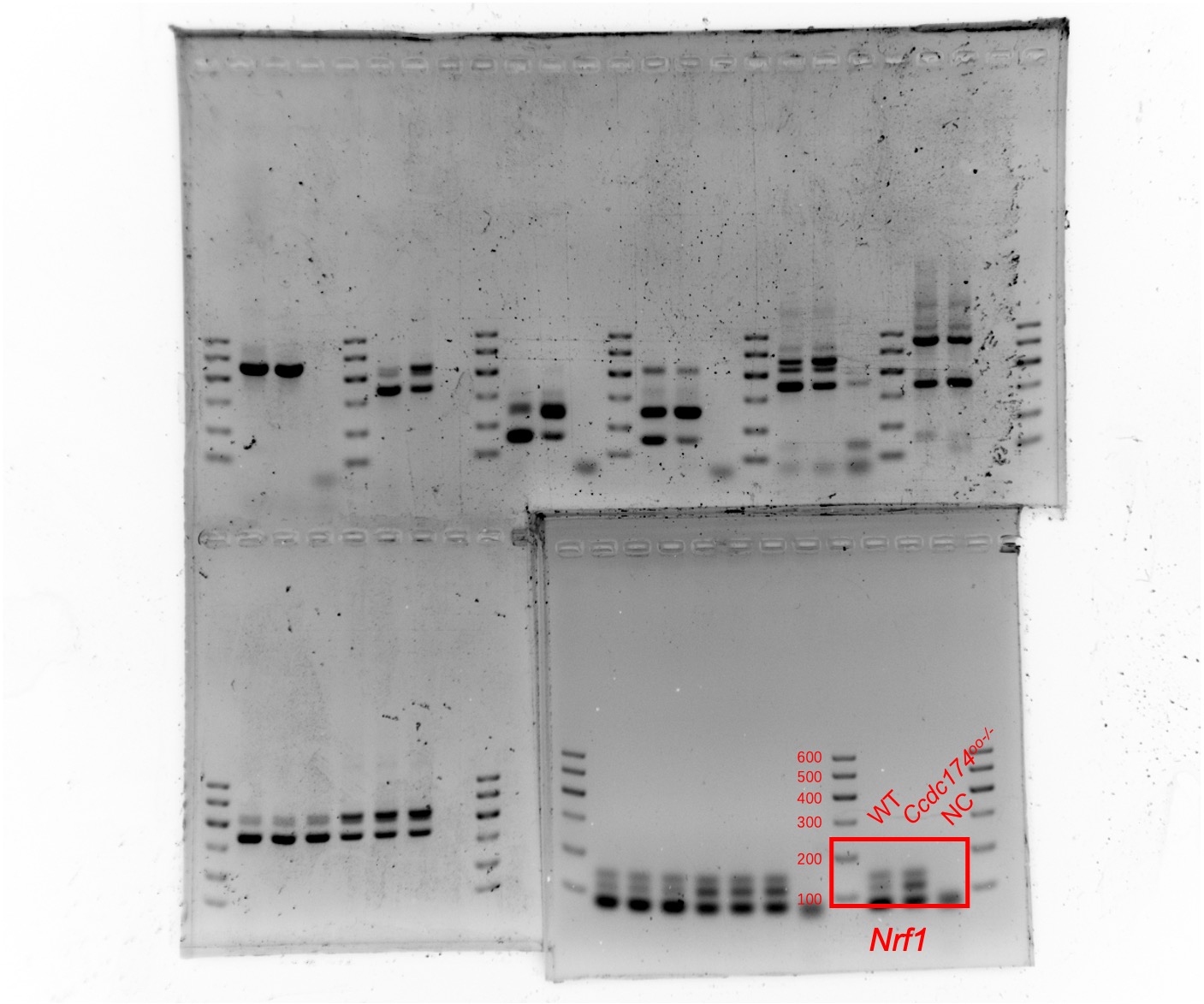

Supplement: Supplementary file 9 — Source data Fig. 5 [file 44321_2026_448_MOESM9_ESM.zip › Figure 5/5D/Figure 5D gel Nrf1.jpg]

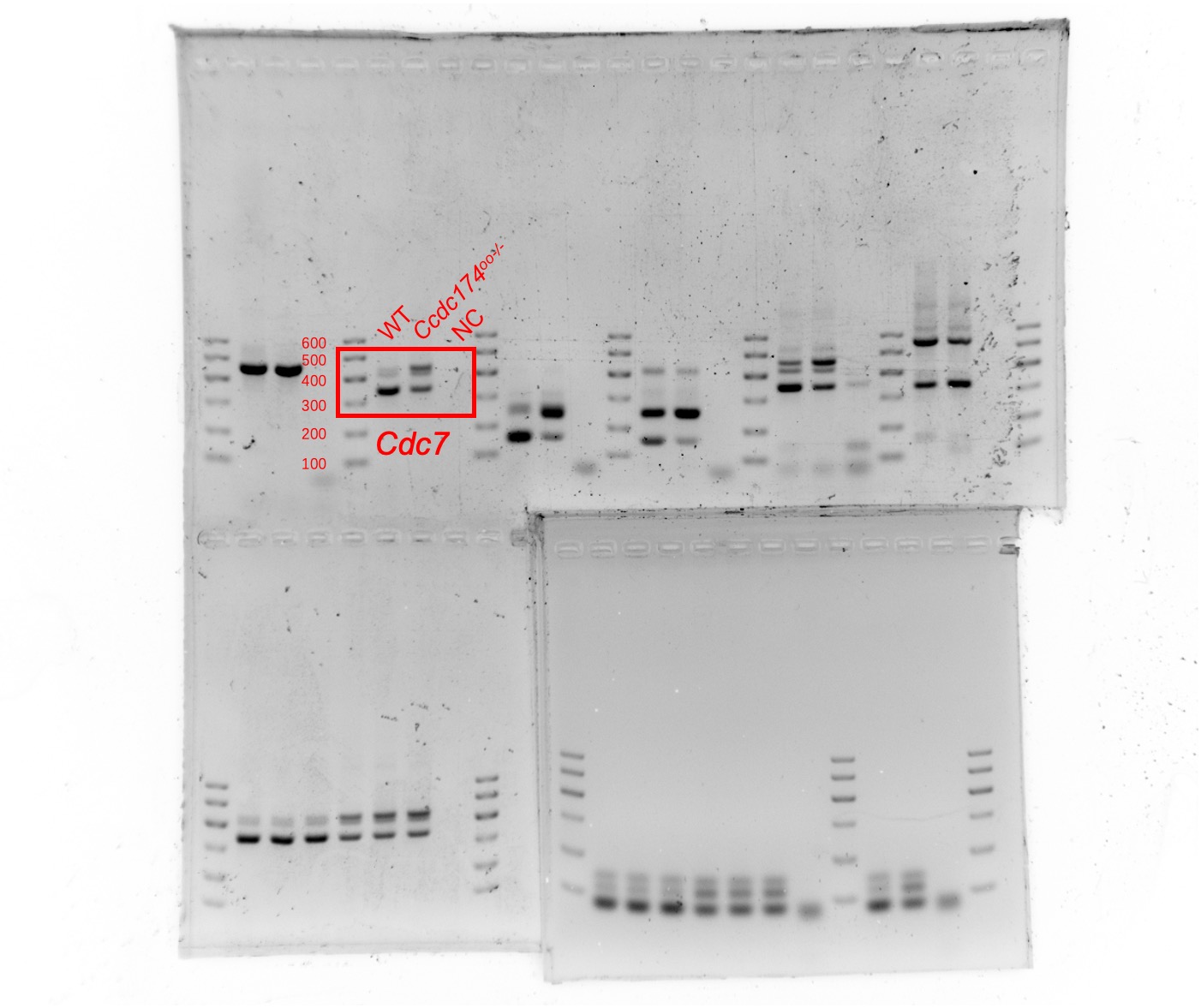

Supplement: Supplementary file 9 — Source data Fig. 5 [file 44321_2026_448_MOESM9_ESM.zip › Figure 5/5D/Figure 5D gel Cdc7.jpg]

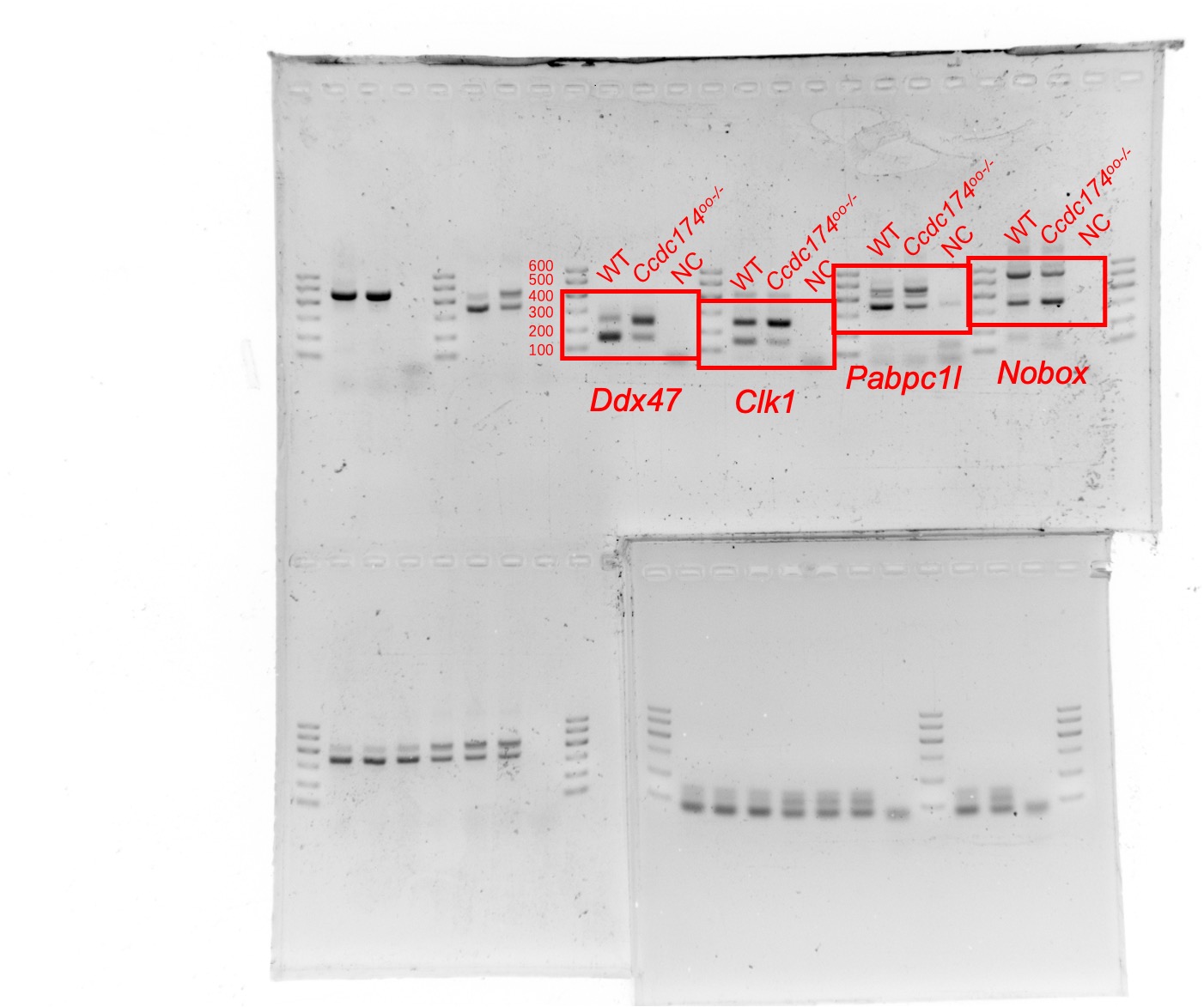

Supplement: Supplementary file 9 — Source data Fig. 5 [file 44321_2026_448_MOESM9_ESM.zip › Figure 5/5D/Figure 5D gel Ddx47 Clk1 Pabpc1l Nobox.jpg]

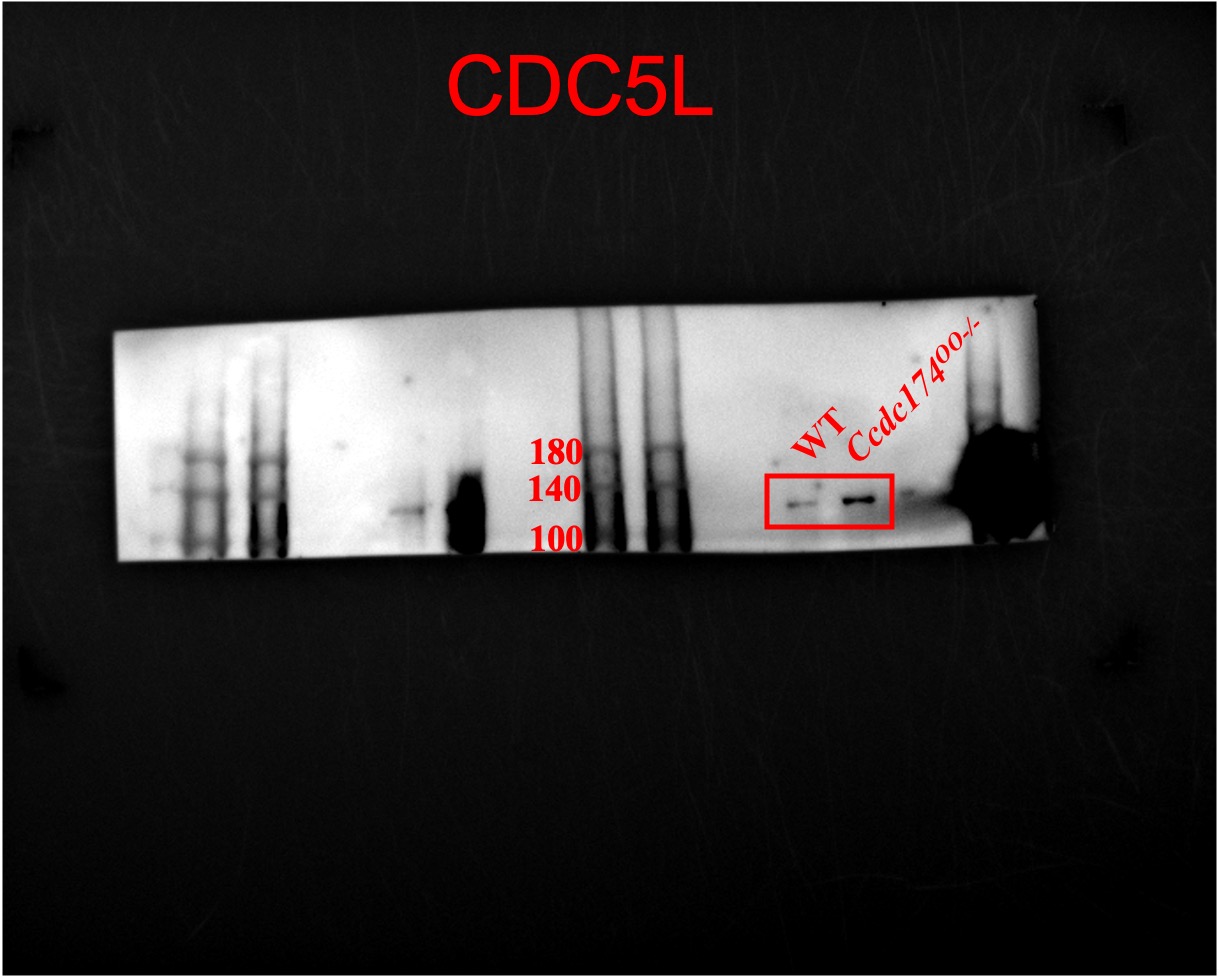

Supplement: Supplementary file 10 — Source data Fig. 6 [file 44321_2026_448_MOESM10_ESM.zip › Figure 6/6E/CDC5L.jpg]

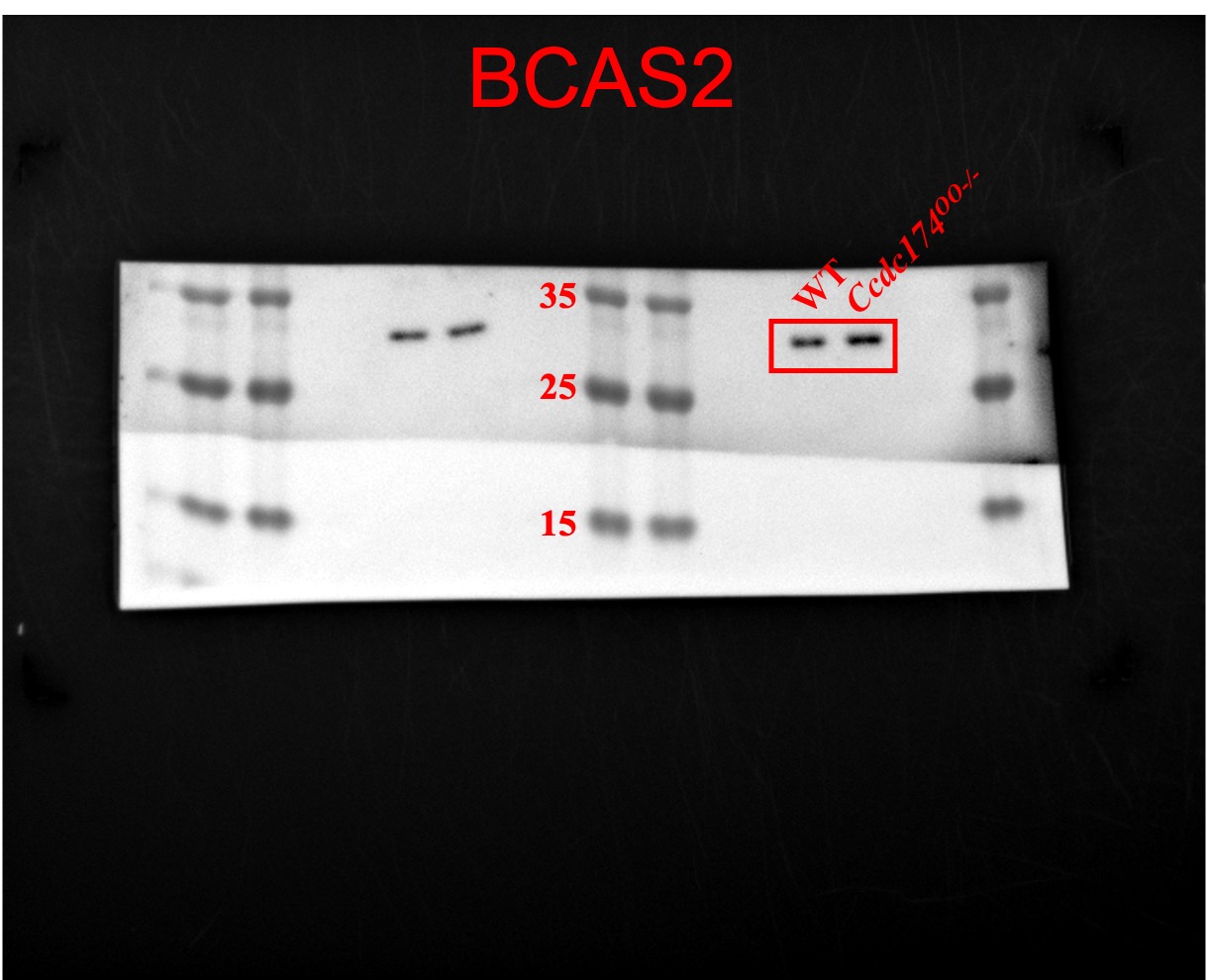

Supplement: Supplementary file 10 — Source data Fig. 6 [file 44321_2026_448_MOESM10_ESM.zip › Figure 6/6E/BCAS2.jpg]

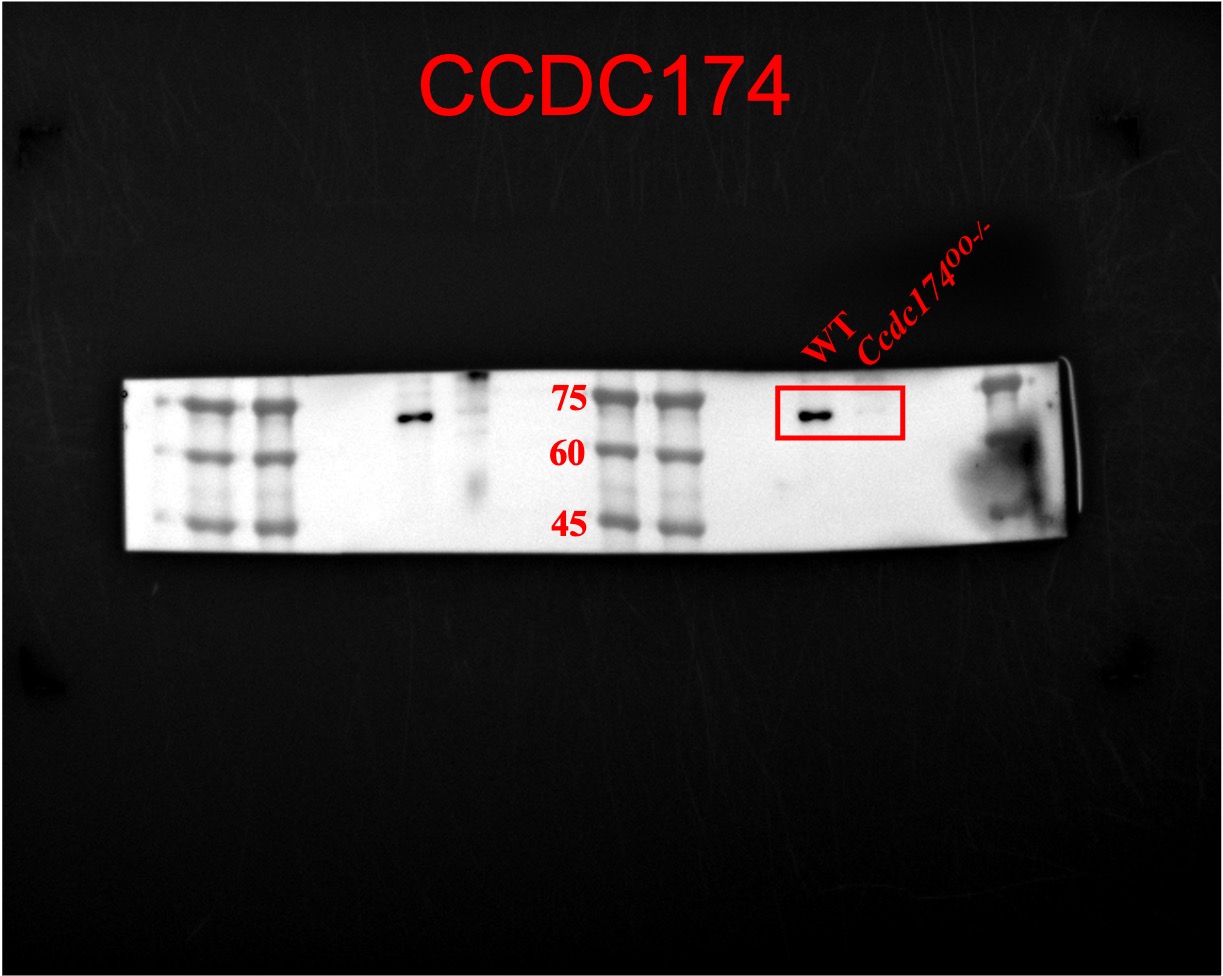

Supplement: Supplementary file 10 — Source data Fig. 6 [file 44321_2026_448_MOESM10_ESM.zip › Figure 6/6E/CCDC174.jpg]

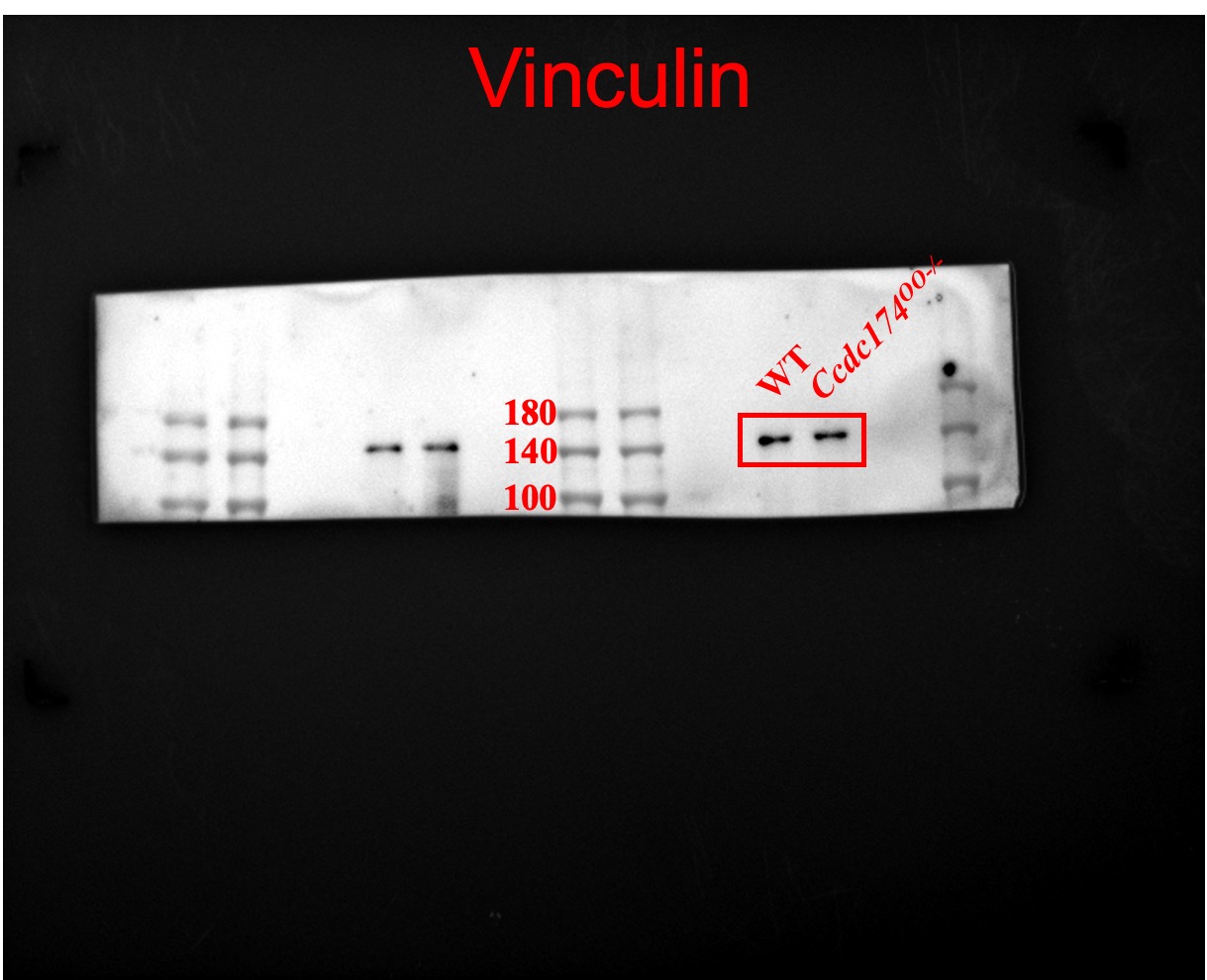

Supplement: Supplementary file 10 — Source data Fig. 6 [file 44321_2026_448_MOESM10_ESM.zip › Figure 6/6E/Vinculin.jpg]

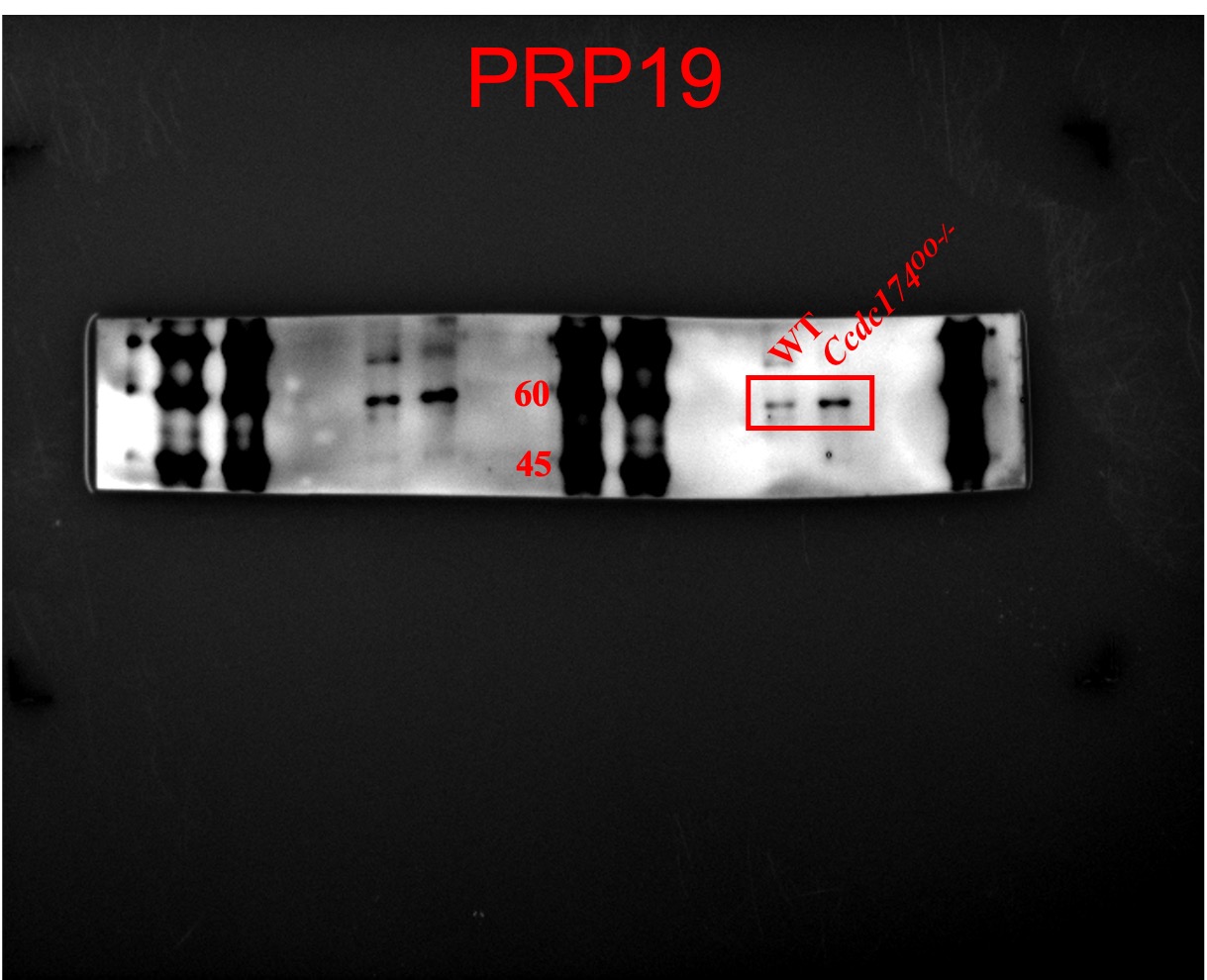

Supplement: Supplementary file 10 — Source data Fig. 6 [file 44321_2026_448_MOESM10_ESM.zip › Figure 6/6E/PRP19.jpg]

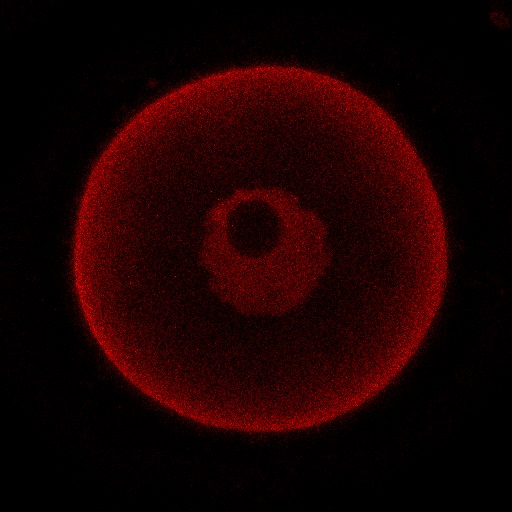

Supplement: Supplementary file 10 — Source data Fig. 6 [file 44321_2026_448_MOESM10_ESM.zip › Figure 6/6B/mPLRG1-FLAG+HA-CCDC174/FLAG.jpg]

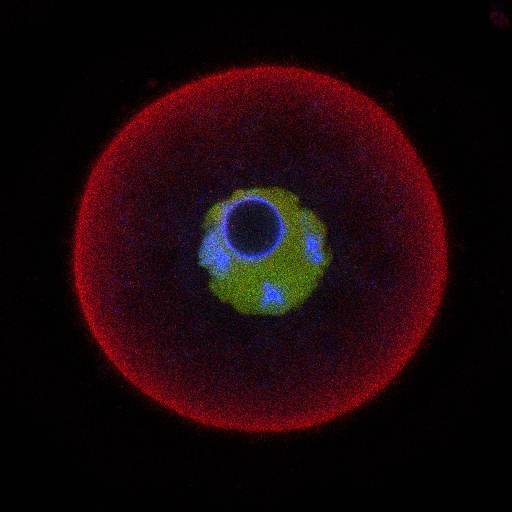

Supplement: Supplementary file 10 — Source data Fig. 6 [file 44321_2026_448_MOESM10_ESM.zip › Figure 6/6B/mPLRG1-FLAG+HA-CCDC174/Merge.jpg]

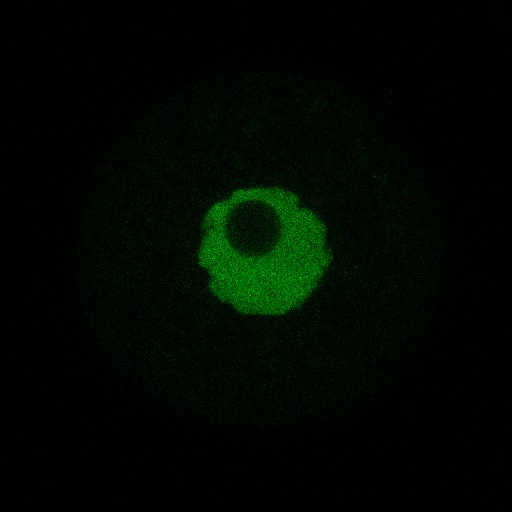

Supplement: Supplementary file 10 — Source data Fig. 6 [file 44321_2026_448_MOESM10_ESM.zip › Figure 6/6B/mPLRG1-FLAG+HA-CCDC174/HA.jpg]

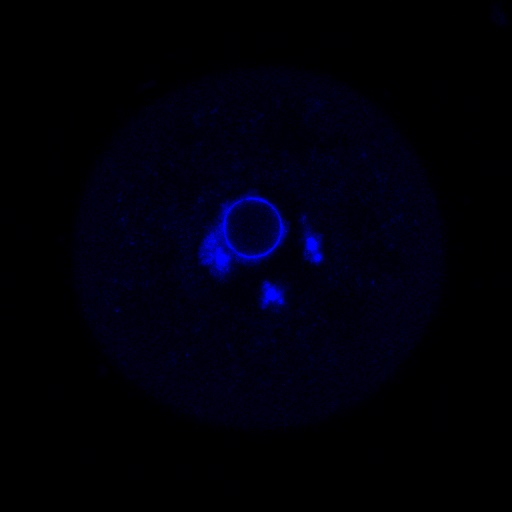

Supplement: Supplementary file 10 — Source data Fig. 6 [file 44321_2026_448_MOESM10_ESM.zip › Figure 6/6B/mPLRG1-FLAG+HA-CCDC174/Hoechst.jpg]

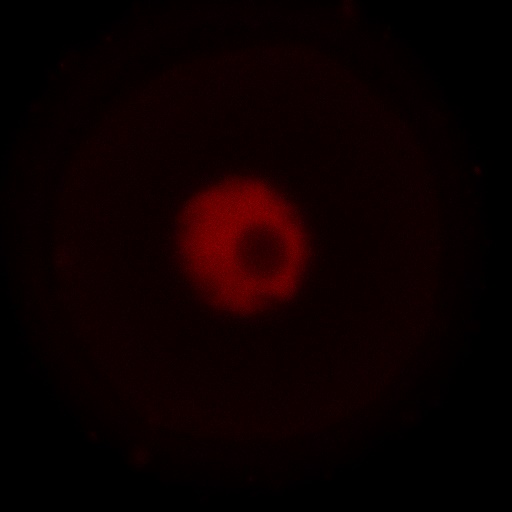

Supplement: Supplementary file 10 — Source data Fig. 6 [file 44321_2026_448_MOESM10_ESM.zip › Figure 6/6B/mCDC5L-FLAG+HA-CCDC174/FLAG.jpg]

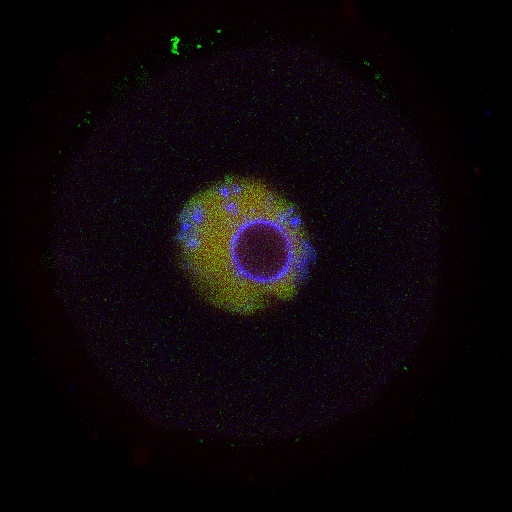

Supplement: Supplementary file 10 — Source data Fig. 6 [file 44321_2026_448_MOESM10_ESM.zip › Figure 6/6B/mCDC5L-FLAG+HA-CCDC174/Merge.jpg]

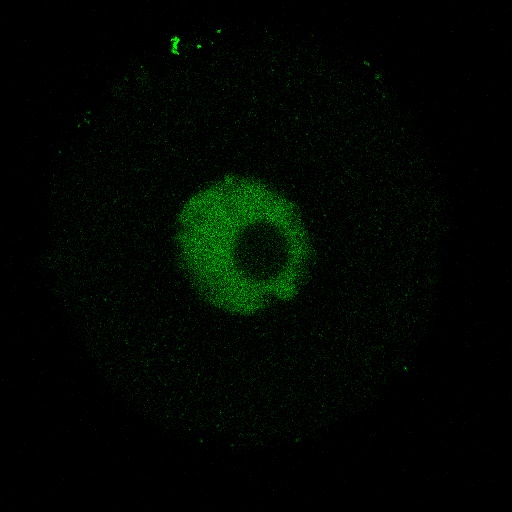

Supplement: Supplementary file 10 — Source data Fig. 6 [file 44321_2026_448_MOESM10_ESM.zip › Figure 6/6B/mCDC5L-FLAG+HA-CCDC174/HA.jpg]

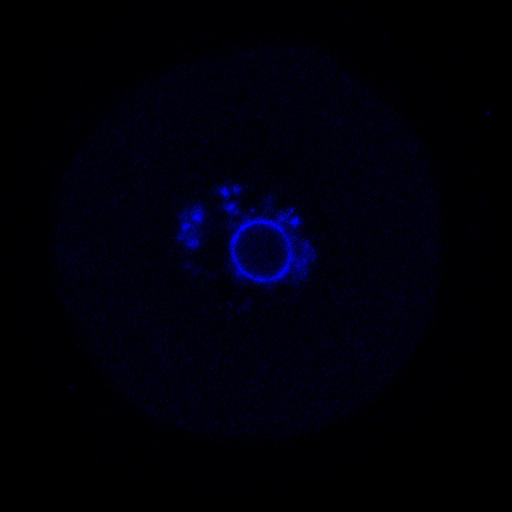

Supplement: Supplementary file 10 — Source data Fig. 6 [file 44321_2026_448_MOESM10_ESM.zip › Figure 6/6B/mCDC5L-FLAG+HA-CCDC174/Hoechst.jpg]

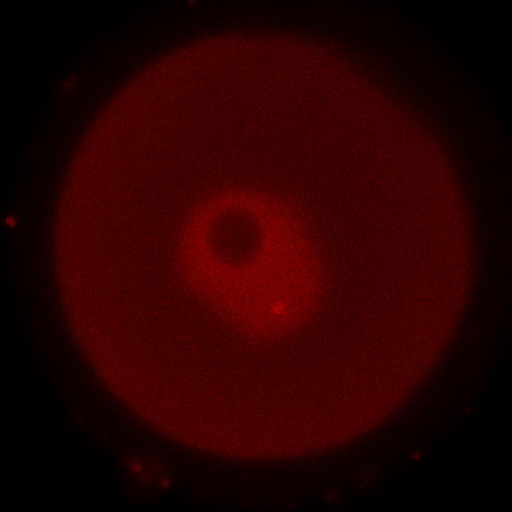

Supplement: Supplementary file 10 — Source data Fig. 6 [file 44321_2026_448_MOESM10_ESM.zip › Figure 6/6B/mPRP19-FLAG+HA-mCCDC174/FLAG.jpg]

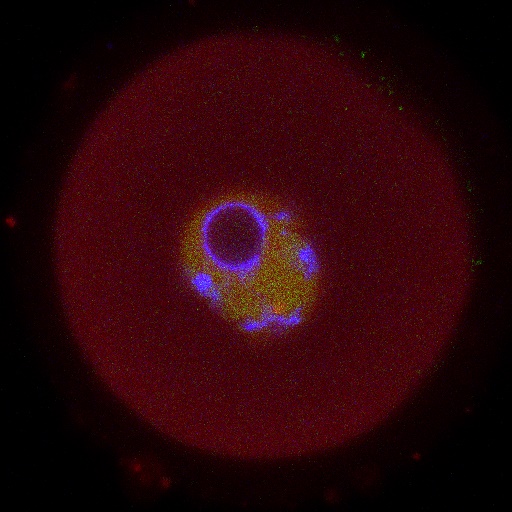

Supplement: Supplementary file 10 — Source data Fig. 6 [file 44321_2026_448_MOESM10_ESM.zip › Figure 6/6B/mPRP19-FLAG+HA-mCCDC174/Merge.jpg]

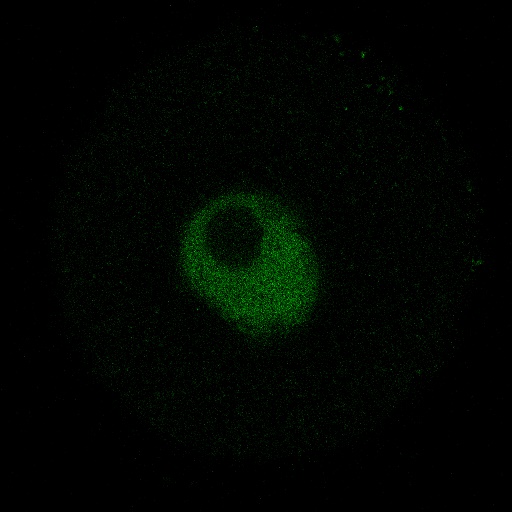

Supplement: Supplementary file 10 — Source data Fig. 6 [file 44321_2026_448_MOESM10_ESM.zip › Figure 6/6B/mPRP19-FLAG+HA-mCCDC174/HA.jpg]

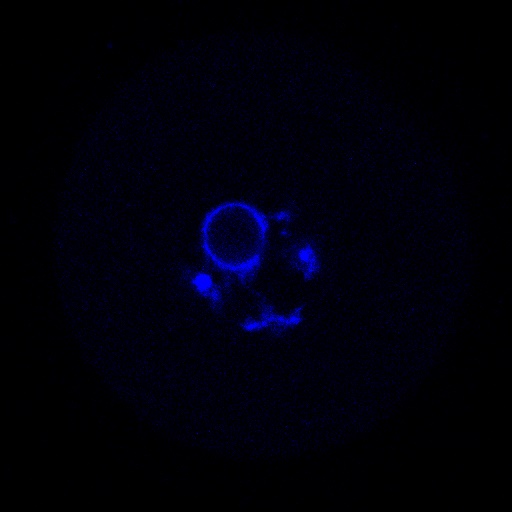

Supplement: Supplementary file 10 — Source data Fig. 6 [file 44321_2026_448_MOESM10_ESM.zip › Figure 6/6B/mPRP19-FLAG+HA-mCCDC174/Hoechst.jpg]

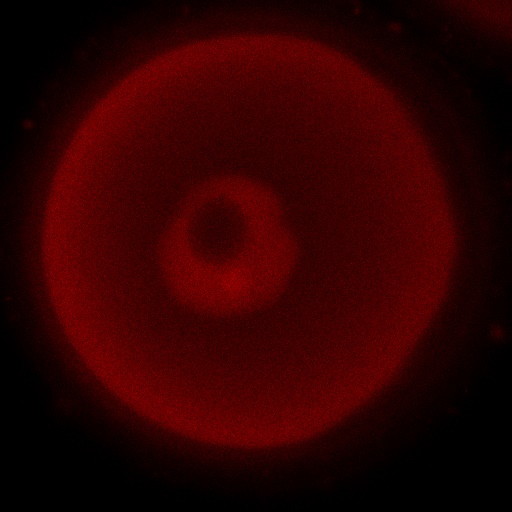

Supplement: Supplementary file 10 — Source data Fig. 6 [file 44321_2026_448_MOESM10_ESM.zip › Figure 6/6B/mBCAS2-FLAG+ HA-mCCDC174/FLAG.jpg]

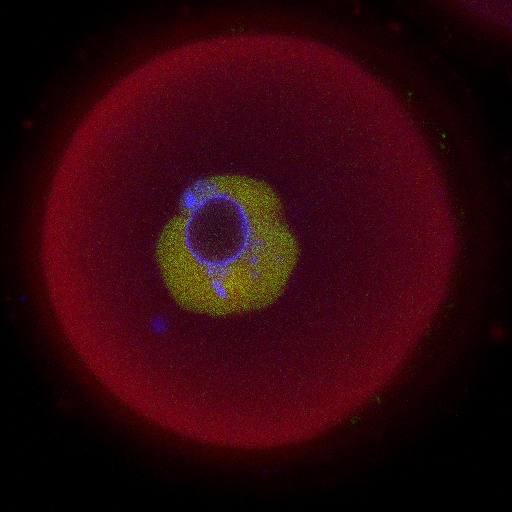

Supplement: Supplementary file 10 — Source data Fig. 6 [file 44321_2026_448_MOESM10_ESM.zip › Figure 6/6B/mBCAS2-FLAG+ HA-mCCDC174/Merge.jpg]

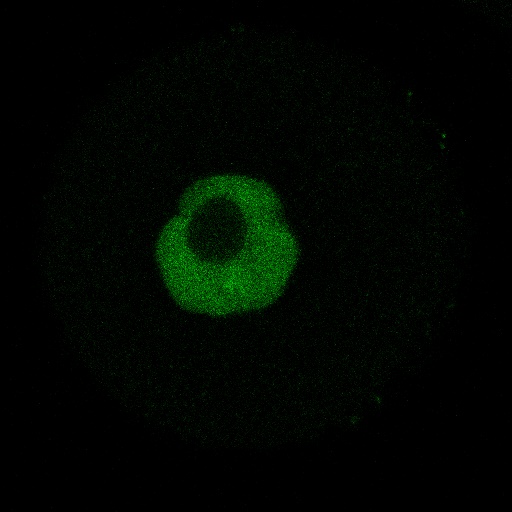

Supplement: Supplementary file 10 — Source data Fig. 6 [file 44321_2026_448_MOESM10_ESM.zip › Figure 6/6B/mBCAS2-FLAG+ HA-mCCDC174/HA.jpg]

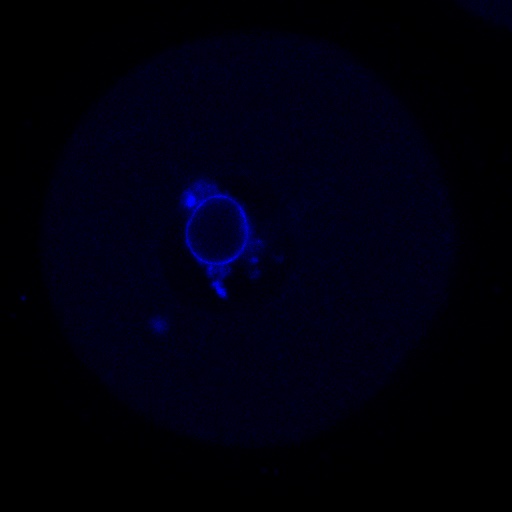

Supplement: Supplementary file 10 — Source data Fig. 6 [file 44321_2026_448_MOESM10_ESM.zip › Figure 6/6B/mBCAS2-FLAG+ HA-mCCDC174/Hoechst.jpg]

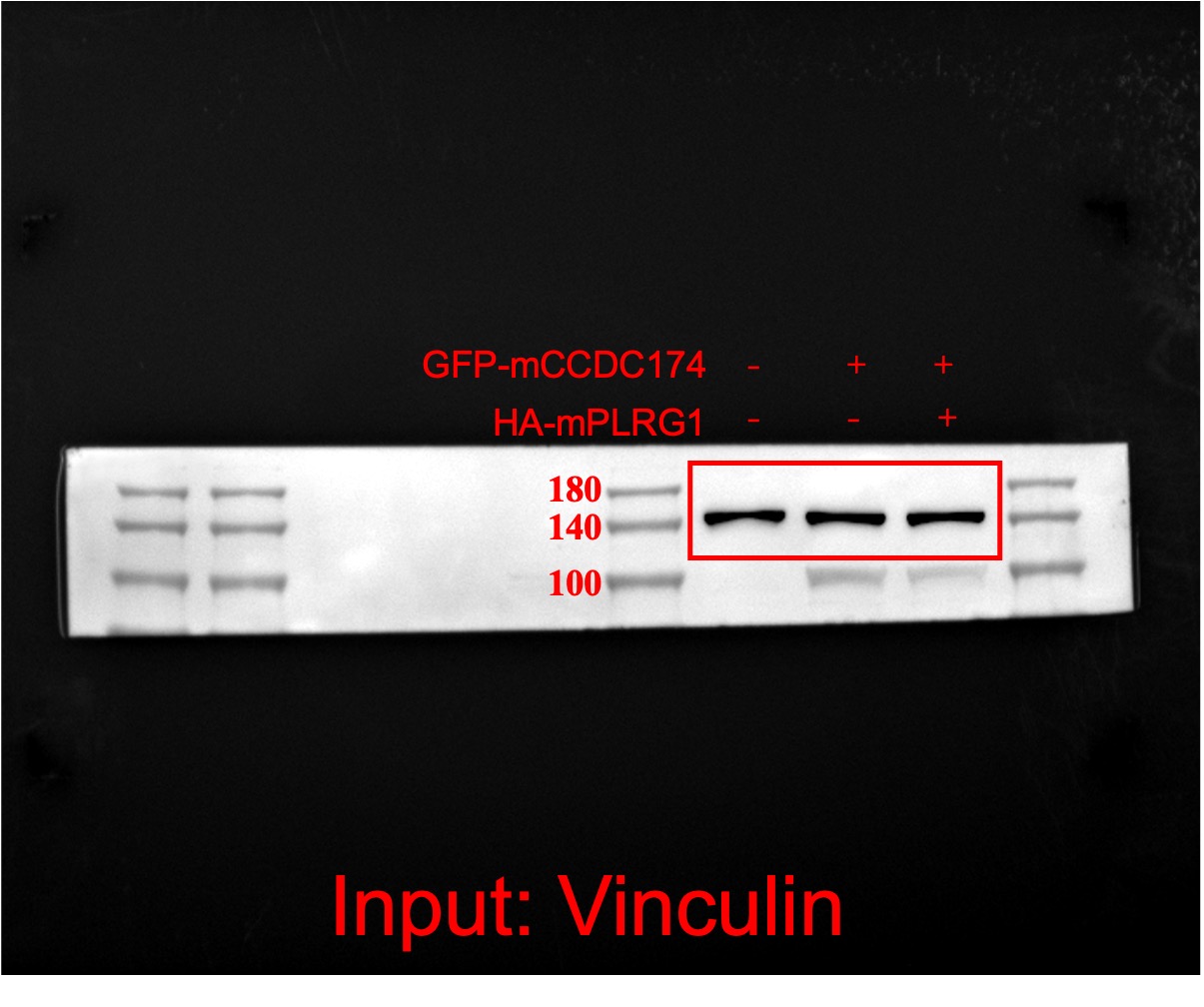

Supplement: Supplementary file 10 — Source data Fig. 6 [file 44321_2026_448_MOESM10_ESM.zip › Figure 6/6C/PLRG1 IP/PLRG1 Input Vinculin.jpg]

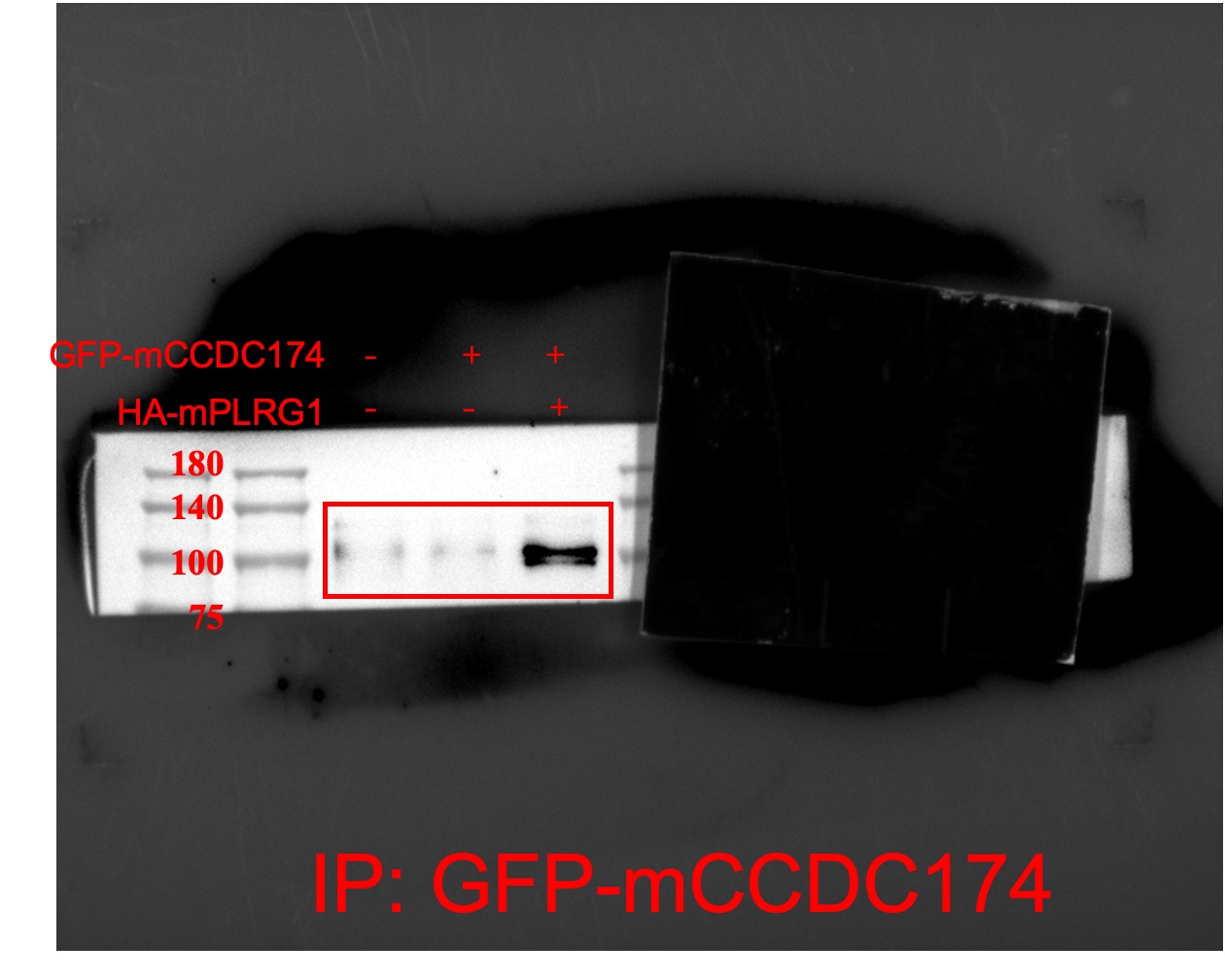

Supplement: Supplementary file 10 — Source data Fig. 6 [file 44321_2026_448_MOESM10_ESM.zip › Figure 6/6C/PLRG1 IP/PLRG1 IP GFP-mCCDC174.jpg]

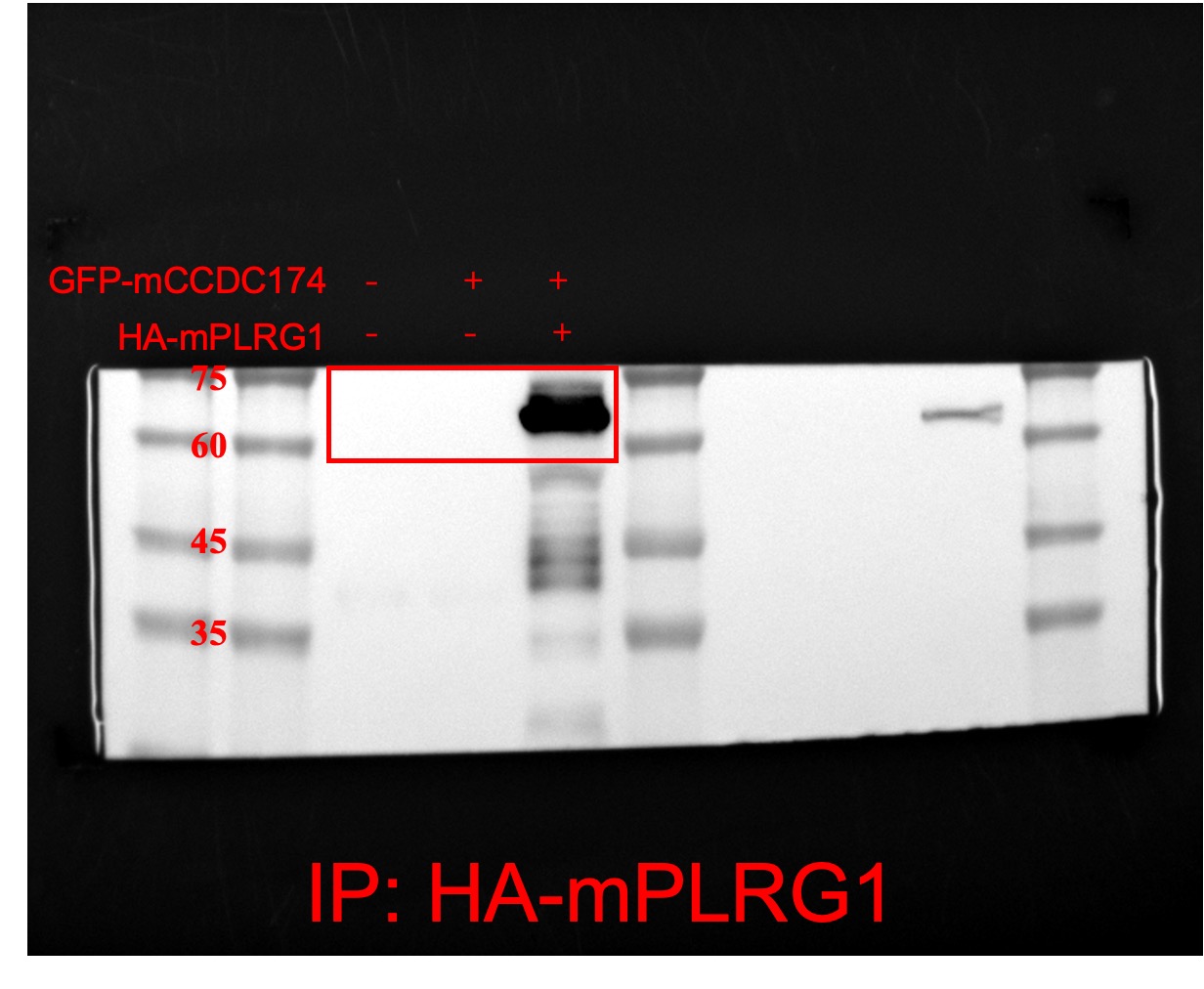

Supplement: Supplementary file 10 — Source data Fig. 6 [file 44321_2026_448_MOESM10_ESM.zip › Figure 6/6C/PLRG1 IP/PLRG1 IP HA-mPLRG1.jpg]

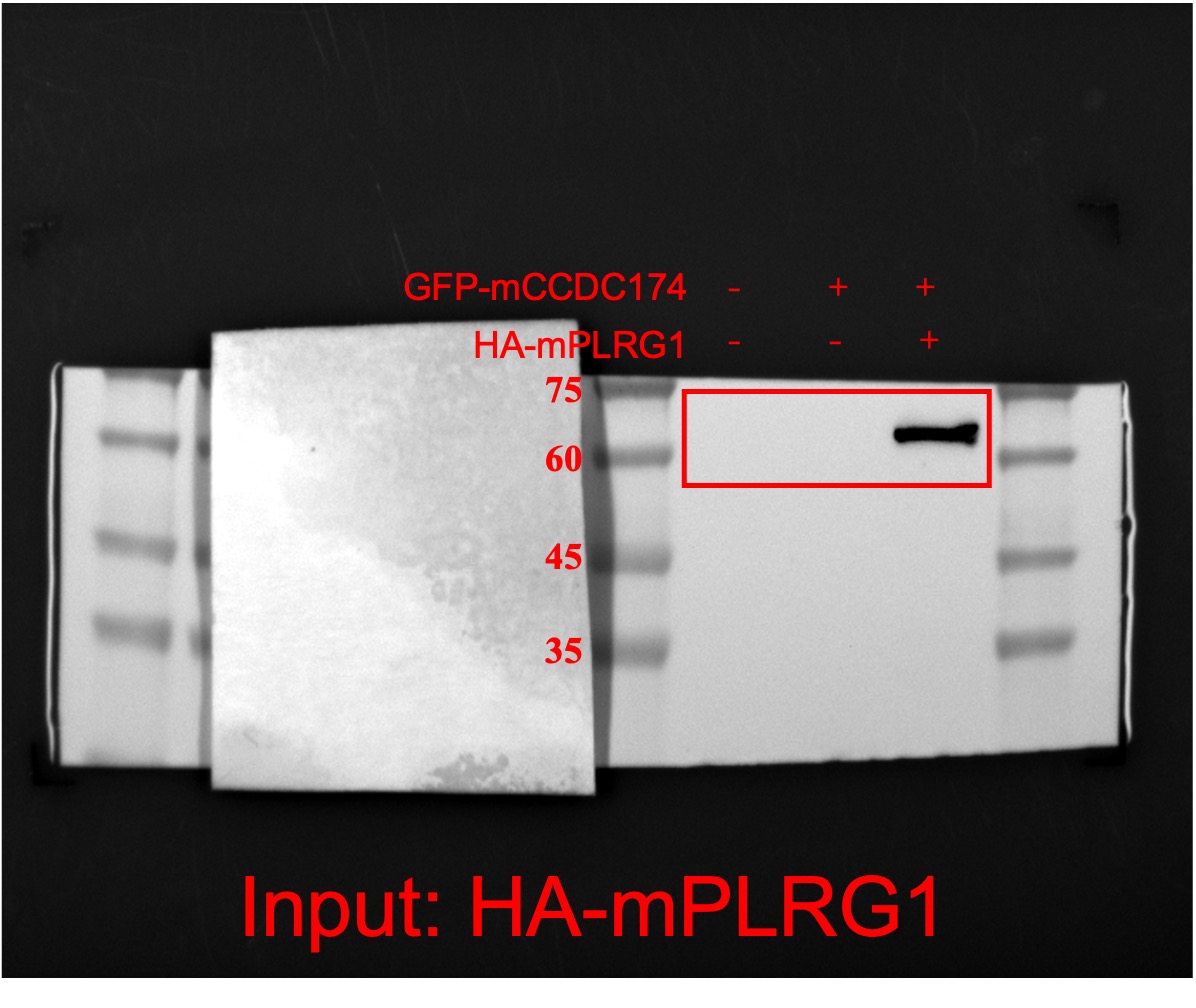

Supplement: Supplementary file 10 — Source data Fig. 6 [file 44321_2026_448_MOESM10_ESM.zip › Figure 6/6C/PLRG1 IP/PLRG1 Input HA-mPLRG1.jpg]

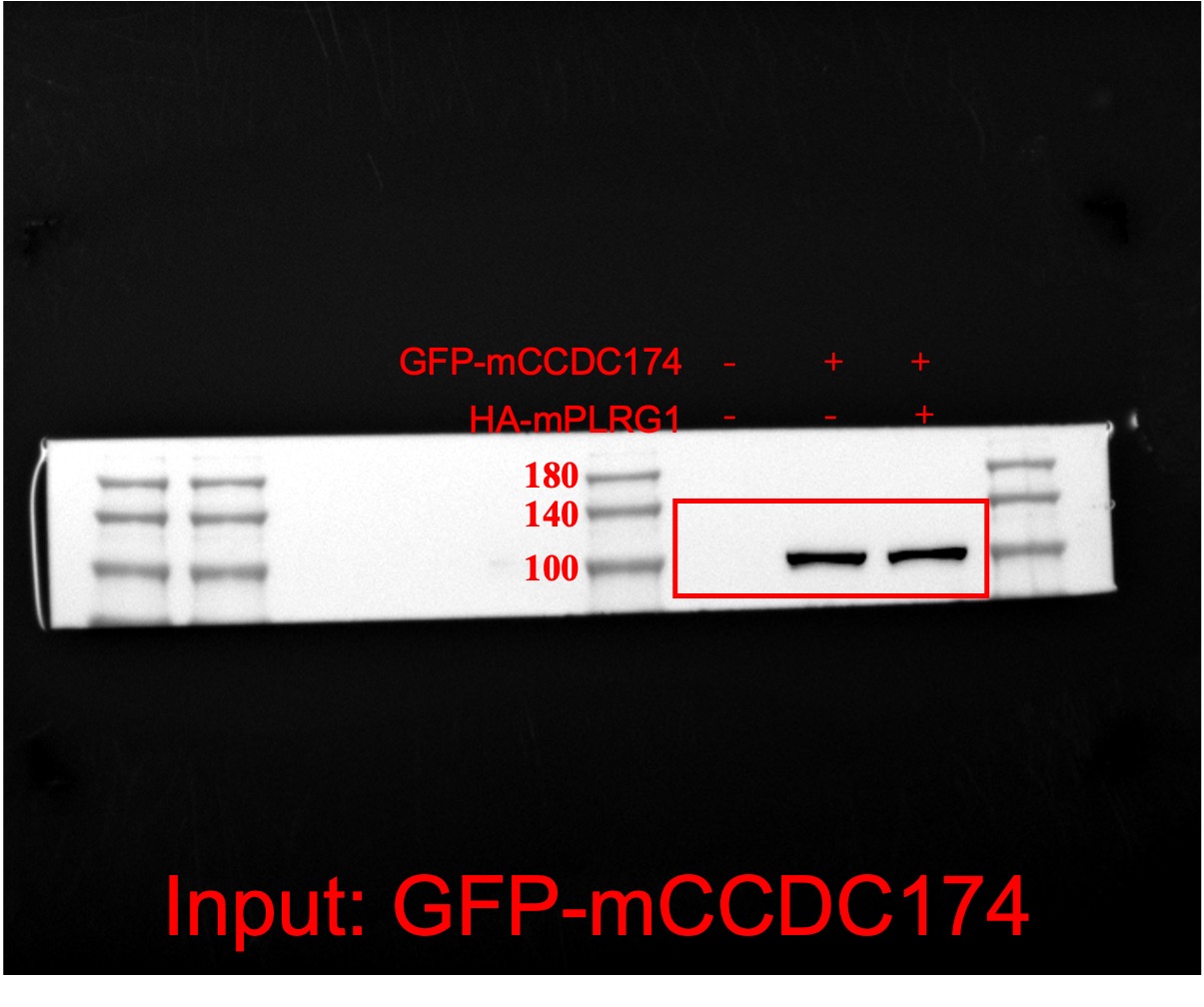

Supplement: Supplementary file 10 — Source data Fig. 6 [file 44321_2026_448_MOESM10_ESM.zip › Figure 6/6C/PLRG1 IP/PLRG1 Input GFP-mCCDC174.jpg]

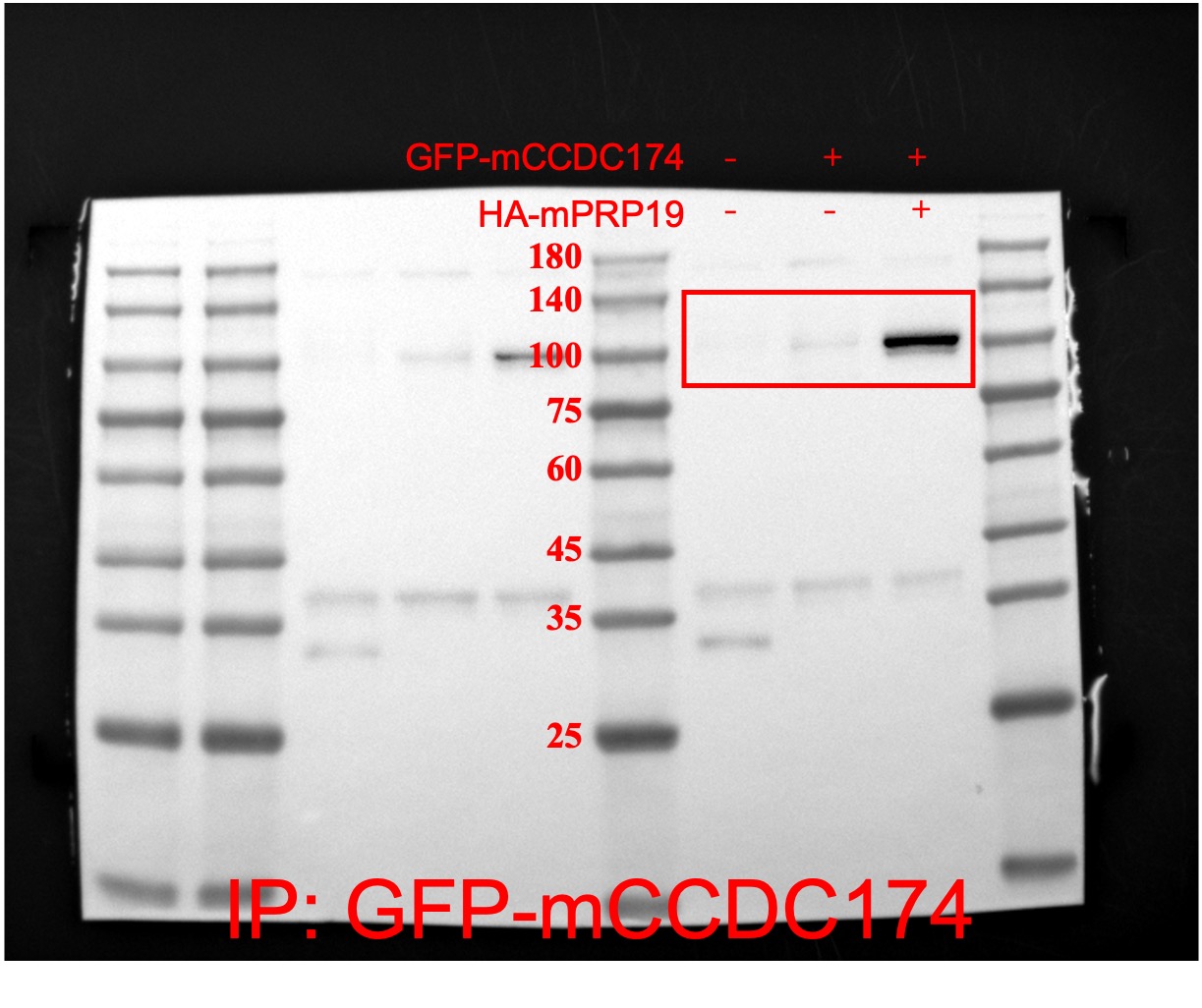

Supplement: Supplementary file 10 — Source data Fig. 6 [file 44321_2026_448_MOESM10_ESM.zip › Figure 6/6C/PRP19 IP/PRP19 IP GFP-mCCDC174.jpg]

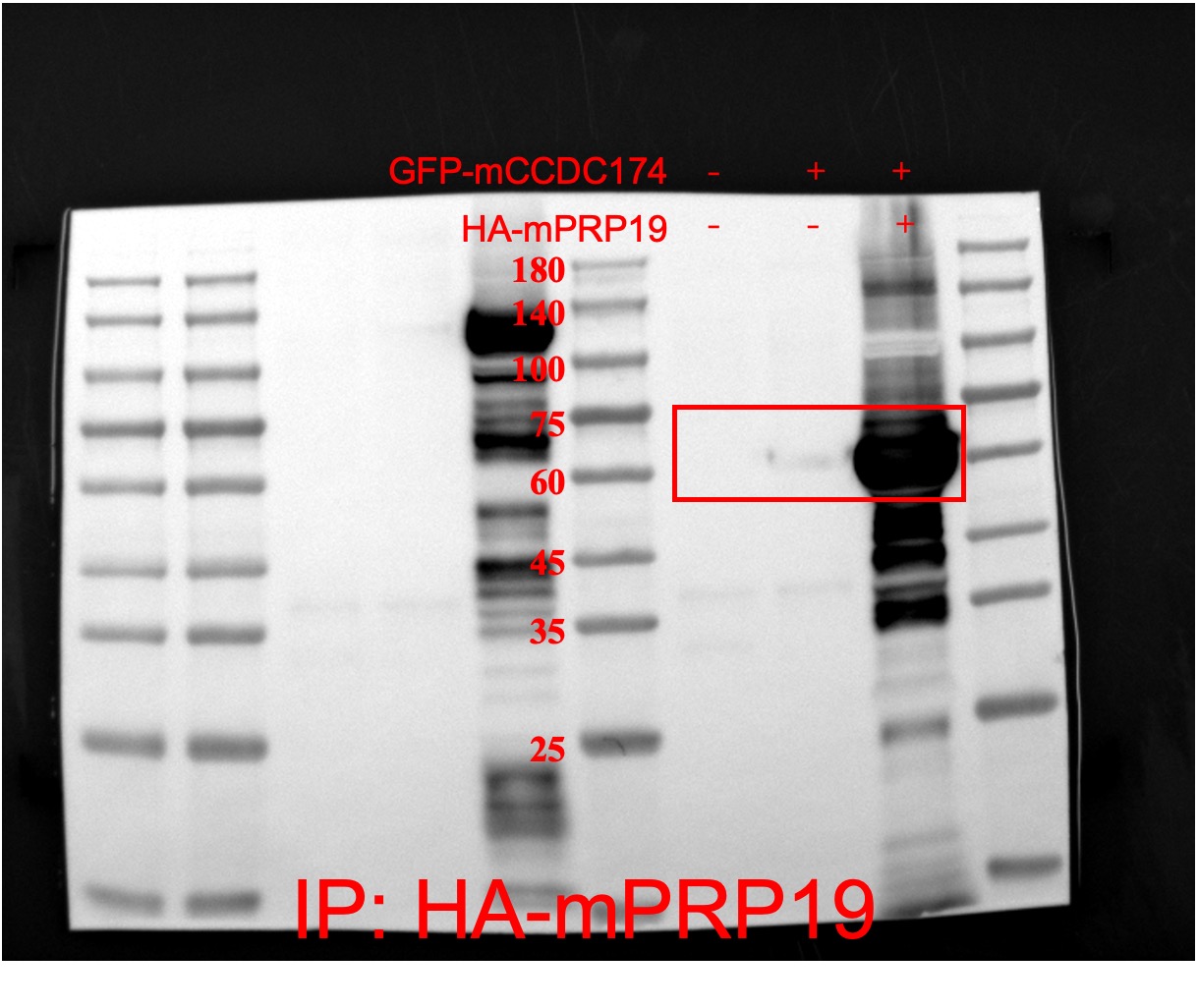

Supplement: Supplementary file 10 — Source data Fig. 6 [file 44321_2026_448_MOESM10_ESM.zip › Figure 6/6C/PRP19 IP/PRP19 IP HA-mPRP19.jpg]

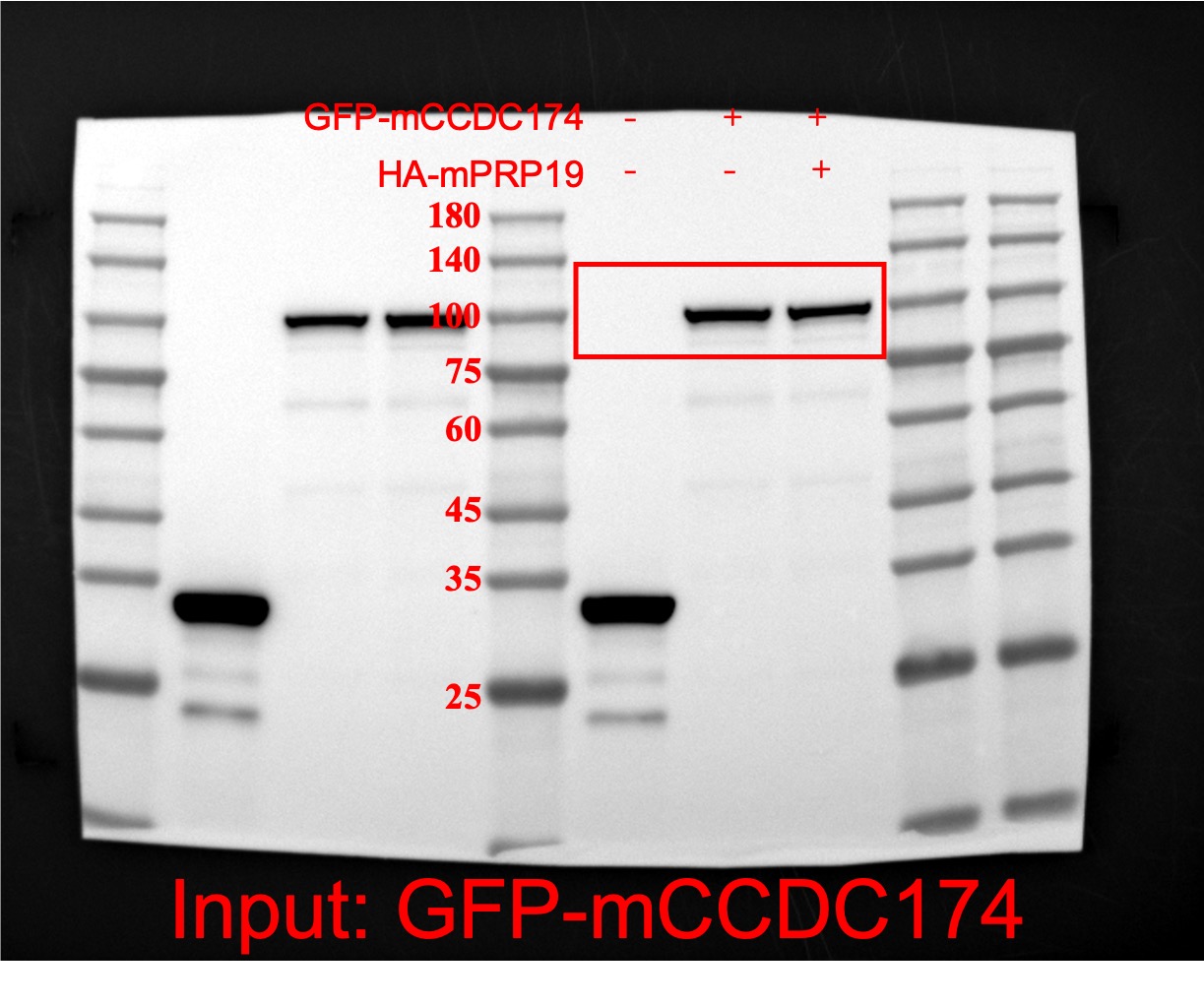

Supplement: Supplementary file 10 — Source data Fig. 6 [file 44321_2026_448_MOESM10_ESM.zip › Figure 6/6C/PRP19 IP/PRP19 Input GFP-mCCDC174.jpg]

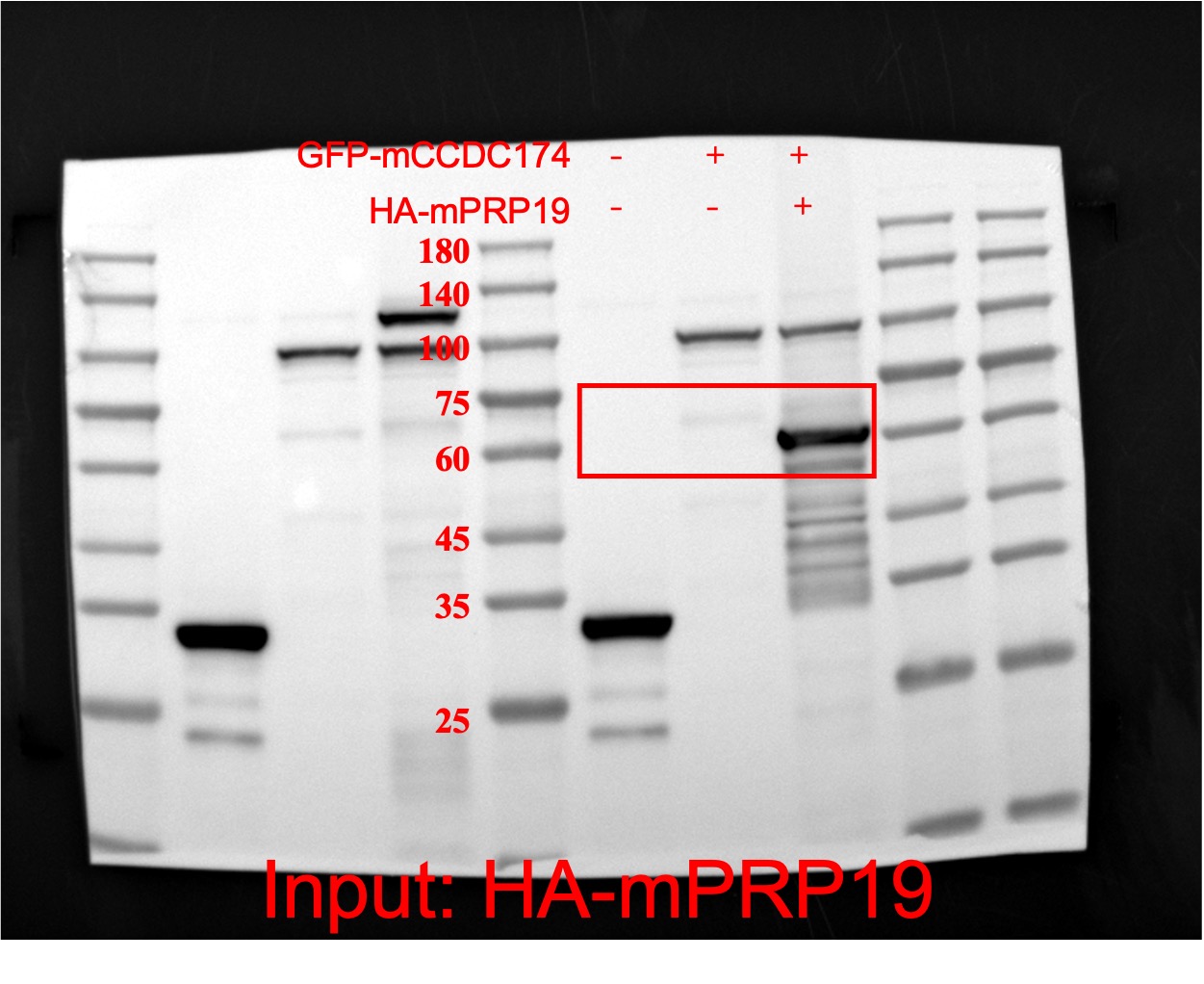

Supplement: Supplementary file 10 — Source data Fig. 6 [file 44321_2026_448_MOESM10_ESM.zip › Figure 6/6C/PRP19 IP/PRP19 Input HA-mPRP19.jpg]

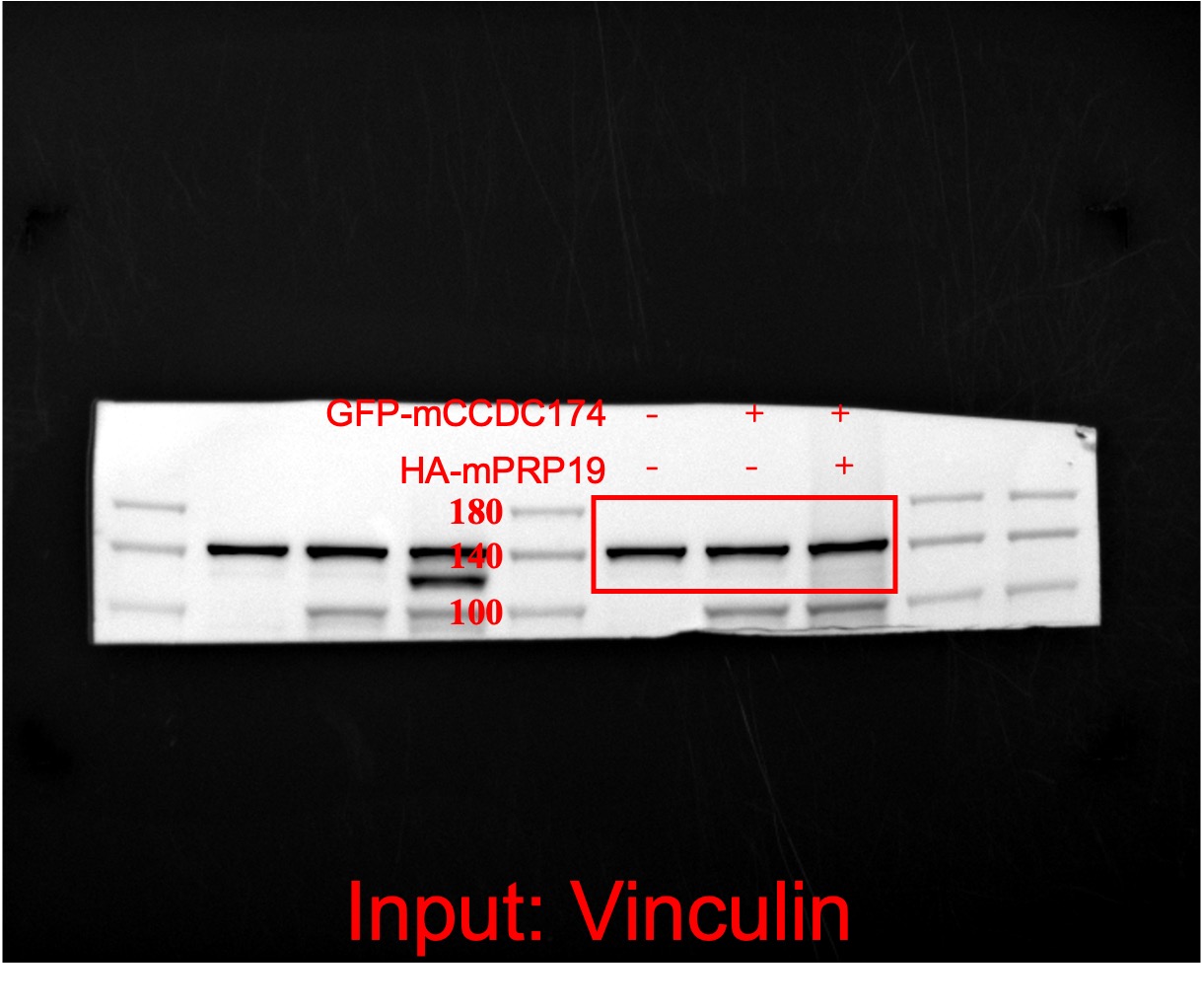

Supplement: Supplementary file 10 — Source data Fig. 6 [file 44321_2026_448_MOESM10_ESM.zip › Figure 6/6C/PRP19 IP/PRP19 Input Vinculin.jpg]

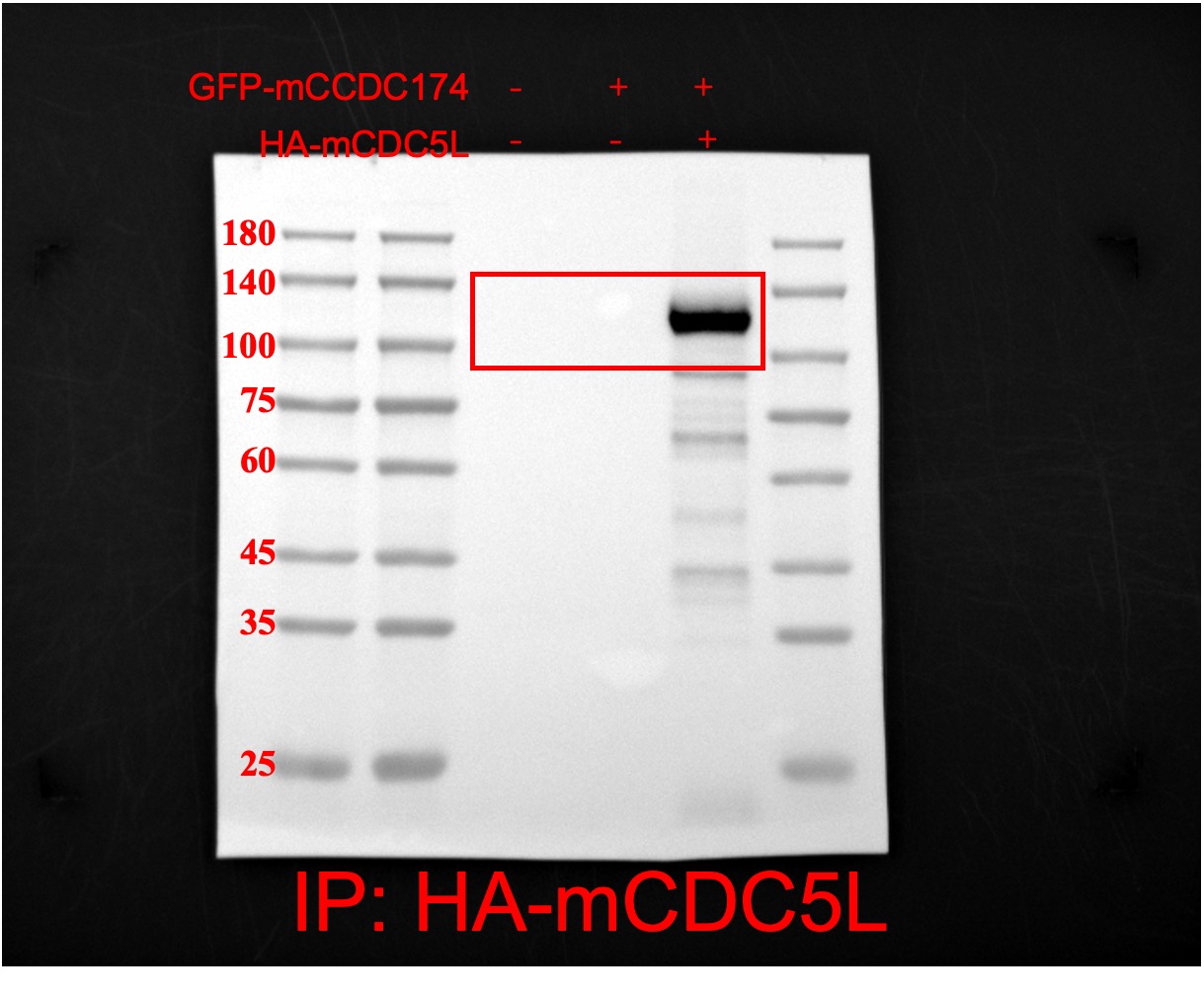

Supplement: Supplementary file 10 — Source data Fig. 6 [file 44321_2026_448_MOESM10_ESM.zip › Figure 6/6C/CDC5L IP/CDC5L IP HA-mCDC5L.jpg]

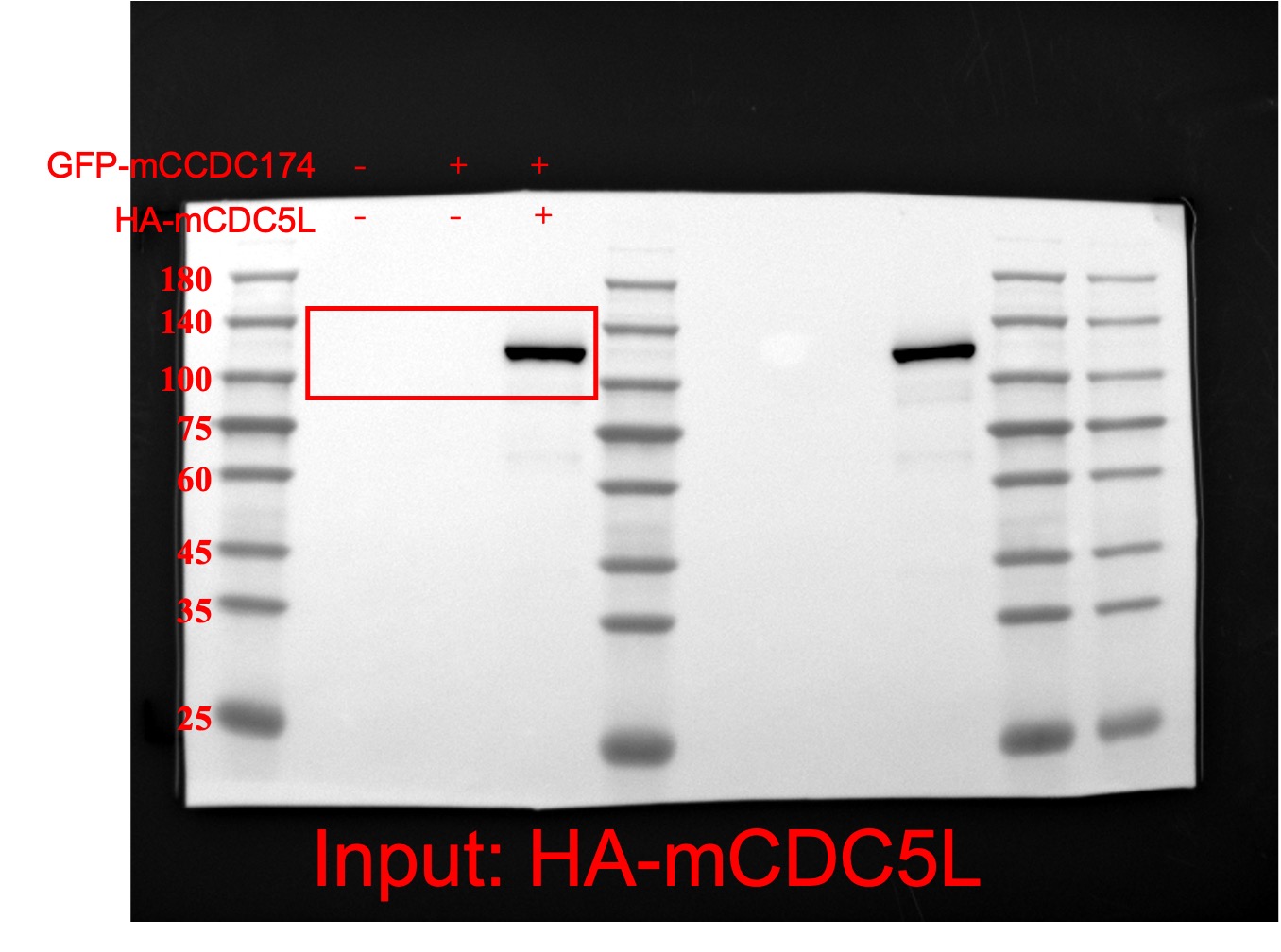

Supplement: Supplementary file 10 — Source data Fig. 6 [file 44321_2026_448_MOESM10_ESM.zip › Figure 6/6C/CDC5L IP/CDC5L Input HA-mCDC5L.jpg]

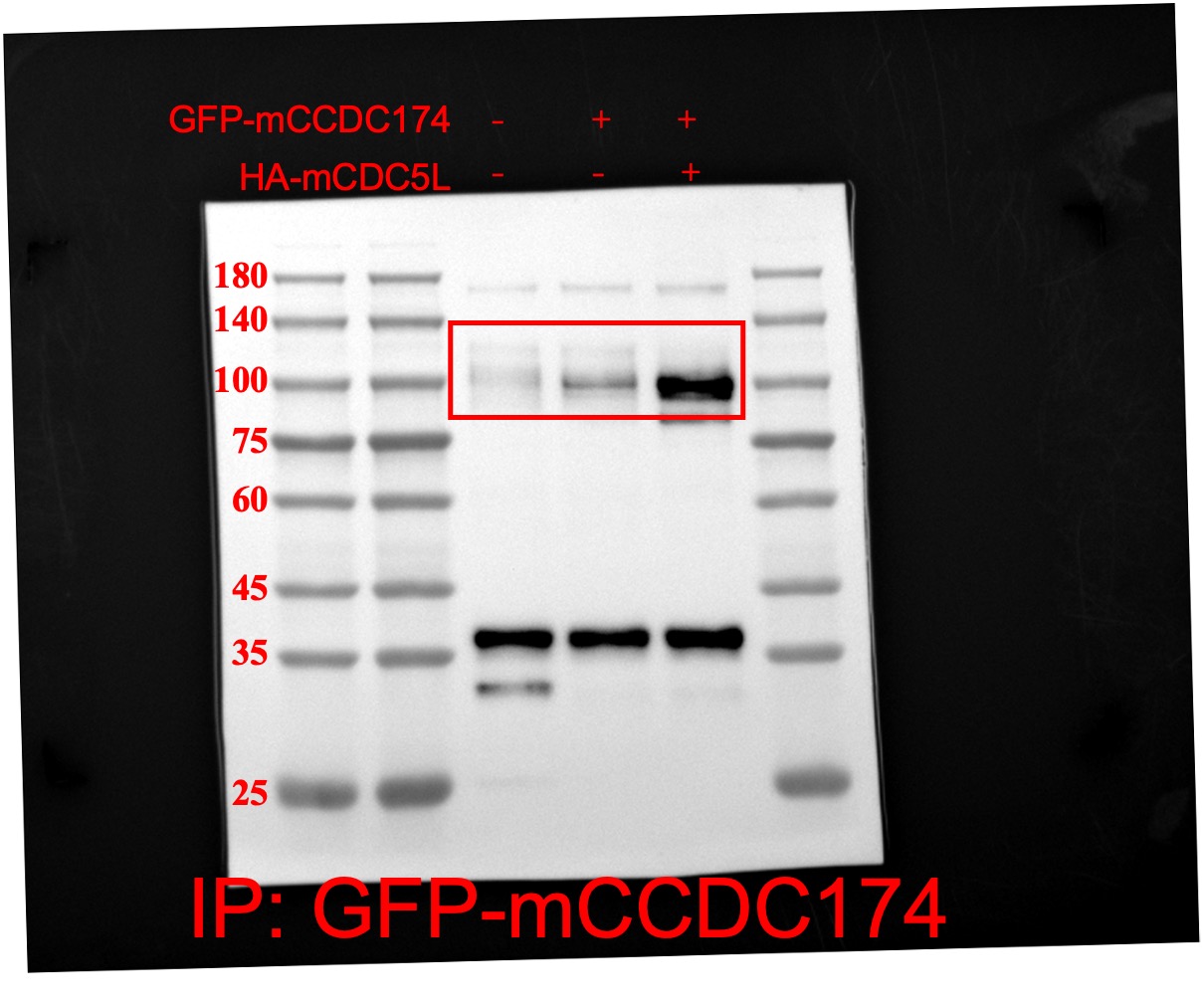

Supplement: Supplementary file 10 — Source data Fig. 6 [file 44321_2026_448_MOESM10_ESM.zip › Figure 6/6C/CDC5L IP/CDC5L IP GFP-mCCDC174.jpg]

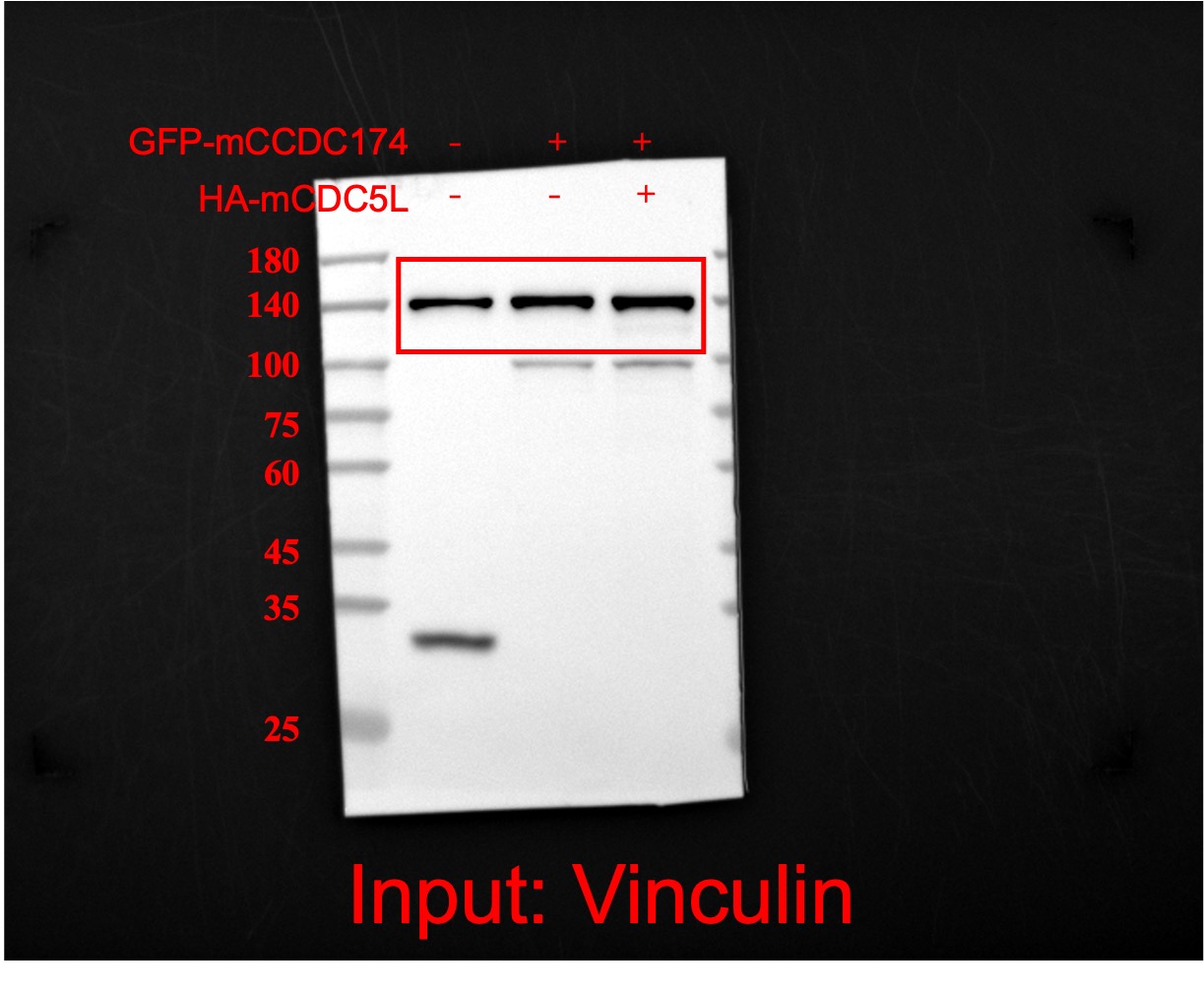

Supplement: Supplementary file 10 — Source data Fig. 6 [file 44321_2026_448_MOESM10_ESM.zip › Figure 6/6C/CDC5L IP/CDC5L Input Vinculin.jpg]

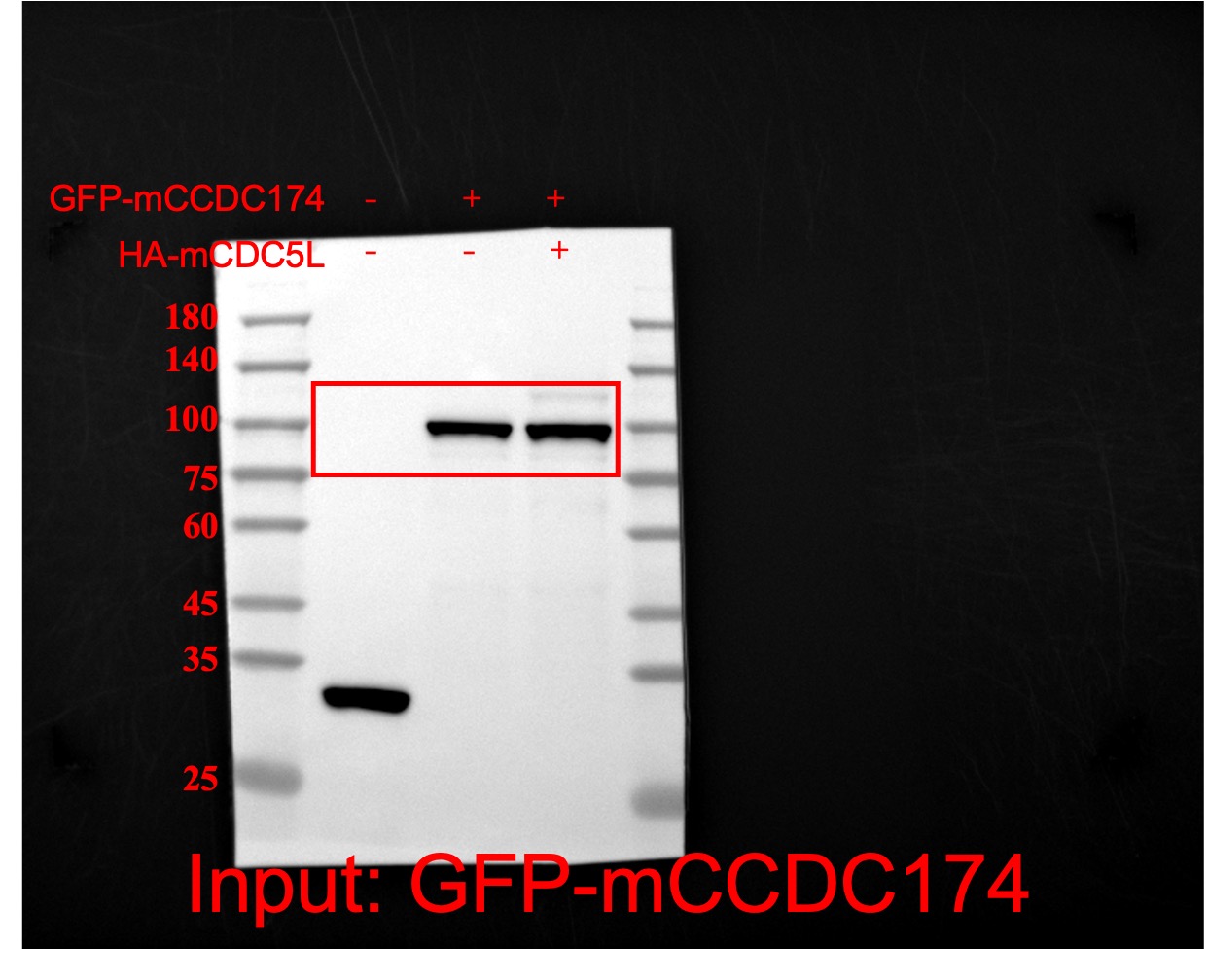

Supplement: Supplementary file 10 — Source data Fig. 6 [file 44321_2026_448_MOESM10_ESM.zip › Figure 6/6C/CDC5L IP/CDC5L Input GFP-mCCDC174.jpg]

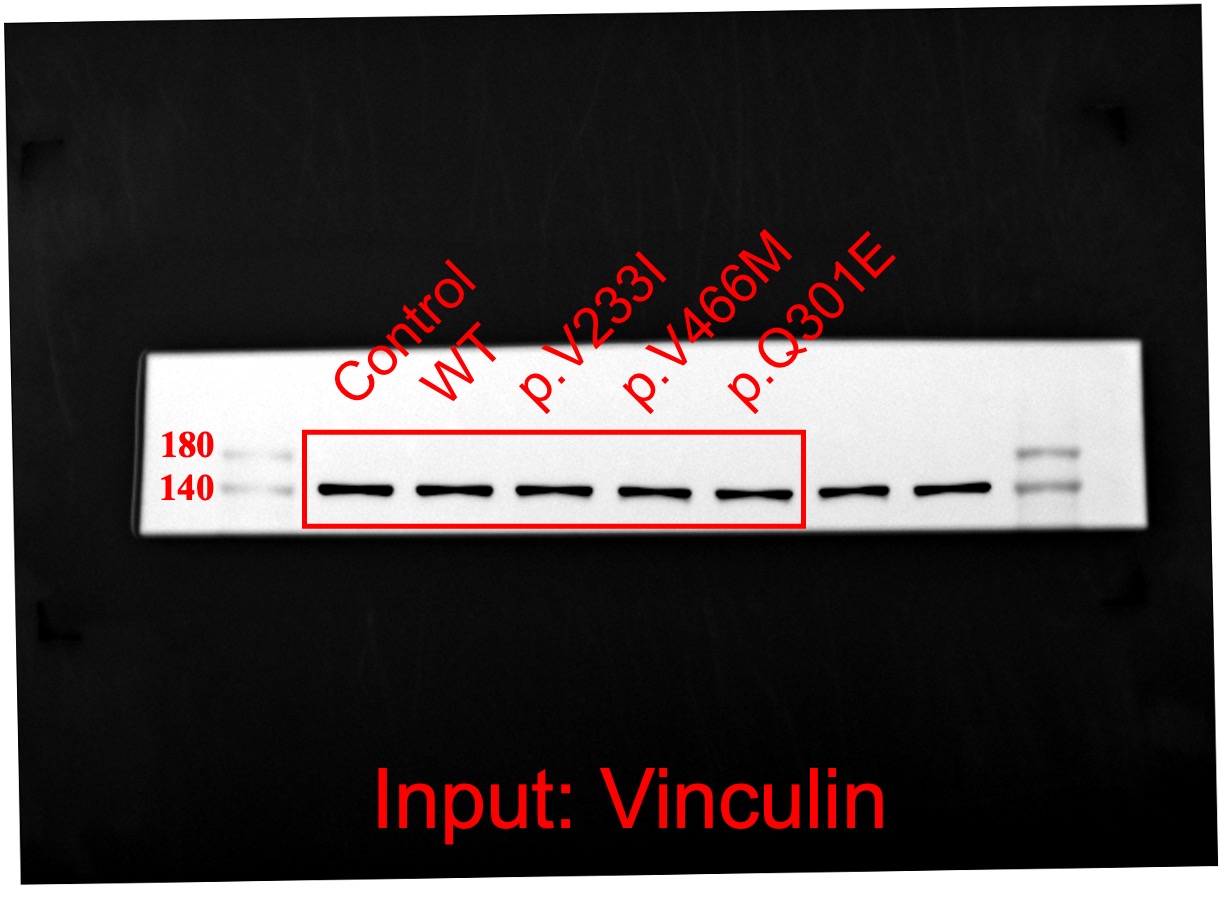

Supplement: Supplementary file 11 — Source data Fig. 7 [file 44321_2026_448_MOESM11_ESM.zip › Figure 7/7C/Input- Vinculin.jpg]

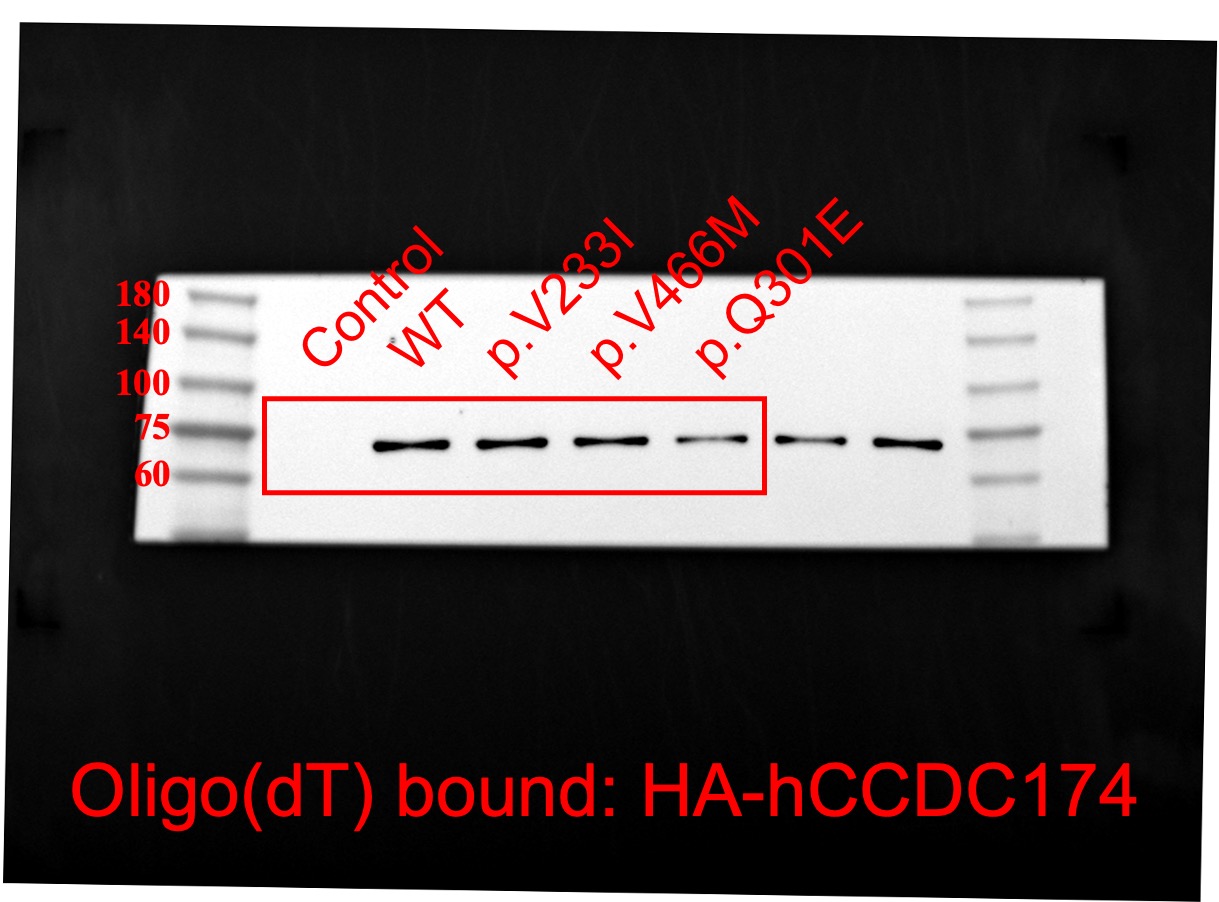

Supplement: Supplementary file 11 — Source data Fig. 7 [file 44321_2026_448_MOESM11_ESM.zip › Figure 7/7C/Oligo(dT) bound- HA-hCCDC174.jpg]

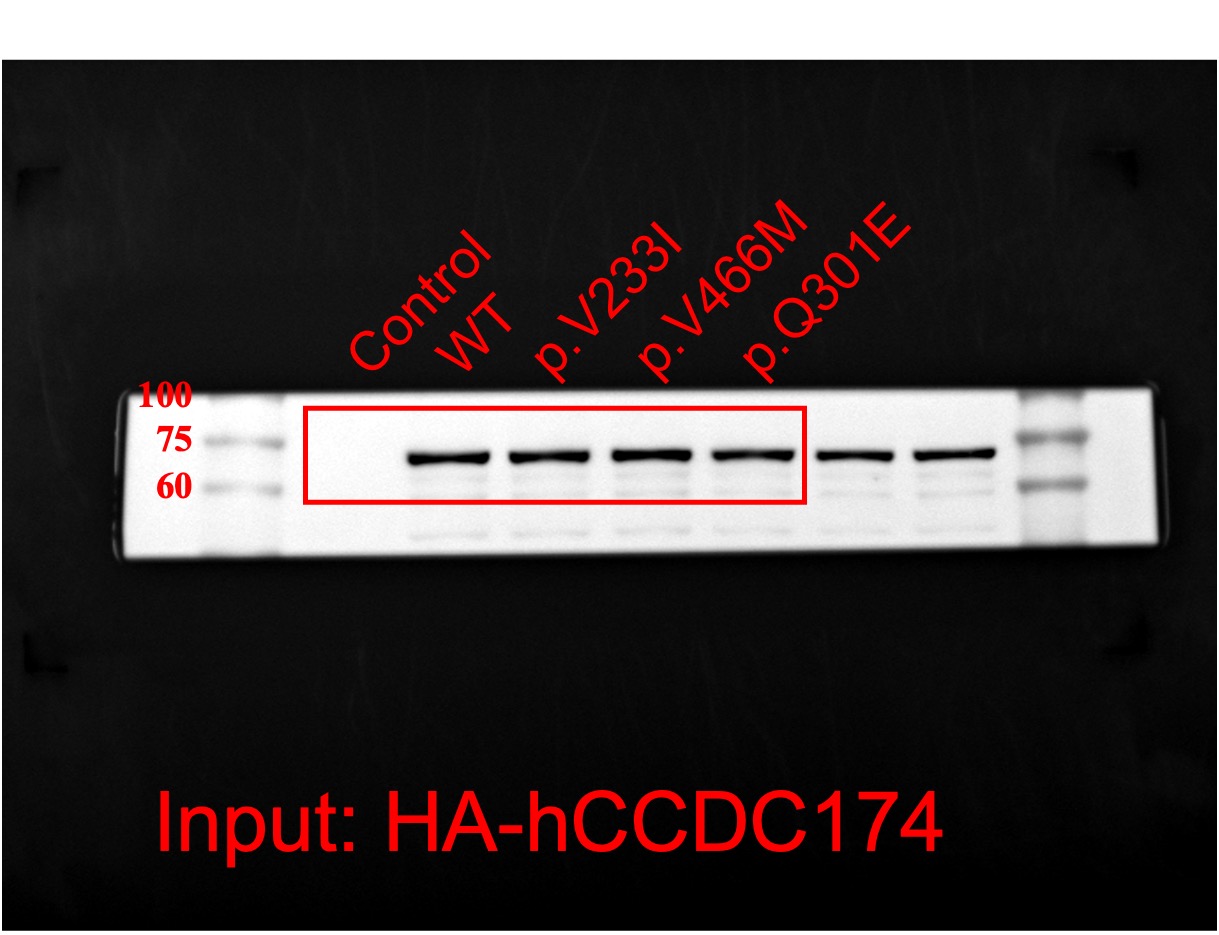

Supplement: Supplementary file 11 — Source data Fig. 7 [file 44321_2026_448_MOESM11_ESM.zip › Figure 7/7C/Input- HA-hCCDC174.jpg]

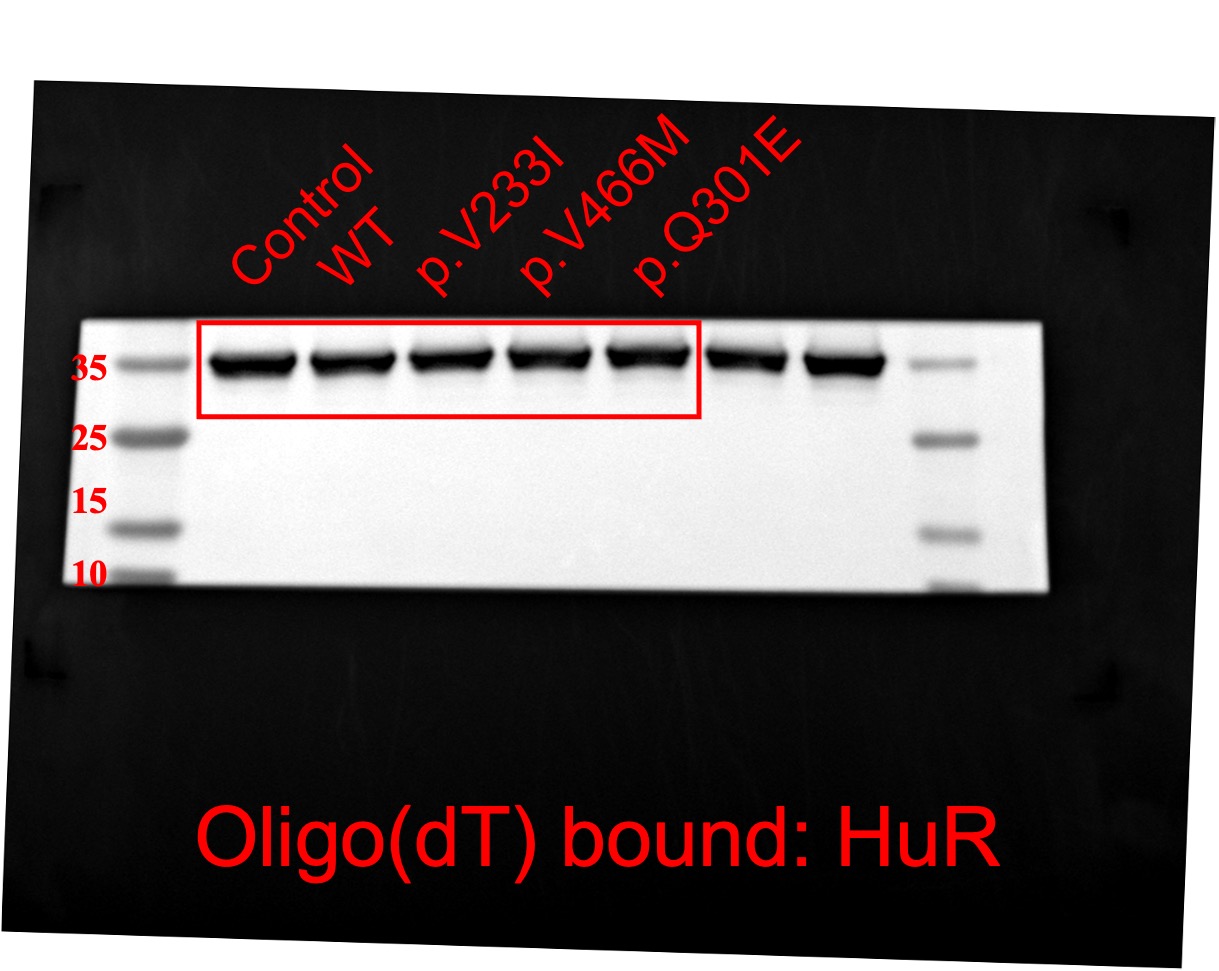

Supplement: Supplementary file 11 — Source data Fig. 7 [file 44321_2026_448_MOESM11_ESM.zip › Figure 7/7C/Oligo(dT) bound- HuR.jpg]

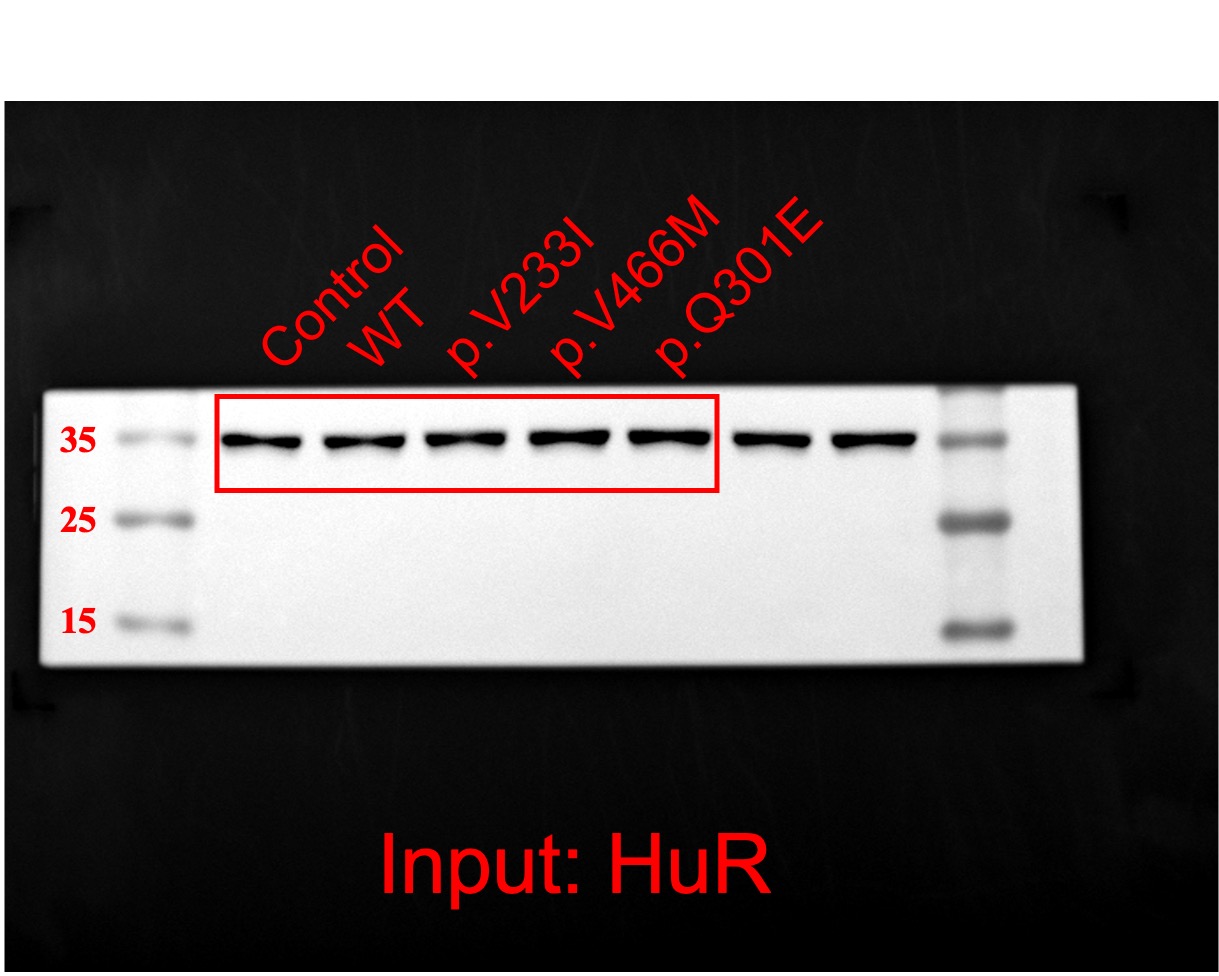

Supplement: Supplementary file 11 — Source data Fig. 7 [file 44321_2026_448_MOESM11_ESM.zip › Figure 7/7C/Input- HuR.jpg]

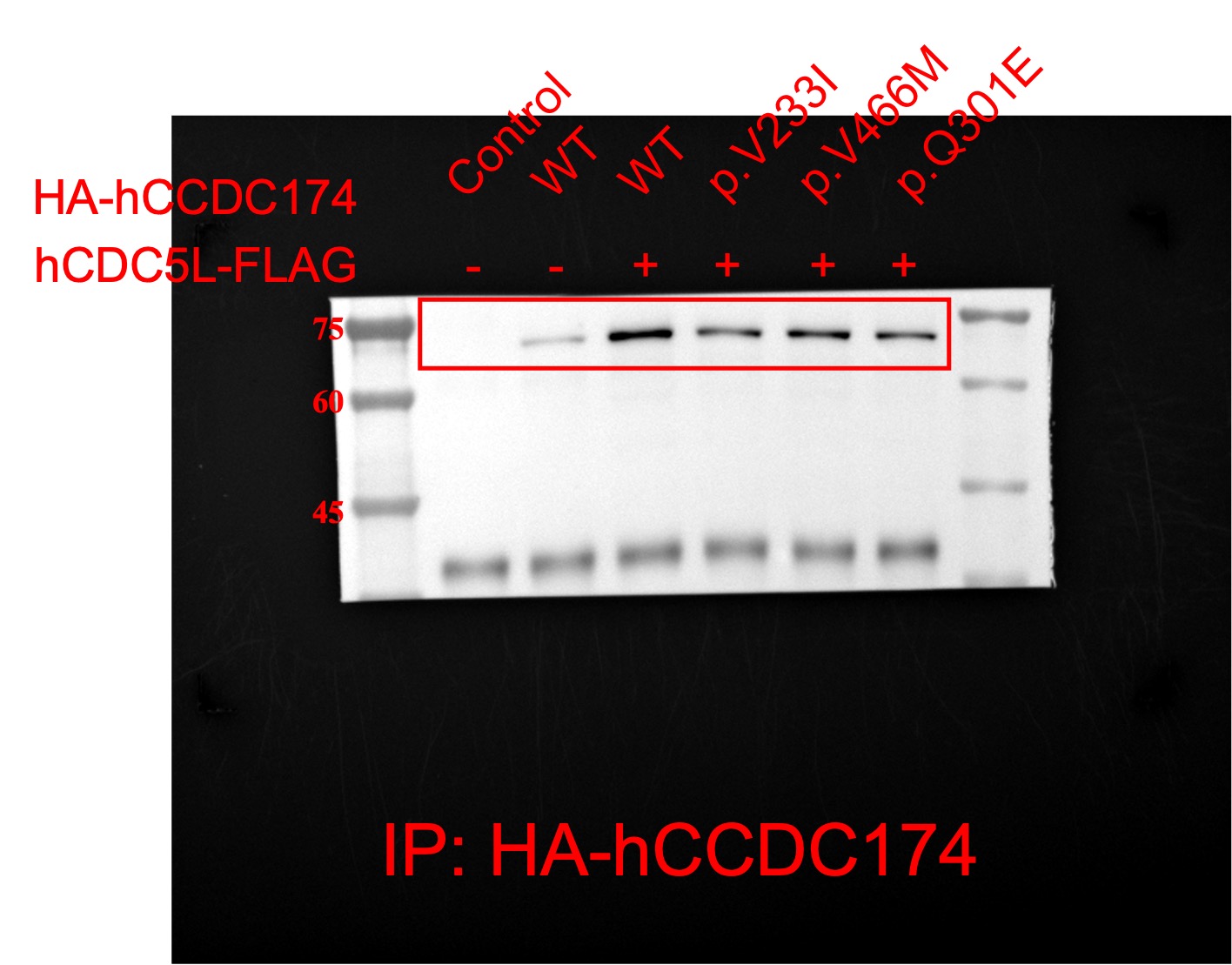

Supplement: Supplementary file 11 — Source data Fig. 7 [file 44321_2026_448_MOESM11_ESM.zip › Figure 7/7E/IP- HA-hCCDC174.jpg]

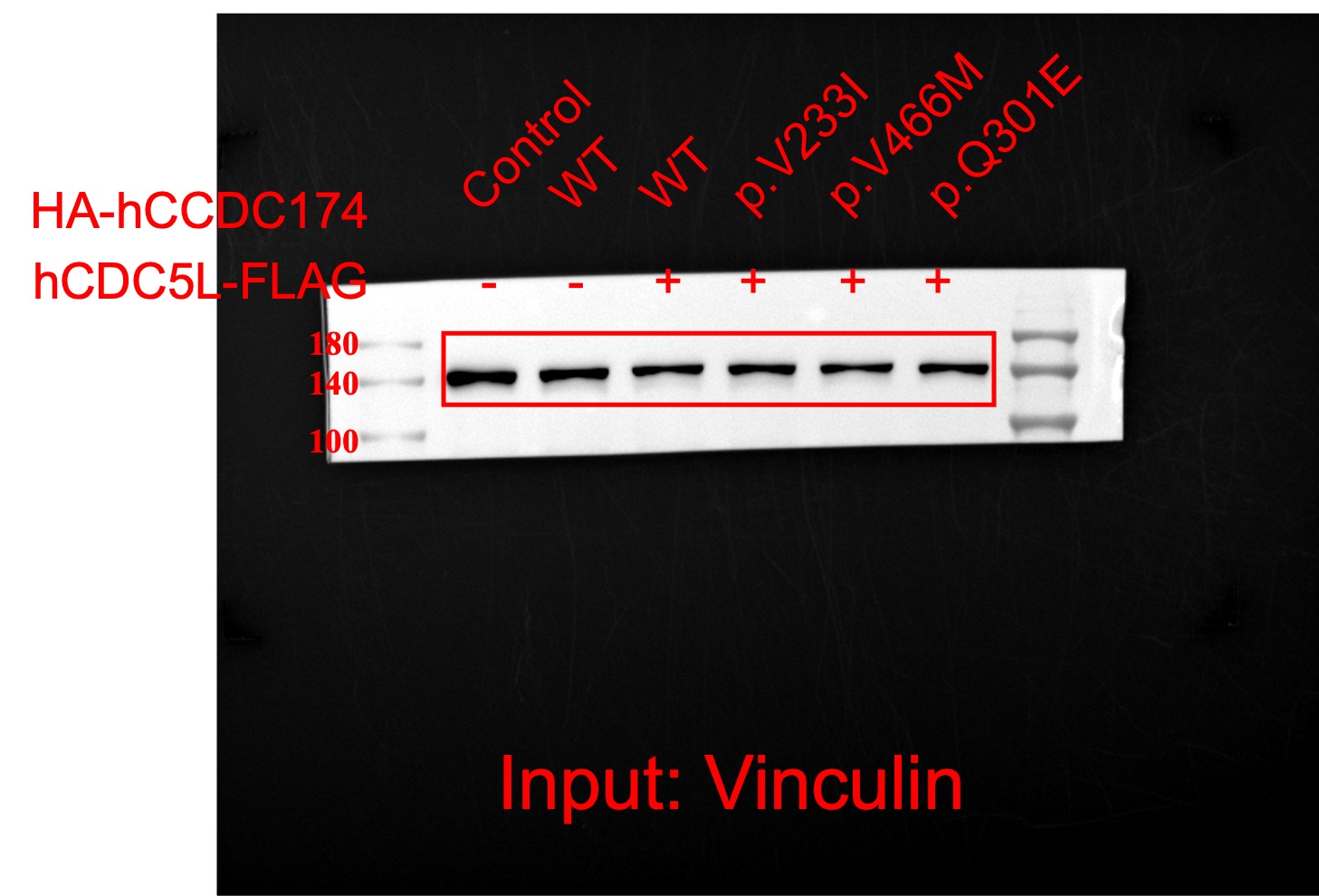

Supplement: Supplementary file 11 — Source data Fig. 7 [file 44321_2026_448_MOESM11_ESM.zip › Figure 7/7E/Input- Vinculin.jpg]

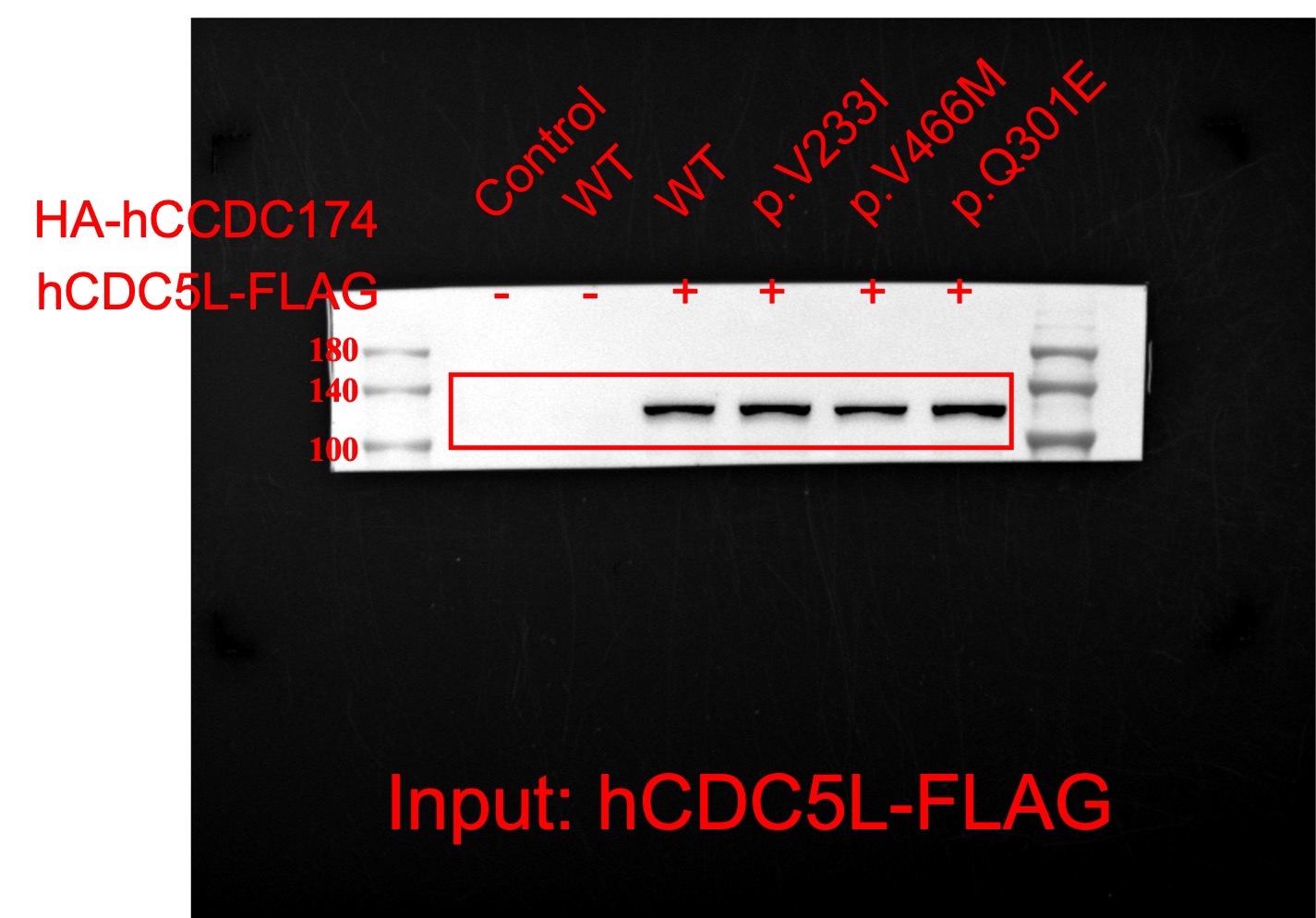

Supplement: Supplementary file 11 — Source data Fig. 7 [file 44321_2026_448_MOESM11_ESM.zip › Figure 7/7E/Input- hCDC5L-FLAG.jpg]

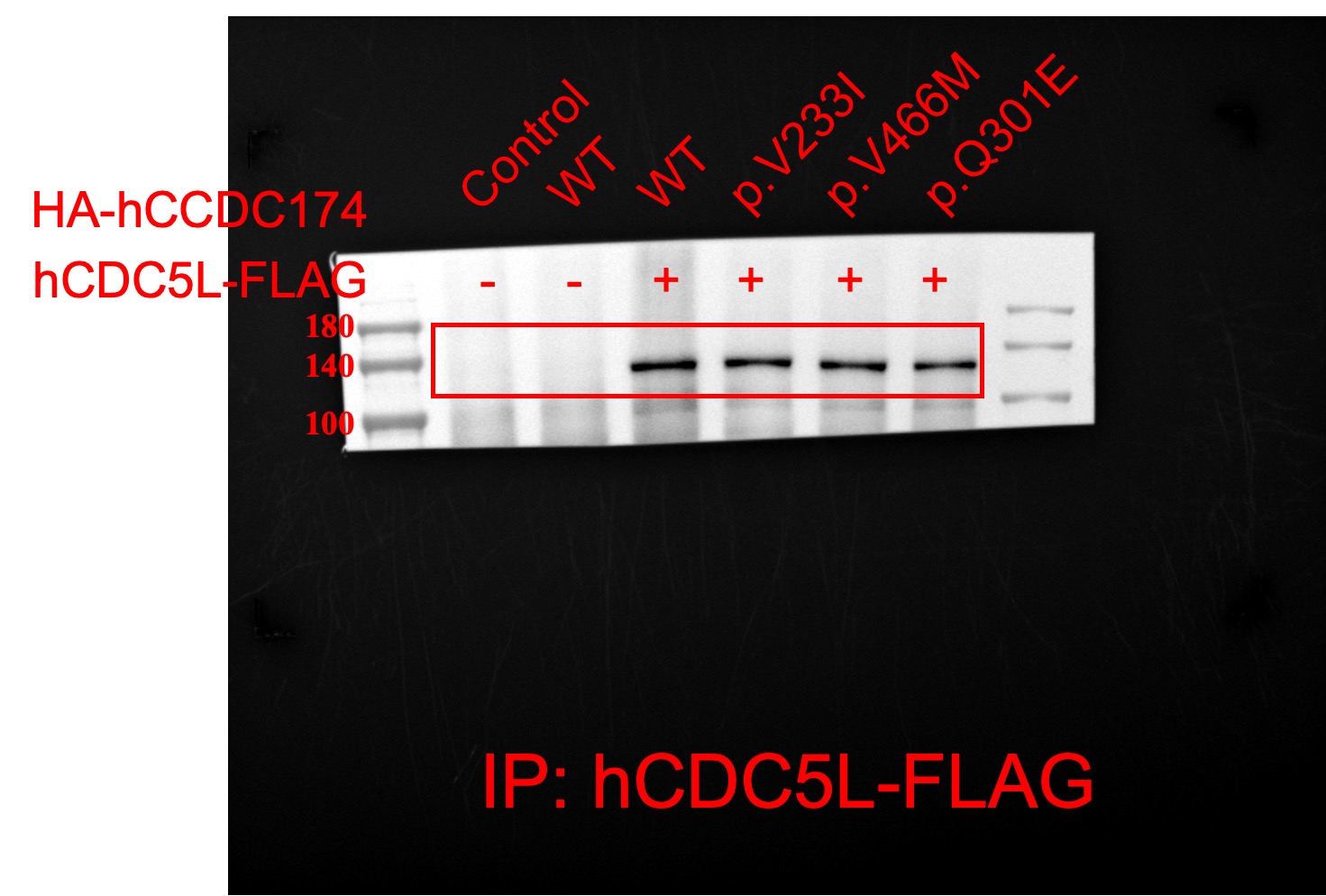

Supplement: Supplementary file 11 — Source data Fig. 7 [file 44321_2026_448_MOESM11_ESM.zip › Figure 7/7E/IP- hCDC5L-FLAG.jpg]

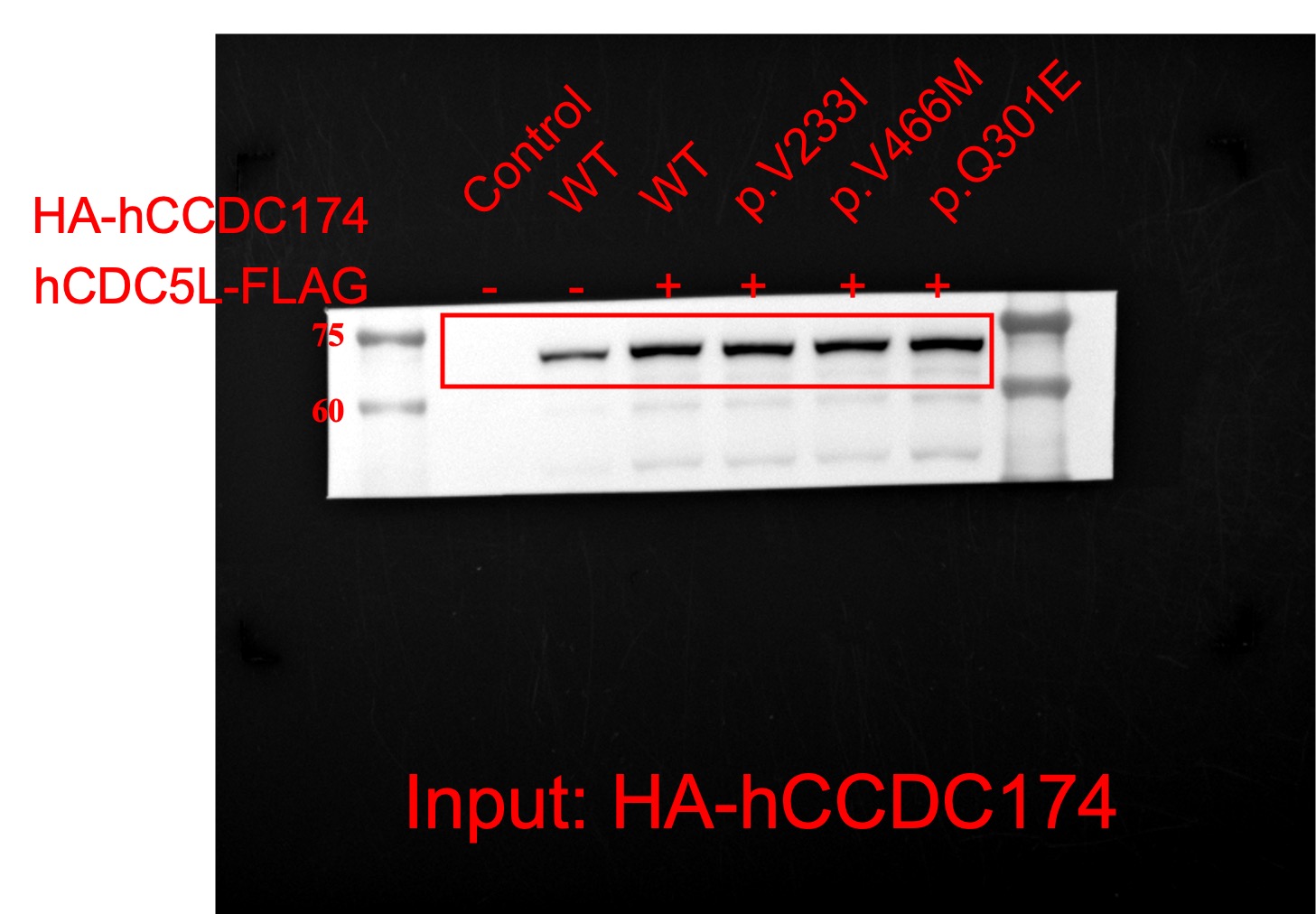

Supplement: Supplementary file 11 — Source data Fig. 7 [file 44321_2026_448_MOESM11_ESM.zip › Figure 7/7E/Input- HA-hCCDC174.jpg]

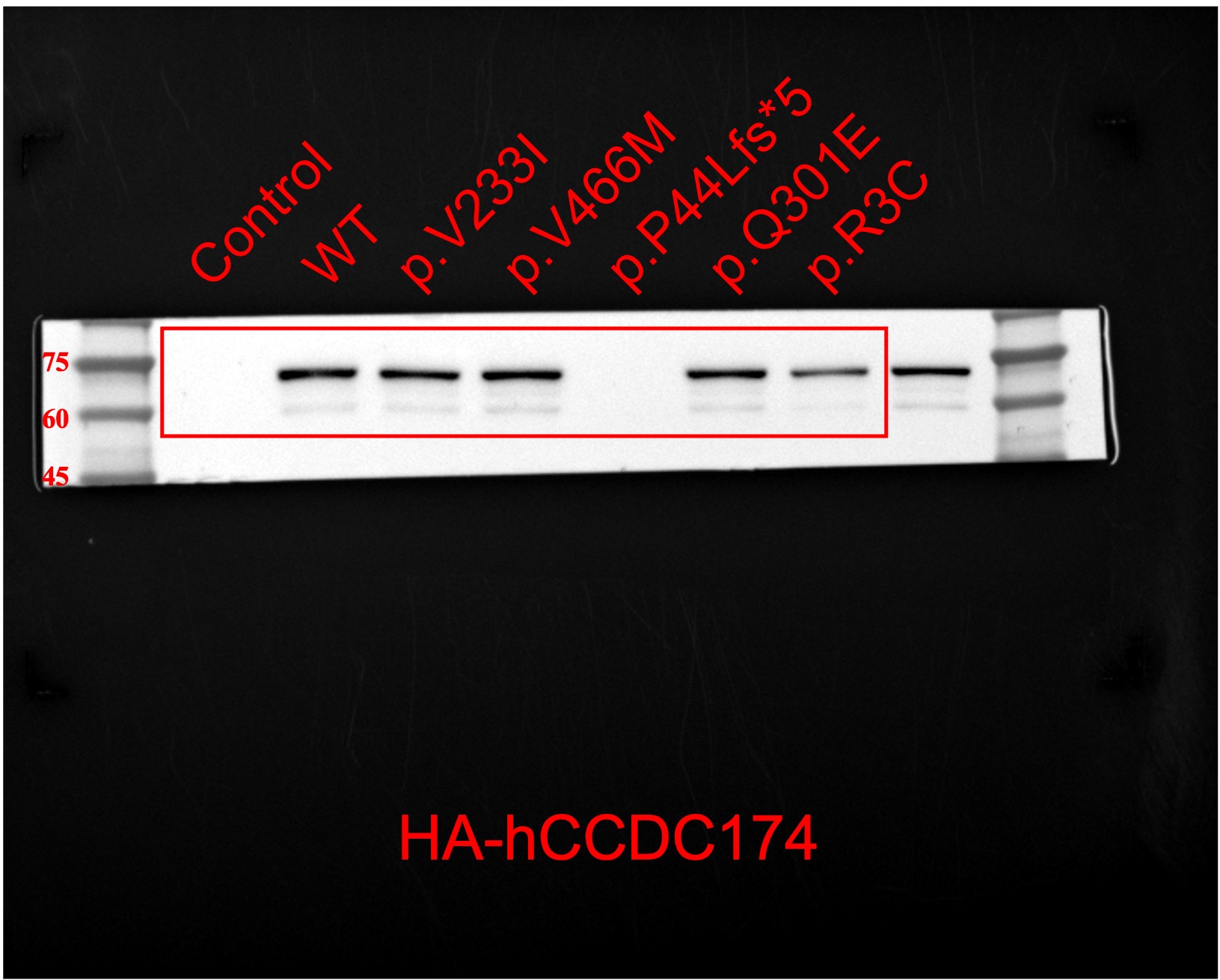

Supplement: Supplementary file 11 — Source data Fig. 7 [file 44321_2026_448_MOESM11_ESM.zip › Figure 7/7A/HA-hCCDC174.jpg]

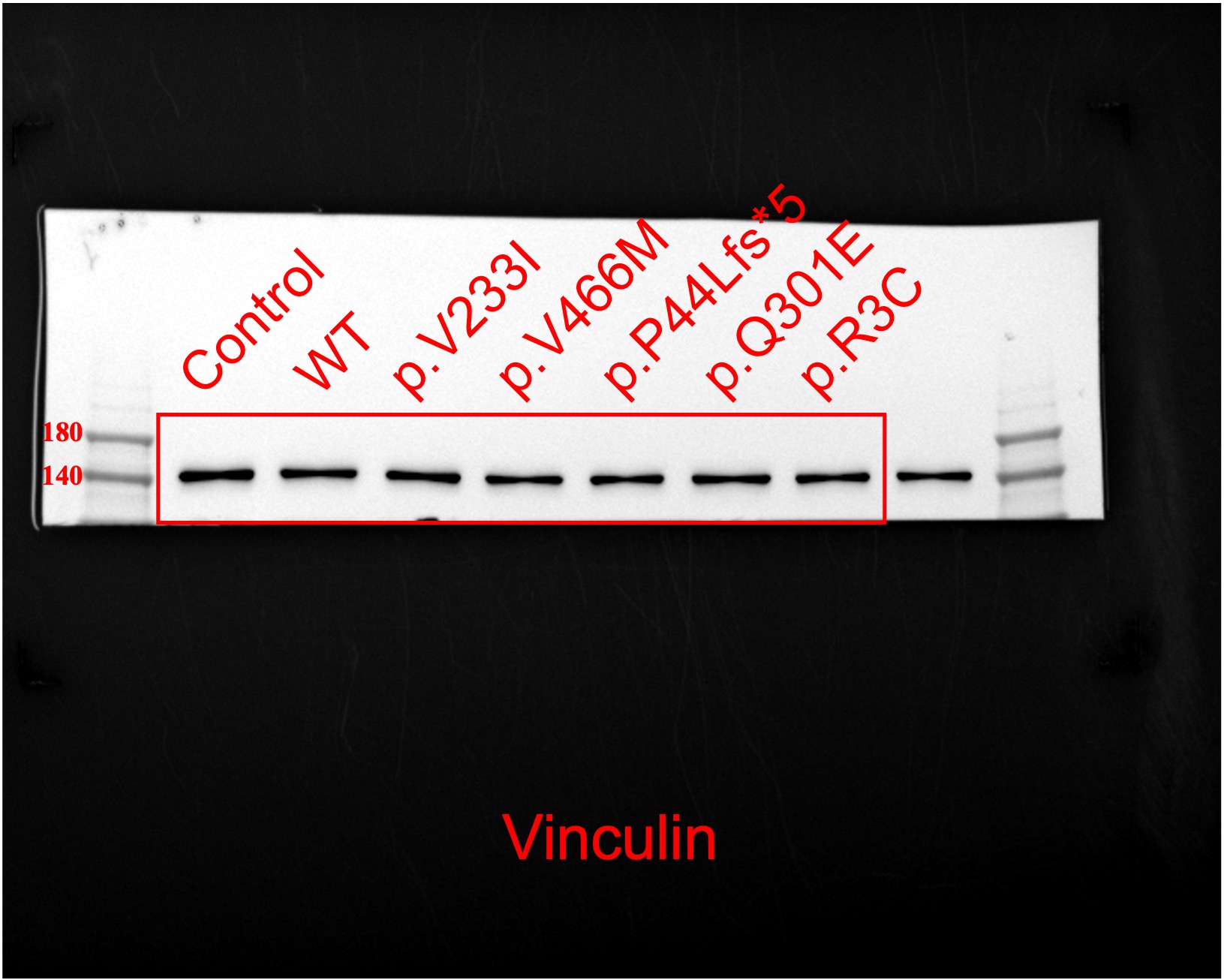

Supplement: Supplementary file 11 — Source data Fig. 7 [file 44321_2026_448_MOESM11_ESM.zip › Figure 7/7A/Vinculin.jpg]
