# Supplementary material for: CCDC174 deficiency impaired human fertility by affecting the alternative splicing of maternal mRNAs
Source: EMBO Mol Med. 2026 May 12;18(6):2436–54. doi: 10.1038/s44321-026-00448-y (PMC13270137; doi:10.1038/s44321-026-00448-y)
Supplement: Supplementary file 12 — Expanded View Figures [file 44321_2026_448_MOESM12_ESM.pdf]

Expanded View Figures

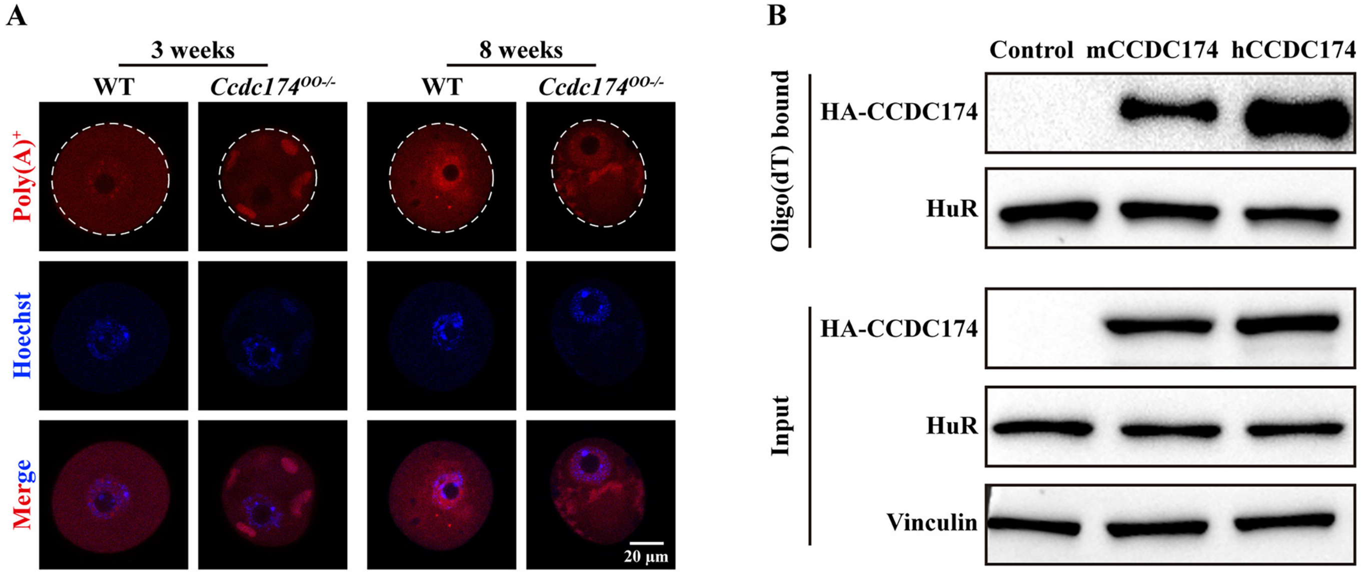

Figure EV1. CCDC174 deficiency affects maternal mRNA localization, and human and mouse CCDC174 both bind to RNAs.

(**A**) FISH of poly(A) RNAs in WT and *Ccdc174*$^{oo-/-}$ oocytes. $n \geq 13$ for each group. Dashed borders indicate the boundary of the oocyte. (**B**) Representative immunoblots of mouse and human CCDC174 bound to poly(A) RNAs in HEK293T cells. HuR was used as internal control and Vinculin was used as the loading control. $n = 3$ biological replicates.

© The Author(s)
