# Supplementary material for: CCDC174 deficiency impaired human fertility by affecting the alternative splicing of maternal mRNAs
Source: EMBO Mol Med. 2026 May 12;18(6):2436–54. doi: 10.1038/s44321-026-00448-y (PMC13270137; doi:10.1038/s44321-026-00448-y)
Supplement: Supplementary file 2 — Peer Review File [file 44321_2026_448_MOESM2_ESM.pdf]

EMBO Molecular Medicine

CCDC174 deficiency impaired human fertility by affecting the alternative splicing of maternal mRNAs

Weijie Wang, Zhiqi Pan, Huixia Jing, Rong Shi, Ling Wu, Jinjie Wang, Biaobang Chen, Jian Mu, Zhihua Zhang, Tianyu Wu, Qiaoli Li, Juanzi Shi, Yanping Kuang, Lin He, Lei Wang, and Qing Sang

Corresponding author(s): Qing Sang (sangqing@fudan.edu.cn) , Lei Wang (wangleiwanglei@fudan.edu.cn), Weijie Wang (wangweijie0816@126.com)

Review Timeline:

Submission Date:	6th Jan 26
Editorial Decision:	23rd Jan 26
Revision Received:	21st Mar 26
Editorial Decision:	21st Apr 26
Revision Received:	26th Apr 26
Accepted:	29th Apr 26

Editor: Jingyi Hou

Transaction Report:

(Note: With the exception of the correction of typographical or spelling errors that could be a source of ambiguity, letters and reports are not edited. Depending on transfer agreements, referee reports obtained elsewhere may or may not be included in this compilation. Referee reports are anonymous unless the Referee chooses to sign their reports.)

23rd Jan 2026

Dear Prof. Sang,

Thank you for submitting your work to EMBO Molecular Medicine. We have now heard back from the three reviewers who agreed to evaluate your manuscript. As you will see below, the reviewers think that the study is interesting and relevant. They raise however a series of concerns, which we would ask you to convincingly address in a revision.

The reviewers' recommendations are generally clear, so I will not repeat them in detail. Reviewer #1 requested a few additional experiments to strengthen the mechanistic insights, while Reviewers #2 and #3 suggested further analysis of mRNA splicing in patients' oocytes (if feasible), which should be addressed carefully. The concern raised by Reviewer #1 regarding the lack of a functional rescue experiment should also be addressed to make the study more conclusive, by providing at least some level of supporting evidence.

As you may already know, our editorial policy allows in principle a single round of major revision, so it is essential to provide responses to the reviewers' comments that are as complete as possible. Please feel free to contact me in case you would like to discuss in further detail any of the issues raised by the reviewers.

EMBO Molecular Medicine has a "scooping protection" policy, whereby similar findings that are published by others during review or revision are not a criterion for rejection. Should you decide to submit a revised version, I do ask that you get in touch after three months if you have not completed it, to update us on the status.

Please also contact us as soon as possible if similar work is published elsewhere. If other work is published, we may not be able to extend the revision period beyond three months.

Please read below for important editorial formatting and consult our author's guidelines for proper formatting of your revised article for EMBO Molecular Medicine.

I look forward to receiving your revised manuscript.

Sincerely,
Jingyi

Jingyi Hou
Senior Editor
EMBO Molecular Medicine

*****
When preparing your revised manuscript, please refer to our guidelines: https://link.springer.com/journal/44321/submission-guidelines#cms-Revised-submissions. We perform an initial quality control of all revised manuscripts before re-review; failure to include requested items will delay the evaluation of your revision.

We require:

1) A .docx formatted version of the manuscript text (including legends for main figures, EV figures and tables). Please make sure that the changes are highlighted to be clearly visible.

2) Individual production quality figure files as .eps, .tif, .jpg (one file per figure). For guidance, download the 'Figure Guide PDF': https://media.springernature.com/original/springer-cms/rest/v1/content/27825798/data/v1.

3) A .docx formatted letter INCLUDING the reviewers' reports and your detailed point-by-point responses to their comments. As part of the EMBO Press transparent editorial process, the point-by-point response is part of the Review Process File (RPF), which will be published alongside your paper.

4) A complete author checklist, which you can download from our author guidelines. Please insert information in the checklist that is also reflected in the manuscript. The completed author checklist will also be part of the RPF.

5) Please note that all corresponding authors are required to supply an ORCID ID for their name upon submission of a revised manuscript.

6) It is mandatory to include a 'Data Availability' section after the Materials and Methods. Before submitting your revision, primary datasets produced in this study need to be deposited in an appropriate public database, and the accession numbers and database listed under 'Data Availability'. Please remember to provide a reviewer password if the datasets are not yet public.

In case you have no data that requires deposition in a public database, please state so in this section. Note that the Data Availability Section is restricted to new primary data that are part of this study.

7) For data quantification: please specify the name of the statistical test used to generate error bars and P values, the number (n) of independent experiments (specify technical or biological replicates) underlying each data point and the test used to calculate p-values in each figure legend. The figure legends should contain a basic description of n, P and the test applied. Graphs must include a description of the bars and the error bars (s.d., s.e.m.).

8) At EMBO Press we ask authors to provide source data for the main manuscript figures. You will receive a separate email with instructions for providing source data with your revised manuscript, including how to upload and organize the files.

9) Our journal encourages inclusion of *data citations in the reference list* to directly cite datasets that were re-used and obtained from public databases. Data citations in the article text are distinct from normal bibliographical citations and should directly link to the database records from which the data can be accessed. In the main text, data citations are formatted as follows: "Data ref: Smith et al, 2001" or "Data ref: NCBI Sequence Read Archive PRJNA342805, 2017". In the Reference list, data citations must be labeled with "[DATASET]". A data reference must provide the database name, accession number/identifiers and a resolvable link to the landing page from which the data can be accessed at the end of the reference.

10) We replaced Supplementary Information with Expanded View (EV) Figures and Tables that are collapsible/expandable online. A maximum of 5 EV Figures can be typeset. EV Figures should be cited as 'Figure EV1, Figure EV2" etc... in the text and their respective legends should be included in the main text after the legends of regular figures.

- For the figures that you do NOT wish to display as Expanded View figures, they should be bundled together with their legends in a single PDF file called *Appendix*, which should start with a short Table of Content. Appendix figures should be referred to in the main text as: "Appendix Figure S1, Appendix Figure S2" etc.

- Additional Tables/Datasets should be labeled and referred to as Table EV1, Dataset EV1, etc. Legends have to be provided in a separate tab in case of .xls files. Alternatively, the legend can be supplied as a separate text file (README) and zipped together with the Table/Dataset file.

11) The paper explained: EMBO Molecular Medicine articles are accompanied by a summary of the articles to emphasize the major findings in the paper and their medical implications for the non-specialist reader. Please provide a draft summary of your article highlighting

- the medical issue you are addressing,

- the results obtained and

- their clinical impact.

This may be edited to ensure that readers understand the significance and context of the research. Please refer to any of our published articles for an example.

12) Author contributions: You will be asked to provide CRediT (Contributor Role Taxonomy) terms in the submission system. These replace a narrative author contribution section in the manuscript.

13) A Conflict of Interest statement should be provided in the main text.

14) Every published paper includes a 'Synopsis' to further enhance discoverability. Synopses are displayed on the journal webpage and are freely accessible to all readers. They include a short stand first (maximum of 300 characters, including space) as well as 2-5 one-sentences bullet points that summarizes the paper. Please write the bullet points to summarize the key NEW findings. They should be designed to be complementary to the abstract - i.e. not repeat the same text. We encourage inclusion of key acronyms and quantitative information (maximum of 30 words / bullet point). Please use the passive voice. Please attach these in a separate file or send them by email, we will incorporate them accordingly.

Please provide a visual abstract to illustrate your article as a PNG file 550 px wide x 300-600 px high.

15) Please download and fill our Reagents and Tools Table template (.docx), which you can find in our author guidelines: https://link.springer.com/journal/44320/submission-guidelines#structuredmethods.

When submitting your revised manuscript, please do not include the Reagents and Tools Table in the Methods section of the manuscript but upload it as a separate file choosing the file type "Reagent Table".

EMBO Molecular Medicine has a "scooping protection" policy, whereby similar findings that are published by others during review or revision are not a criterion for rejection. Should you decide to submit a revised version, I do ask that you get in touch after three months if you have not completed it, to update us on the status.

***** Reviewer's comments *****

Referee #1 (Comments on Novelty/Model System for Author):

See below, including the necessary additional experiments.

Referee #1 (Remarks for Author):

The manuscript of Weijie Wang et al, identifies biallelic variants in CCDC174 as a cause of human oocyte competence defects and female infertility. Using human genetics, an oocyte-specific knockout mouse model, multi-omics analyses, and biochemical assays, the authors propose that CCDC174 acts as a novel regulator of alternative splicing (AS) of maternal mRNAs through interaction with the PRP19/CDC5L spliceosomal complex.
The study addresses a clinically and biologically important problem, namely unexplained oocyte competence defects underlying female infertility. The identification of new maternal-effect genes has clear diagnostic implications.
To date, no human infertility disorder has been attributed to mutations in genes encoding alternative splicing regulators acting in oocytes. CCDC174 has not previously been implicated in human infertility. The study identifies CCDC174 as a novel upstream regulator of the PRP19/CDC5L complex in oocytes
The mechanistic link to the PRP19/CDC5L spliceosomal complex is biologically plausible and well supported by the interaction data.
Importantly, there is strong precedent in rodents for a reproductive role of this complex. Oocyte-specific deletion of Bcas2, a core PRP19/CDC5L component, causes severe alternative splicing defects of maternal mRNAs and complete female infertility in mice. By contrast, direct human infertility phenotypes associated with PRP19/CDC5L core components have not been reported, most likely because these genes are essential for general cell viability and embryonic development.
In this context, the identification of CCDC174 as a tissue-specific upstream regulator of the PRP19/CDC5L complex provides an elegant explanation for how perturbation of spliceosome regulation can result in isolated female infertility rather than systemic developmental disease. This represents an important conceptual contribution of the study.

Experimental quality
The experimental strategy is comprehensive and well executed, combining:
• robust human genetic evidence (large case-control cohort),
• a conditional oocyte-specific knockout mouse model,
• transcriptomic, proteomic, and AS analyses in oocytes,
• biochemical validation of RNA binding and protein-protein interactions,
• functional characterization of patient-derived variants.
Overall data quality is high, and the main conclusions are supported by the results

Mechanistic insight
The interaction between CCDC174 and the PRP19/CDC5L spliceosomal complex is convincingly demonstrated. The extensive AS defects observed in knockout oocytes are biologically plausible and relevant. However, the precise molecular role of CCDC174 (e.g. scaffold vs regulator of spliceosome activity) remains partially unresolved.

Relevance to infertility types
The study clearly establishes a role for CCDC174 in female infertility. There is no evidence for involvement in male infertility, which should be explicitly stated.
The manuscript provides a conceptual advance by establishing, for the first time, a direct link between a splicing regulator and human female reproductive disease. It complements and extends recent studies on BCAS2, ESRP1, and other post-transcriptional regulators of maternal mRNA homeostasis.

Weaknesses and missing experiments
The main limitation is the lack of functional rescue experiments. In particular :

1. Lack of in vivo functional rescue experiments demonstrating that reintroduction of wild-type CCDC174 can restore oocyte maturation in knockout oocytes.
2. The connection between specific alternative splicing defects and the observed cellular phenotypes remains largely correlative. The link could be obtained through identification and validation of at least one or two key target genes (e.g. involved in cell cycle regulation, DNA repair, or oocyte maturation) whose aberrant splicing is likely to account for the observed defects
3. Some mechanistic interpretations (e.g. compensatory upregulation of PRP19/CDC5L complex components) are plausible but not directly tested.

These additions would significantly enhance the mechanistic depth expected for EMBO Mol Med.

Referee #2 (Comments on Novelty/Model System for Author):

NONE

Referee #2 (Remarks for Author):

Oocyte competence defect is a common cause for IVF/ICSI failure, but the underlying mechanisms are largely unknown. In this work, Wang et al. identified homozygous or compound heterozygous variants in CCDC174 in five patients with oocyte competence defects. In vivo results using Ccdc174 knockout mouse showed that deletion of CCDC174 led to oocyte maturation arrest and infertility in female mice. They then used a series of experiments, including transcriptomic, proteomic, and IP-MS analyses, to find that CCDC174 regulates the alternative splicing of maternal mRNA through its interaction with the PRP19/CDC5L complex. Finally, they demonstrated that patient-derived variants are disruptive to protein function, including expression and binding to RNAs or CDC5L. This is the first study to reveal the critical role of CCDC174 in human reproduction. This study not only provides new insights into the molecular mechanism of oocyte quality control, but also provide a new diagnostic marker for clinical genetic counseling, interesting and highly innovative.
I have a few concerns as follows:
1. In the second paragraph of the Introduction, the authors first highlight the importance of maternal mRNA homeostasis for oocyte competence by introducing six reported mutant genes that regulate maternal mRNA metabolism. They then describe the important role of alternative splicing in regulating maternal mRNA homeostasis. It is important to note that the evidence for alternative splicing regulating maternal mRNA homeostasis is derived entirely from mice.
2. CCDC174 deficiency leads to abnormal mRNA splicing in mouse oocytes. If possible, it is recommended that the authors evaluate mRNA splicing in oocytes from patients with CCDC174 variants.
3. For patients carrying the CCDC174 variants, what possible treatment strategies do the authors know that could improve their fertility in the future?
4, Patients with CCDC174 variants show phenotype of oocyte maturation, fertilization and embryonic defects. However, Ccdc174 knockout mouse show phenotype of oocyte GV arrest, please discuss the phenotypic difference. In addition, please also discuss how Ccdc174 dysfunction resulted in oocyte GV arrest.
5, Does CCDC174 also regulate AS in somatic cells, if yes, whether the patient have phenotypes in other somatic tissues?

Referee #3 (Comments on Novelty/Model System for Author):

In this study, the authors identified homozygous or compound heterozygous mutations in CCDC174 in five infertile patients presenting with oocyte competence defects. To date, the understanding of CCDC174's function is very limited, and its role specifically in reproduction remains completely unknown. Oocyte-specific knockout mice confirmed in vivo that this gene is essential for oocyte development and female fertility. Through multi-omics analysis and diverse biochemical/molecular experiments, the authors revealed that CCDC174 regulates the AS and homeostasis of maternal mRNAs in oocytes via interaction with the PRP19/CDC5L complex. In vitro functional experiments demonstrated that patient-derived mutations impair the expression of CCDC174 or its binding ability to RNA/CDC5L.
This is an interesting and comprehensive study that is supported by both in vitro and in vivo experiments. The manuscript is well-written, and all experiments and results clearly described. This work is the first to report the physiological and pathological mechanisms of CCDC174 in oocyte development, providing novel diagnostic markers for female infertility patients with oocyte competence defects.

Referee #3 (Remarks for Author):

In this study, the authors identified homozygous or compound heterozygous mutations in CCDC174 in five infertile patients presenting with oocyte competence defects. To date, the understanding of CCDC174's function is very limited, and its role

specifically in reproduction remains completely unknown. Oocyte-specific knockout mice confirmed in vivo that this gene is essential for oocyte development and female fertility. Through multi-omics analysis and diverse biochemical/molecular experiments, the authors revealed that CCDC174 regulates the AS and homeostasis of maternal mRNAs in oocytes via interaction with the PRP19/CDC5L complex. In vitro functional experiments demonstrated that patient-derived mutations impair the expression of CCDC174 or its binding ability to RNA/CDC5L.

This is an interesting and comprehensive study that is supported by both in vitro and in vivo experiments. The manuscript is well-written, and all experiments and results clearly described. This work is the first to report the physiological and pathological mechanisms of CCDC174 in oocyte development, providing novel diagnostic markers for female infertility patients with oocyte competence defects.

I have the following suggestions.

1. The oocytes of all five patients exhibited low developmental competence, which was specifically manifested as oocyte maturation arrest, fertilization failure, and embryonic arrest. What might be the possible reasons for the phenotypic heterogeneity among different patients?
2. In Figure 2A, the RNA level of Ccdc174 in mouse zygotes is also relatively high. Is this gene indispensable for early embryonic development, and will downregulating this gene in zygotes affect the early embryonic development of mice?
3. In this study, why did the authors directly construct an oocyte-specific knockout mouse model instead of a global knockout mouse model? What is the phenotype of the knockout (KO) mouse? These key points need to be addressed by the authors in the manuscript.
4. If available, I suggest to detect the splicing events in patient oocytes.

***** Reviewer's comments *****

Referee #1 (Comments on Novelty/Model System for Author):
See below, including the necessary additional experiments.
Referee #1 (Remarks for Author):

The manuscript of Weijie Wang et al, identifies biallelic variants in CCDC174 as a cause of human oocyte competence defects and female infertility. Using human genetics, an oocyte-specific knockout mouse model, multi-omics analyses, and biochemical assays, the authors propose that CCDC174 acts as a novel regulator of alternative splicing (AS) of maternal mRNAs through interaction with the PRP19/CDC5L spliceosomal complex.
The study addresses a clinically and biologically important problem, namely unexplained oocyte competence defects underlying female infertility. The identification of new maternal-effect genes has clear diagnostic implications.
To date, no human infertility disorder has been attributed to mutations in genes encoding alternative splicing regulators acting in oocytes. CCDC174 has not previously been implicated in human infertility. The study identifies CCDC174 as a novel upstream regulator of the PRP19/CDC5L complex in oocytes
The mechanistic link to the PRP19/CDC5L spliceosomal complex is biologically plausible and well supported by the interaction data.
Importantly, there is strong precedent in rodents for a reproductive role of this complex. Oocyte-specific deletion of Bcas2, a core PRP19/CDC5L component, causes severe alternative splicing defects of maternal mRNAs and complete female infertility in mice. By contrast, direct human infertility phenotypes associated with PRP19/CDC5L core components have not been reported, most likely because these genes are essential for general cell viability and embryonic development.
In this context, the identification of CCDC174 as a tissue-specific upstream regulator of the PRP19/CDC5L complex provides an elegant explanation for how perturbation of spliceosome regulation can result in isolated female infertility rather than systemic developmental disease. This represents an important conceptual contribution of the study.
Experimental quality
The experimental strategy is comprehensive and well executed, combining:
• robust human genetic evidence (large case-control cohort),
• a conditional oocyte-specific knockout mouse model,
• transcriptomic, proteomic, and AS analyses in oocytes,
• biochemical validation of RNA binding and protein-protein interactions,
• functional characterization of patient-derived variants.

Overall data quality is high, and the main conclusions are supported by the results

Response: Thanks for your positive feedback and thoughtful comments.

Mechanistic insight

The interaction between CCDC174 and the PRP19/CDC5L spliceosomal complex is convincingly demonstrated. The extensive AS defects observed in knockout oocytes are biologically plausible and relevant. However, the precise molecular role of CCDC174 (e.g. scaffold vs regulator of spliceosome activity) remains partially unresolved.

Response: We sincerely appreciate your valuable insights on the mechanism. In this study, we found that CCDC174 regulates the AS of maternal mRNAs by interacting with the PRP19/CDC5L spliceosomal complex. However, whether CCDC174 act as scaffold, regulate the activity of spliceosome, or have other unknown roles remains unclear, this deserves further investigation in future studies. Now we have added this point as a limitation in the discussion (Lines 391-395).

Relevance to infertility types

The study clearly establishes a role for CCDC174 in female infertility. There is no evidence for involvement in male infertility, which should be explicitly stated.

Response: According to your suggestion, we discussed the role of CCDC174 in male reproduction as following "In mice and *Drosophila*, numerous mRNA splicing regulators have been shown to be essential for male fertility, including PRP19/CDC5L complex members, BCAS2 and PRP19 (Li *et al*, 2025). Our study clearly demonstrates that CCDC174 functions as a key mRNA splicing regulator and interacts with the PRP19/CDC5L complex. Moreover, *Ccdc174* is highly expressed in mouse testes. Together, these findings suggest that CCDC174 may play an essential role in male reproduction. Nevertheless, the specific function of CCDC174 in spermatogenesis and its potential involvement in male infertility remains completely unknown and requires further elucidation" (Lines 309-316).

The manuscript provides a conceptual advance by establishing, for the first time, a direct link between a splicing regulator and human female reproductive disease. It complements and extends recent studies on BCAS2, ESRP1, and other post-transcriptional regulators of maternal mRNA homeostasis.

Response: We agree with your view and appreciate your positive comment.

Weaknesses and missing experiments

The main limitation is the lack of functional rescue experiments. In particular :

1. Lack of in vivo functional rescue experiments demonstrating that reintroduction of wild-type CCDC174 can restore oocyte maturation in knockout oocytes.

Response: According to your suggestion, we performed a functional rescue experiment. The results showed that reintroduction of wild-type CCDC174 into knockout GV oocytes failed to rescue the oocyte maturation arrest phenotype (Appendix Fig. S5). *Ccdc174* depletion severely disrupts the homeostasis of maternal mRNAs and proteins in oocytes. Affected molecules are involved in multiple key pathways governing oocyte development, such as GDF9 and BMP15 (essential factors regulating oocyte and follicle development). Disruption of these key factors and pathways cause irreversible damage to oocyte development. Therefore, reintroducing CCDC174 into GV oocytes cannot restore oocyte maturation. Furthermore, CCDC174 may begin to function in earlier growing oocytes. Depletion of *Ccdc174* causes irreversible damage in early growing oocytes, therefore, reintroduction of wild-type CCDC174 into knockout fully-grown oocytes cannot rescue the phenotype, however, reintroduction of wild-type CCDC174 into early growing oocytes is technically challenging (Lines 399-411).

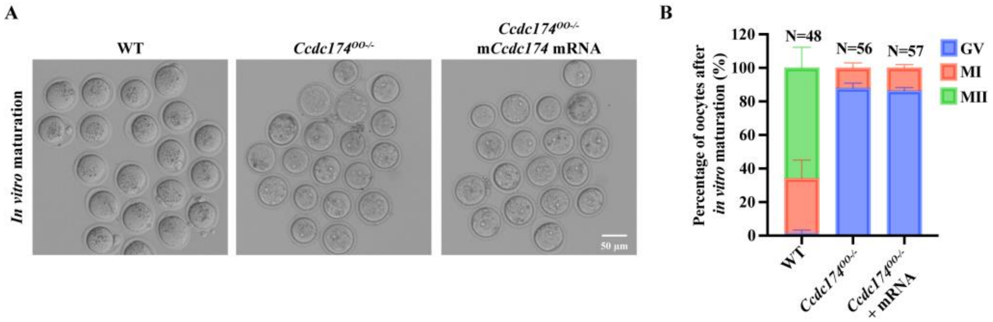

Appendix Fig. S5. Microinjection of mouse *Ccdc174* mRNA into *Ccdc174*$^{OO-/-}$ oocytes could not rescue oocyte maturation. (A) Representative images of oocytes after in vitro maturation (IVM). Scale bar = 50 μm. (B) The proportion of oocytes at different stages after IVM. The number of analyzed oocytes is indicated (N). *n* = 3 biological replicates.

2. The connection between specific alternative splicing defects and the observed cellular phenotypes remains largely correlative. The link could be obtained through identification and validation of at least one or two key target genes (e.g. involved in cell cycle regulation, DNA repair, or oocyte maturation) whose aberrant splicing is likely to account for the observed defects.

Response: In this study, we selected six genes (*Nobox*, *Cdc7*, *Clk1*, *Ddx47*, *Pabpc1l*, and *Nrf1*) for AS event detection and confirmed abnormal AS of these genes in *Ccdc174*$^{OO–/–}$ oocytes (Figure 5D), indicating that CCDC174 deficiency causes aberrant AS in oocytes. Notably, two of these genes are essential for oocyte development and female fertility: NOBOX is an oocyte-specific transcription factor required for oocyte-specific gene expression (Rajkovic *et al*, 2004), and PABPC1L regulates the translational activation of maternal mRNAs (Wang *et al*., 2023). Therefore, the aberrant AS of these key factors likely accounts for the oocyte competence defects observed in *Ccdc174*$^{OO–/–}$ oocytes. We have added this into the Result section (Lines 227-235).

3. Some mechanistic interpretations (e.g. compensatory upregulation of PRP19/CDC5L complex components) are plausible but not directly tested. These additions would significantly enhance the mechanistic depth expected for EMBO Mol Med.

Response: We agree with your point that the compensatory upregulation of the PRP19/CDC5L complex components are not directly tested, which need further confirmation in future. Although we currently lack direct experimental evidence to prove this interpretation, but at least, it demonstrated the imbalance of PRP19/CDC5L complex when CCDC174 was depleted. To be more stringent, we have added this point as a limitation in the Discussion section (Lines 395-397).

Referee #2 (Comments on Novelty/Model System for Author):
NONE
Referee #2 (Remarks for Author):

Oocyte competence defect is a common cause for IVF/ICSI failure, but the underlying mechanisms are largely unknown. In this work, Wang et al. identified homozygous or compound heterozygous variants in CCDC174 in five patients with oocyte competence defects. In vivo results using Ccdc174 knockout mouse showed that deletion of CCDC174 led to oocyte maturation arrest and infertility in female mice. They then used a series of experiments, including transcriptomic, proteomic, and IP-MS analyses, to find that CCDC174 regulates the alternative splicing of maternal mRNA through its interaction with the PRP19/CDC5L complex. Finally, they demonstrated that patient-derived variants are disruptive to protein function, including expression and binding to RNAs or CDC5L. This is the first study to reveal the

critical role of CCDC174 in human reproduction.

This study not only provides new insights into the molecular mechanism of oocyte quality control, but also provide a new diagnostic marker for clinical genetic counseling, interesting and highly innovative.

Response: Thank you for your positive assessment.

I have a few concerns as follows:

1. In the second paragraph of the Introduction, the authors first highlight the importance of maternal mRNA homeostasis for oocyte competence by introducing six reported mutant genes that regulate maternal mRNA metabolism. They then describe the important role of alternative splicing in regulating maternal mRNA homeostasis. It is important to note that the evidence for alternative splicing regulating maternal mRNA homeostasis is derived entirely from mice.

Response: Based on your suggestion, we have revised the relevant description as follows: Furthermore, knockout mouse studies demonstrated that alternative splicing (AS), a key step in post-transcriptional mRNA processing, is essential for maintaining maternal mRNA homeostasis and acquiring oocyte competence (Lines 68-71).

2. CCDC174 deficiency leads to abnormal mRNA splicing in mouse oocytes. If possible, it is recommended that the authors evaluate mRNA splicing in oocytes from patients with CCDC174 variants.

Response: Thank you for your constructive feedback. In this study, due to the lack of patient oocyte samples, we were unable to evaluate mRNA splicing in patient oocytes. We have acknowledged this limitation in the Discussion section (Lines 388-391).

3. For patients carrying the CCDC174 variants, what possible treatment strategies do the authors know that could improve their fertility in the future?

Response: Thank you for raising this important clinical question. Our findings identify CCDC174 as a novel pathogenic gene for oocyte competence defects. Nevertheless, treatment strategies for patients with *CCDC174* variants remain to be explored. We attempted to rescue the oocyte maturation arrest phenotype by microinjecting wild-type *Ccdc174* mRNA into GV oocytes from knockout mice, but this strategy proved unsuccessful (Figure S5). *Ccdc174* depletion severely disrupts the homeostasis of maternal mRNAs and proteins in oocytes. Affected molecules are involved in multiple key pathways governing oocyte

development, such as GDF9 and BMP15 (essential factors regulating oocyte and follicle development). Disruption of these key factors and pathways cause irreversible damage to oocyte development. Therefore, reintroducing CCDC174 into GV oocytes cannot restore oocyte maturation. Furthermore, CCDC174 may begin to function in earlier growing oocytes. Depletion of *Ccdc174* causes irreversible damage in early growing oocytes, therefore, reintroduction of wild type CCDC174 into knockout fully-grown oocytes cannot rescue the phenotype, however, reintroduction of wild type CCDC174 into early growing oocytes is technically challenging. Future studies could focus on targeting downstream effectors of CCDC174 or screening for small-molecule regulators of alternative splicing, which may provide possible treatment strategies to improve patient fertility. We have added this content to the Discussion section (Lines 397-414).

4, Patients with CCDC174 variants show phenotype of oocyte maturation, fertilization and embryonic defects. However, Ccdc174 knockout mouse show phenotype of oocyte GV arrest, please discuss the phenotypic difference. In addition, please also discuss how Ccdc174 dysfunction resulted in oocyte GV arrest.

Response: Based on your suggestion, we discussed the phenotypic differences between mice and patients in the manuscript as following: Compared to the complete loss of function resulting from gene knockout, the mutant CCDC174 retains partial biological activity that can support oocyte maturation but is insufficient for sustaining subsequent fertilization and embryonic development. Our functional studies further confirmed that the variants reduce the binding ability of CCDC174 with RNAs or with CDC5L rather than causing a complete loss of these functions. This further suggests that the phenotypic severity correlates with the degree of functional disruption in the protein (Lines 380-387).

In addition, we also discussed how CCDC174 dysfunction causes GV arrest in mice as following: The acquisition of oocyte competence relies on the precise regulation and synchronous completion of nuclear and cytoplasmic maturation (Conti & Franciosi, 2018). In this study, we demonstrated that CCDC174 deficiency alters the splicing pattern of a gene network comprising 1,018 genes. GO analysis revealed that these genes are essential for two maturation processes: nuclear maturation (e.g., cell cycle regulation, DNA damage and repair, chromatin organization and remodeling) and cytoplasmic maturation (e.g., mRNA processing and cytoskeletal organization). Additionally, transcriptomic

and proteomic analyses revealed aberrant homeostasis of stored mRNAs and proteins in *Ccdc174*$^{OO-/-}$ oocytes, which also disrupts cytoplasmic maturation. Overall, dual abnormalities in nuclear and cytoplasmic maturation severely impair the developmental competence of oocytes, ultimately resulting in GV arrest and female infertility in mice (Lines 344-355). We have added discussions in the revised manuscript.

5, Does CCDC174 also regulate AS in somatic cells, if yes, whether the patient have phenotypes in other somatic tissues?

Response: Studies have reported that CCDC174 interacts with EIF4A3 (a core component of the exon junction complex) in human neuroblastoma cells (Volodarsky *et al*, 2015), and interacts with NRDE2 to bind U1 snRNA and facilitate the processing of weak 5' splice sites in mouse ES cells (Flemr *et al*, 2023). These results suggest that CCDC174 may regulate splicing in somatic cells, although the detailed mechanism remains to be further elucidated.

According to the patient's clinical records, patients with *CCDC174* variants exhibited no obvious phenotypes in other somatic tissues. This oocyte-restricted phenotype could be explained by several mechanisms. One plausible mechanism is that *Ccdc174* is highly expressed in oocytes but lowly in somatic tissues (Figure 2), which likely renders oocytes more sensitive to *CCDC174* variants. In addition, genetic redundancy may contribute to this tissue-specific phenotype. In somatic cells, functionally redundant genes may compensate for impaired CCDC174 function, whereas such a compensatory mechanism is absent or insufficient in highly specialized oocytes. Furthermore, the unique process of oocyte meiosis requires strict temporal control over mRNA transcription and stability, which differs from the cell cycle and gene regulatory programs of somatic mitosis. These unique processes are likely more susceptible to dysfunction of CCDC174, leading to oocyte-specific phenotypes. We have added discussions in the revised manuscript (Lines 356-367).

Referee #3 (Comments on Novelty/Model System for Author):

In this study, the authors identified homozygous or compound heterozygous mutations in CCDC174 in five infertile patients presenting with oocyte competence defects. To date, the understanding of CCDC174's function is very limited, and its role specifically in reproduction remains completely unknown. Oocyte-specific knockout

mice confirmed in vivo that this gene is essential for oocyte development and female fertility. Through multi-omics analysis and diverse biochemical/molecular experiments, the authors revealed that CCDC174 regulates the AS and homeostasis of maternal mRNAs in oocytes via interaction with the PRP19/CDC5L complex. In vitro functional experiments demonstrated that patient-derived mutations impair the expression of CCDC174 or its binding ability to RNA/CDC5L.

This is an interesting and comprehensive study that is supported by both in vitro and in vivo experiments. The manuscript is well-written, and all experiments and results clearly described. This work is the first to report the physiological and pathological mechanisms of CCDC174 in oocyte development, providing novel diagnostic markers for female infertility patients with oocyte competence defects.

Response: Thank you for your positive evaluation of this study.

Referee #3 (Remarks for Author):

In this study, the authors identified homozygous or compound heterozygous mutations in CCDC174 in five infertile patients presenting with oocyte competence defects. To date, the understanding of CCDC174's function is very limited, and its role specifically in reproduction remains completely unknown. Oocyte-specific knockout mice confirmed in vivo that this gene is essential for oocyte development and female fertility. Through multi-omics analysis and diverse biochemical/molecular experiments, the authors revealed that CCDC174 regulates the AS and homeostasis of maternal mRNAs in oocytes via interaction with the PRP19/CDC5L complex. In vitro functional experiments demonstrated that patient-derived mutations impair the expression of CCDC174 or its binding ability to RNA/CDC5L.

This is an interesting and comprehensive study that is supported by both in vitro and in vivo experiments. The manuscript is well-written, and all experiments and results clearly described. This work is the first to report the physiological and pathological mechanisms of CCDC174 in oocyte development, providing novel diagnostic markers for female infertility patients with oocyte competence defects.

Response: Thank you for your positive assessment.

I have the following suggestions.

1. The oocytes of all five patients exhibited low developmental competence, which was specifically manifested as oocyte maturation arrest, fertilization failure, and embryonic arrest. What might be the possible reasons for the phenotypic heterogeneity among different patients?

Response: There are some possible explanations for phenotypic heterogeneity. Firstly, different types (e.g., impaired expression, CDC5L

binding, or RNA binding) and degrees (severe vs. mild) of CCDC174 dysfunction caused by different variants might lead to phenotypic heterogeneity in these patients. Secondly, genetic modifiers may also play an important role in regulating the function of mutant CCDC174 and promoting phenotypic heterogeneity. In addition to genetic factors, external factors such as lifestyle, environmental exposure, and assisted reproductive strategies may also influence the phenotypic characteristics of patients. We have added these sentences to the Discussion section (Lines 368-376).

2. In Figure 2A, the RNA level of Ccdc174 in mouse zygotes is also relatively high. Is this gene indispensable for early embryonic development, and will downregulating this gene in zygotes affect the early embryonic development of mice?

Response: Based on your suggestion, we designed siRNA targeting *Ccdc174* and confirmed significant downregulation of CCDC174 protein levels by immunoblotting (Figure R1A). Evaluation of embryonic development revealed that downregulation of CCDC174 in zygotes did not affect early embryonic development (Figure R1B, C). These results suggest that CCDC174 is not essential for early embryonic development in mice.

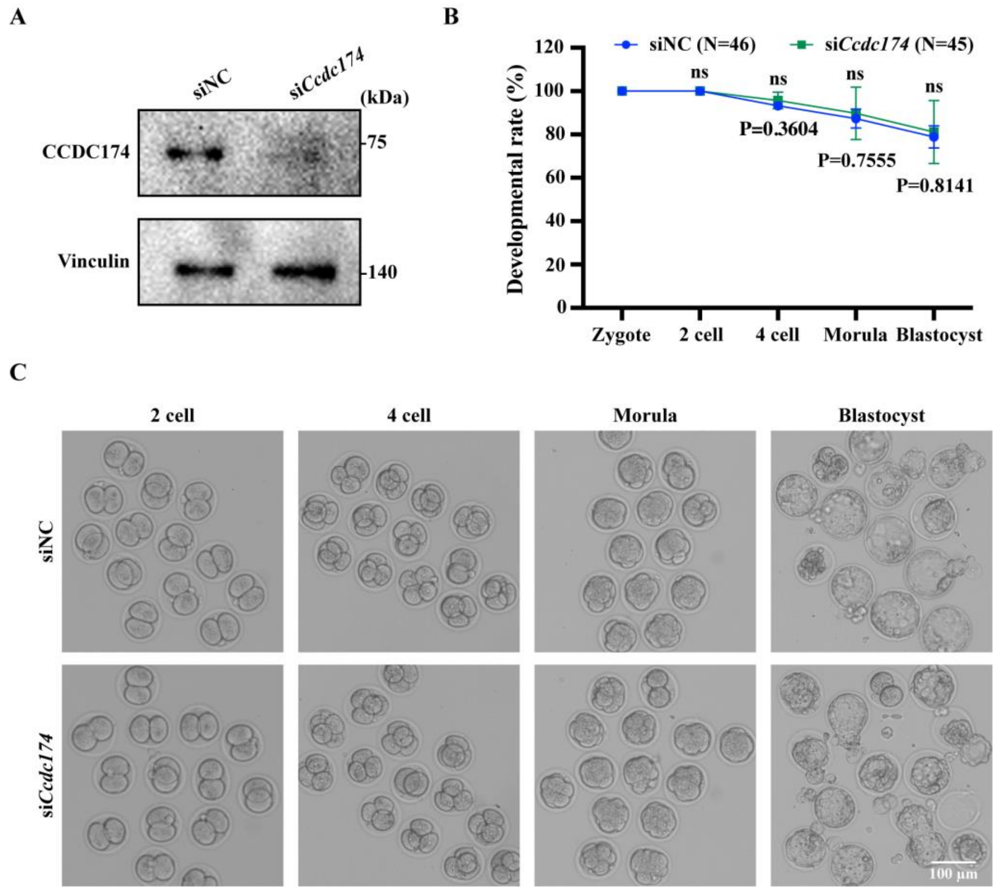

Figure R1 Downregulation of CCDC174 in zygotes does not affect early embryonic development in mice. (A) Immunoblotting shows that the CCDC174 level was significantly reduced by siRNA knockdown. (B) The proportion of embryos at different developmental stages. The number of analyzed embryos is indicated (N). n = 3 biological replicates. (C) Representative images of early embryonic development at different stages. Scale bar represents 100 μm.

3. In this study, why did the authors directly construct an oocyte-specific knockout mouse model instead of a global knockout mouse model? What is the phenotype of the knockout (KO) mouse? These key points need to be addressed by the authors in the manuscript.

Response: According to the Mouse Genome Informatics (MGI) database, homozygous *Ccdc174* knockout mice are embryonic lethality (MGI: 2444652). Therefore, to explore the function of CCDC174 in oocyte competence and female fertility, we directly generated an oocyte-specific knockout mouse model. We have added this information to the Results section of the manuscript (Lines 167-168).

4. If available, I suggest to detect the splicing events in patient oocytes.

Response: Thank you for your suggestion. In this study, due to the lack of patient oocyte samples, we were unable to detect the splicing events in patient oocytes. We have acknowledged this limitation in the Discussion section (Lines 388-391).

21st Apr 2026

Dear Prof. Sang,

Thank you for sending us your revised manuscript. We have now heard back from the two reviewers who were asked to re-evaluate your study. As you will see below, the reviewers are overall satisfied with the modifications made. Before we can formally accept your manuscript for publication, we would ask you to address the remaining minor issues raised by Reviewer #1.

Additionally, on a more editorial level:

1. Please remove "Authors' contribution" section.

2. Data availability:Please provide the specific URLs for CRA029850, OMIX011823,
OMIX011822 datasets.

3. Remove "data not shown"(line 332). As per our guidelines, on "Unpublished Data" the journal does not permit citation of "Data not shown". All data referred to in the paper should be displayed in the main or Expanded View figures.

4. Please change the heading "Materials and Methods" to "Methods".

5. Please address the following issues in figure legends:
- Please note that the exact p values are not provided in the legends of figures 3C, D, G; 5D, 7B, D, F;
- Please indicate the statistical test used for data analysis in the legends of figures 4B, C, E; 6A
- Please note that the measure of center for the error bars needs to be defined in the legend of figure 2A
- Please note that the dashed borders are not defined in the legend of figure EV1 A. This needs to be rectified.
- Please note that the white arrow heads are not defined in the legend of figure 3H. This needs to be rectified.

I look forward to seeing a revised form of your manuscript as soon as possible.

Kind regards,
Jingyi

Jingyi Hou
Senior Editor
EMBO Molecular Medicine

*** PLEASE NOTE *** As part of the EMBO Publications transparent editorial process initiative (see our Editorial at https://www.embopress.org/doi/pdf/10.1002/emmm.201000094), EMBO Molecular Medicine will publish online a Review Process File to accompany accepted manuscripts.

In the event of acceptance, this file will be published in conjunction with your paper and will include the anonymous referee reports, your point-by-point response and all pertinent correspondence relating to the manuscript. If you do NOT want this file to be published, please inform the editorial office at contact@embomolmed.org.

***** Reviewer's comments *****

Referee #1 (Comments on Novelty/Model System for Author):

See below

Referee #1 (Remarks for Author):

The revised manuscript by Wang et al. identifies biallelic variants in CCDC174 as a cause of human oocyte competence defects and female infertility, and proposes a mechanistic role for CCDC174 as a regulator of alternative splicing (AS) of maternal mRNAs via interaction with the PRP19/CDC5L spliceosomal complex. The study combines human genetics, an oocyte-specific knockout mouse model, multi-omics analyses, and molecular assays, and addresses an important unmet question in reproductive biology.

Overall assessment
The authors have adequately addressed the major concerns raised in the initial review. In particular:
A functional rescue experiment has now been performed. Although reintroduction of wild-type CCDC174 into knockout oocytes did not restore maturation, the authors provide a reasonable and biologically plausible explanation, supported by the extent of global transcriptomic and proteomic disruption and the likely requirement for earlier developmental expression.
The authors have strengthened the mechanistic link between splicing defects and phenotype by validating multiple alternatively spliced transcripts, including functionally relevant genes such as NOBOX and PABPC1L, which are known regulators of oocyte development and maternal mRNA regulation.
Potential overinterpretations regarding the PRP19/CDC5L complex have been appropriately moderated, and key limitations are now clearly acknowledged in the Discussion.
Additional clarifications regarding male fertility, phenotypic variability, and model differences between human patients and mice have improved the overall clarity and completeness of the manuscript.
Taken together, the revised version represents a substantial improvement over the initial submission.

Remaining minor points
The study is overall convincing, and no additional experiments are strictly required. However, a few minor points could be further clarified to strengthen the manuscript:
1-Rescue experiment interpretation
While the lack of phenotypic rescue is plausibly explained by early and irreversible defects, it would be helpful to more explicitly emphasize in the Discussion that this limitation prevents a definitive functional rescue conclusion and reflects the temporal requirement of CCDC174 during oocyte development.
2-Link between AS defects and phenotype
The validation of selected targets (e.g. NOBOX, PABPC1L) is appropriate and strengthens the study. However, it should be clearly stated that these represent illustrative examples within a broader network-level defect, rather than definitive single downstream effectors.
These points are minor and can be addressed in the text.

Conclusion
This study provides a novel and important conceptual advance, establishing for the first time a link between alternative splicing regulation and human female infertility. The genetic evidence, in vivo model, and multi-layered mechanistic analyses are strong and convincing.

I therefore recommend acceptance after minor revision.

Referee #3 (Comments on Novelty/Model System for Author):

In my opinion, the authors have sufficiently revised their manuscript.

Referee #3 (Remarks for Author):

The manuscript is now can be published based on my judgement.

***** Reviewer's comments *****

Referee #1 (Comments on Novelty/Model System for Author):
See below
Referee #1 (Remarks for Author):
The revised manuscript by Wang et al. identifies biallelic variants in CCDC174 as a cause of human oocyte competence defects and female infertility, and proposes a mechanistic role for CCDC174 as a regulator of alternative splicing (AS) of maternal mRNAs via interaction with the PRP19/CDC5L spliceosomal complex. The study combines human genetics, an oocyte-specific knockout mouse model, multi-omics analyses, and molecular assays, and addresses an important unmet question in reproductive biology.
Overall assessment
The authors have adequately addressed the major concerns raised in the initial review. In particular:
A functional rescue experiment has now been performed. Although reintroduction of wild-type CCDC174 into knockout oocytes did not restore maturation, the authors provide a reasonable and biologically plausible explanation, supported by the extent of global transcriptomic and proteomic disruption and the likely requirement for earlier developmental expression.
The authors have strengthened the mechanistic link between splicing defects and phenotype by validating multiple alternatively spliced transcripts, including functionally relevant genes such as NOBOX and PABPC1L, which are known regulators of oocyte development and maternal mRNA regulation.
Potential overinterpretations regarding the PRP19/CDC5L complex have been appropriately moderated, and key limitations are now clearly acknowledged in the Discussion.
Additional clarifications regarding male fertility, phenotypic variability, and model differences between human patients and mice have improved the overall clarity and completeness of the manuscript.
Taken together, the revised version represents a substantial improvement over the initial submission.
Response: We greatly appreciate your positive feedback.

Remaining minor points

The study is overall convincing, and no additional experiments are strictly required. However, a few minor points could be further clarified to strengthen the manuscript:
1-Rescue experiment interpretation

While the lack of phenotypic rescue is plausibly explained by early and irreversible defects, it would be helpful to more explicitly emphasize in the Discussion that this limitation prevents a definitive functional rescue conclusion and reflects the temporal requirement of CCDC174 during oocyte development.

Response: Thank you for your valuable suggestion. We have added the statement "This limitation prevents a definitive functional rescue conclusion and reflects the temporal requirement of CCDC174 during oocyte development" in the Discussion section (Lines 407-409).

2-Link between AS defects and phenotype

The validation of selected targets (e.g. NOBOX, PABPC1L) is appropriate and strengthens the study. However, it should be clearly stated that these represent illustrative examples within a broader network-level defect, rather than definitive single downstream effectors.

These points are minor and can be addressed in the text.

Response: According to your suggestion, we have clearly stated that these represent illustrative examples within broader network-level splicing defects, rather than definitive single downstream effectors (Lines 234-235).

Conclusion

This study provides a novel and important conceptual advance, establishing for the first time a link between alternative splicing regulation and human female infertility. The genetic evidence, in vivo model, and multi-layered mechanistic analyses are strong and convincing

I therefore recommend acceptance after minor revision.

Response: We greatly appreciate your positive comments and insightful suggestions, which have substantially improved our manuscript.

29th Apr 2026

Dear Prof. Sang,

We are pleased to inform you that your manuscript is accepted for publication and is now being sent to our publisher to be included in the next available issue of EMBO Molecular Medicine.

Your manuscript will be processed for publication by EMBO Press. It will be copy edited and you will receive page proofs prior to publication. Please note that you will be contacted by Springer Nature Author Services to complete licensing and payment information.

You may qualify for financial assistance for your publication charges - either via a Springer Nature fully open access agreement or an EMBO initiative. Check your eligibility: https://link.springer.com/journal/44321/how-to-publish-with-us

Should you be planning a Press Release on your article, please get in contact with embo_production@springernature.com as early as possible in order to coordinate publication and release dates.

If you have any questions, please do not hesitate to contact the Editorial Office. Thank you for your contribution to EMBO Molecular Medicine.

Yours sincerely,
Jingyi

Jingyi Hou
Senior Editor
EMBO Molecular Medicine

>>> Please note that it is EMBO Molecular Medicine policy for the transcript of the editorial process (containing referee reports and your response letter) to be published as an online supplement to each paper. If you do NOT want this, you will need to inform the Editorial Office via email immediately. More information is available here: https://link.springer.com/partners/embo-press/editorial-policies#Peer%20review

EMBO Press Author Checklist

Corresponding Author Name: Qing Sang, Lei Wang, Weijie Wang	
Journal Submitted to: EMBO Molecular Medicine	
Manuscript Number: EMM-2026-23245	

USEFUL LINKS FOR COMPLETING THIS FORM
The EMBO Journal - Author Guidelines
EMBO Reports - Author Guidelines
Molecular Systems Biology - Author Guidelines
EMBO Molecular Medicine - Author Guidelines

Reporting Checklist for Life Science Articles (updated January
This checklist is adapted from Materials Design Analysis Reporting (MDAR) Checklist for Authors. MDAR establishes a minimum set of requirements in transparent reporting in the life sciences (see Statement of Task: 10.31222/osf.io/9sm4x). Please follow the journal's guidelines in preparing your
Please note that a copy of this checklist will be published alongside your article.

Abridged guidelines for figures
1. Data
The data shown in figures should satisfy the following conditions:
- the data were obtained and processed according to the field's best practice and are presented to reflect the results of the experiments in an accurate and unbiased manner.
- ideally, figure panels should include only measurements that are directly comparable to each other and obtained with the same assay.
- plots include clearly labeled error bars for independent experiments and sample sizes. Unless justified, error bars should not be shown for technical
- if n<5, the individual data points from each experiment should be plotted. Any statistical test employed should be justified.
- Source Data should be included to report the data underlying figures according to the guidelines set out in the authorship guidelines on Data

2. Captions
Each figure caption should contain the following information, for each panel where they are relevant:
- a specification of the experimental system investigated (eg cell line, species name).
- the assay(s) and method(s) used to carry out the reported observations and measurements.
- an explicit mention of the biological and chemical entity(ies) that are being measured.
- an explicit mention of the biological and chemical entity(ies) that are altered/varied/perturbed in a controlled manner.
- the exact sample size (n) for each experimental group/condition, given as a number, not a range;
- a description of the sample collection allowing the reader to understand whether the samples represent technical or biological replicates (including how many animals, litters, cultures, etc.).
- a statement of how many times the experiment shown was independently replicated in the laboratory.
- definitions of statistical methods and measures:
  - common tests, such as t-test (please specify whether paired vs. unpaired), simple $\chi 2$ tests, Wilcoxon and Mann-Whitney tests, can be unambiguously identified by name only, but more complex techniques should be described in the methods section;
  - are tests one-sided or two-sided?
  - are there adjustments for multiple comparisons?
  - exact statistical test results, e.g., P values = x but not P values < x;
  - definition of 'center values' as median or average;
  - definition of error bars as s.d. or s.e.m.

Please complete ALL of the questions below.
Select "Not Applicable" only when the requested information is not relevant for your study.

Materials

Newly Created Materials	Information included in the manuscript?	In which section is the information available? (Reagents and Tools Table, Materials and Methods, Figures, Data Availability Section)
New materials and reagents need to be available; do any restrictions apply?	Yes	Materials and Methods

Antibodies	Information included in the manuscript?	In which section is the information available? (Reagents and Tools Table, Materials and Methods, Figures, Data Availability Section)
For **antibodies** provide the following information:<br>- Commercial antibodies: RRID (if possible) or supplier name, catalogue number and or/clone number<br>- Non-commercial: RRID or citation	Yes	Appendix Table S2

DNA and RNA sequences	Information included in the manuscript?	In which section is the information available? (Reagents and Tools Table, Materials and Methods, Figures, Data Availability Section)
**Short novel DNA or RNA including primers, probes**: provide the sequences.	Yes	Appendix Table S1

Cell materials	Information included in the manuscript?	In which section is the information available? (Reagents and Tools Table, Materials and Methods, Figures, Data Availability Section)
**Cell lines:** Provide species information, strain. Provide accession number in repository **OR** supplier name, catalog number, clone number, and/**OR** RRID.	Yes	Materials and Methods
**Primary cultures:** Provide species, strain, sex of origin, genetic modification status.	Not Applicable	
Report if the cell lines were recently **authenticated** (e.g., by STR profiling) and tested for mycoplasma contamination.	Not Applicable	

Experimental animals	Information included in the manuscript?	In which section is the information available? (Reagents and Tools Table, Materials and Methods, Figures, Data Availability Section)
**Laboratory animals or Model organisms:** Provide species, strain, sex, age, genetic modification status. Provide accession number in repository **OR** supplier name, catalog number, clone number, **OR** RRID.	Yes	Materials and Methods
**Animal observed in or captured from the field:** Provide species, sex, and age where possible.	Not Applicable	
Please detail **housing and husbandry conditions**.	Not Applicable	

Plants and microbes	Information included in the manuscript?	In which section is the information available? (Reagents and Tools Table, Materials and Methods, Figures, Data Availability Section)
**Plants:** provide species and strain, ecotype and cultivar where relevant, unique accession number if available, and source (including location for collected wild specimens).	Not Applicable	
**Microbes:** provide species and strain, unique accession number if available, and source.	Not Applicable	

Human research participants	Information included in the manuscript?	In which section is the information available? (Reagents and Tools Table, Materials and Methods, Figures, Data Availability Section)
If collected and within the bounds of privacy constraints report on age, sex and gender or ethnicity for all study participants.	Yes	Table 2

Core facilities	Information included in the manuscript?	In which section is the information available? (Reagents and Tools Table, Materials and Methods, Figures, Data Availability Section)
If your work benefited from core facilities, was their service mentioned in the acknowledgments section?	Not Applicable	

Design

Study protocol	Information included in the manuscript?	In which section is the information available? (Reagents and Tools Table, Materials and Methods, Figures, Data Availability Section)
If study protocol has been **pre-registered, provide DOI in the manuscript**. For clinical trials, provide the trial registration number **OR** cite DOI.	Not Applicable	
Report the **clinical trial registration number** (at ClinicalTrials.gov or equivalent), where applicable.	Not Applicable	

Laboratory protocol	Information included in the manuscript?	In which section is the information available? (Reagents and Tools Table, Materials and Methods, Figures, Data Availability Section)
Provide DOI OR other citation details if **external detailed step-by-step protocols** are available.	Not Applicable	

Experimental study design and statistics	Information included in the manuscript?	In which section is the information available? (Reagents and Tools Table, Materials and Methods, Figures, Data Availability Section)
Include a statement about **sample size** estimate even if no statistical methods were used.	Yes	Materials and Methods
Were any steps taken to minimize the effects of subjective bias when allocating animals/samples to treatment (e.g. **randomization procedure**)? If yes, have they been described?	Not Applicable	
Include a statement about **blinding** even if no blinding was done.	Not Applicable	
Describe **inclusion/exclusion criteria** if samples or animals were excluded from the analysis. Were the criteria pre-established? If sample or data points were omitted from analysis, report if this was due to attrition or intentional exclusion and provide justification.	Not Applicable	
For every figure, are **statistical tests** justified as appropriate? Do the data meet the assumptions of the tests (e.g., normal distribution)? Describe any methods used to assess it. Is there an estimate of variation within each group of data? Is the variance similar between the groups that are being statistically compared?	Yes	Materials and Methods, Figure legends

Sample definition and in-laboratory replication	Information included in the manuscript?	In which section is the information available? (Reagents and Tools Table, Materials and Methods, Figures, Data Availability Section)
In the figure legends: state number of times the experiment was **replicated** in laboratory.	Yes	Figure legends
In the figure legends: define whether data describe **technical or biological replicates**.	Yes	Figure legends

Ethics

Ethics	Information included in the manuscript?	In which section is the information available? (Reagents and Tools Table, Materials and Methods, Figures, Data Availability Section)
Studies involving **human participants**: State details of **authority granting ethics approval** (IRB or equivalent committee(s), provide reference number for approval.	Yes	Materials and Methods
Studies involving **human participants**: Include a statement confirming that **informed consent** was obtained from all subjects and that the experiments conformed to the principles set out in the WMA Declaration of Helsinki and the Department of Health and Human Services Belmont Report.	Yes	Materials and Methods
Studies involving **human participants:** For publication of **patient photos**, include a statement confirming that consent to publish was obtained.	Not Applicable	
Studies involving experimental **animals**: State details of **authority granting ethics approval** (IRB or equivalent committee(s), provide reference number for approval. Include a statement of compliance with ethical regulations.	Yes	Materials and Methods
Studies involving **specimen and field samples:** State if relevant **permits** obtained, provide details of authority approving study; if none were required, explain why.	Not Applicable	

Dual Use Research of Concern (DURC)	Information included in the manuscript?	In which section is the information available? (Reagents and Tools Table, Materials and Methods, Figures, Data Availability Section)
Could your study fall under dual use research restrictions? Please check biosecurity documents and list of **select agents and toxins** (CDC): https://www.selectagents.gov/sat/list.htm	Not Applicable	
If you used a select agent, is the security level of the lab appropriate and reported in the manuscript?	Not Applicable	
If a study is subject to dual use research of concern regulations, is the name of the **authority granting approval and reference number** for the regulatory approval provided in the manuscript?	Not Applicable	

Reporting

The MDAR framework recommends adoption of discipline-specific guidelines, established and endorsed through community initiatives. Journals have their own policy about requiring specific guidelines and recommendations to complement MDAR.

Adherence to community standards	Information included in the manuscript?	In which section is the information available? (Reagents and Tools Table, Materials and Methods, Figures, Data Availability Section)
State if relevant guidelines or checklists (e.g., **ICMJE, MIBBI, ARRIVE, PRISMA**) have been followed or provided.	Not Applicable	
For **tumor marker prognostic studies,** we recommend that you follow the **REMARK** reporting guidelines (see link list at top right). See author guidelines, under 'Reporting Guidelines'. Please confirm you have followed these guidelines.	Not Applicable	
For **phase II and III randomized controlled trials**, please refer to the **CONSORT** flow diagram (see link list at top right) and submit the CONSORT checklist (see link list at top right) with your submission. See author guidelines, under 'Reporting Guidelines'. Please confirm you have submitted this list.	Not Applicable	

Data Availability

Data availability	Information included in the manuscript?	In which section is the information available? (Reagents and Tools Table, Materials and Methods, Figures, Data Availability Section)
Have **primary datasets** been deposited according to the journal's guidelines (see 'Data Deposition' section) and the respective accession numbers provided in the Data Availability Section?	Yes	Materials and Methods
Were **human clinical and genomic datasets** deposited in a public access-controlled repository in accordance to ethical obligations to the patients and to the applicable consent agreement?	Not Applicable	
Are **computational models** that are central and integral to a study available without restrictions in a machine-readable form? Were the relevant accession numbers or links provided?	Not Applicable	
If publicly available data were reused, provide the respective **data citations in the reference list**.	Not Applicable	
